# The Challenge of Producing Skin Test Antigens with Minimal Resources Suitable for Human Application against a Neglected Tropical Disease; Leprosy

**DOI:** 10.1371/journal.pntd.0002791

**Published:** 2014-05-29

**Authors:** Becky L. Rivoire, Stephen TerLouw, Nathan A. Groathouse, Patrick J. Brennan

**Affiliations:** Department of Microbiology, Immunology, and Pathology, Colorado State University, Fort Collins, Colorado, United States of America; University of California San Diego School of Medicine, United States of America

## Abstract

True incidence of leprosy and its impact on transmission will not be understood until a tool is available to measure pre-symptomatic infection. Diagnosis of leprosy disease is currently based on clinical symptoms, which on average take 3–10 years to manifest. The fact that incidence, as defined by new case detection, equates with prevalence, i.e., registered cases, suggests that the cycle of transmission has not been fully intercepted by implementation of multiple drug therapy. This is supported by a high incidence of childhood leprosy. Epidemiological screening for pre-symptomatic leprosy in large endemic populations is required to facilitate targeted chemoprophylactic interventions. Such a test must be sensitive, specific, simple to administer, cost-effective, and easy to interpret. The intradermal skin test method that measures cell-mediated immunity was explored as the best option. Prior knowledge on skin testing of healthy subjects and leprosy patients with whole or partially fractionated *Mycobacterium leprae* bacilli, such as Lepromin or the Rees' or Convit' antigens, has established an acceptable safety and potency profile of these antigens. These data, along with immunoreactivity data, laid the foundation for two new leprosy skin test antigens, MLSA-LAM (*M. leprae* soluble antigen devoid of mycobacterial lipoglycans, primarily lipoarabinomannan) and MLCwA (*M. leprae* cell wall antigens). In the absence of commercial interest, the challenge was to develop these antigens under current good manufacturing practices in an acceptable local pilot facility and submit an Investigational New Drug to the Food and Drug Administration to allow a first-in-human phase I clinical trial.

## Author Summary

Despite reaching the global elimination target for leprosy, the need for a diagnostic tool to detect pre-symptomatic disease remains. Transmission has not been completely intercepted despite over 30 years of extensive curative treatment. With limited resources, two new leprosy skin test antigens, MLSA-LAM and MLCwA, suitable for human application were developed and manufactured in a local pilot plant. Requirements for manufacturing and clinical testing were met and an Investigational New Drug was established with the Food and Drug Administration to test both antigens in a phase I clinical trial for safety in a non-endemic region for leprosy and a phase II clinical trial for safety and efficacy in an endemic region for leprosy.

## Introduction

Detection of pre-symptomatic leprosy continues to be identified by the World Health Organization (WHO) as a priority [1]. With the introduction of multiple drug therapy (MDT) by the WHO in 1982 to subvert extensive resistance of *Mycobacterium leprae* resulting from years of dapsone monotherapy, the prevalence of leprosy began a dramatic decline [2]. Over the past 30 years, prevalence has dropped by about 98% from an estimated historical high of 11.5 million cases in 1983 [3] to the current figure of 192,246 registered cases [1]. Contrary to this remarkable achievement, leprosy incidence as defined by new case detection (NCD) remained relatively constant or increased slightly from 1985 at 555,188 new cases in the top 33 endemic countries [4] to 571,792 in 1990 [5] and 620,672 in 2002 [6]. A significant decline of 51.4% in new cases was then observed between 2002 and 2005 to 299,036, followed by another decline of 23.6% to the current figure of 228,474 [1]. A total decline of 58.8% in detection of new cases from 1985 to 2010 has been observed. Although many investigators have questioned the value of these numbers based on confounding operational factors [7], one fact remains; NCD has generally exceeded prevalence. Of added concern is the increased NCD of childhood leprosy signifying active and recent transmission of disease [8], [9]. These findings provide evidence that transmission of *M. leprae* from infected to susceptible individuals remains a problem.

Little is known of the extent of pre-symptomatic [10] leprosy in the endemic regions of the world, or reservoirs of infection, or bacterial or immunological basis of the distinctive pathogenesis of leprosy, notably nerve damage [11], [12]; however, we do know that early detection and treatment does reduce transmission [13] and disease sequelae [14]–[16]. Clinical leprosy is a bacteriological and pathological polar disease ranging from the tuberculoid (TT) to the lepromatous (LL) forms, with intermediate stages [17], [18]. This spectrum of disease is determined by the immunological status of the host [19]. The lepromatous forms are marked by high antibody titers, but T cell hyporesponsiveness (anergy) [20]; whereas the tuberculoid form show little evidence of *M. leprae* specific antibodies, but a vigorous Th1 response. Likewise, some household contacts (HC) of leprosy patients also demonstrate a specific T-cell response. These changes in the immune response along the continuum of disease suggest that a cell mediated immunity (CMI) test may be adequate to detect early leprosy infection.

Our approach toward development of an early diagnostic tool for leprosy has been focused on the delayed hypersensitivity (DTH) immune response, because it is considered to be sensitive, simple, cost-effective, and inexpensive when applied as Tuberculin Purified Protein Derivative (PPD), skin test for tuberculosis [21]. A DTH type IV reaction is initiated when antigen is injected into subcutaneous tissue and processed by antigen presenting cells. A Th_1_ effector cell recognizes the antigen and releases cytokines IL-2, IFN-γ, and TNF, which act on vascular endothelium causing erythema and recruitment of T-cells, phagocytes, fluid, and protein. This cascade of events causes a measurable induration response within 48–72 hours in humans. A lack of DTH response to recall antigen is evidence of anergy [22].

Early leprosy skin test studies with whole bacilli preparations, such as Lepromin-H (Mitsuda) [23] and Lepromin A [24], [25], had proven utility in classification of disease with the 21 day Mitsuda granulomatous reaction. However, the Lepromin antigen tends to prime the immune response and moreover is not specific for leprosy. Lepromin Dharmendra (Dharmendra) [26], Convit's Soluble Protein Antigen (SPA) or Leprosin, and Rees's *M. leprae* soluble antigen (MLSA) [27] evoked a 48 hour DTH reaction (the Fernandez reaction) [28]. TT leprosy patients had a characteristic DTH response to SPA and MLSA; whereas LL leprosy patients were anergic to these antigens, but not to other mycobacterial antigens such as PPD [29]. The DTH responses of borderline patients typically fell within the spectrum of their disease classification [30], [31]. Promising features of the MLSA and SPA included: neither had sensitizing potential [32]; both were potent immunologically; and, both were found to be safe in human vaccine trials in Venezuela, Malawi, and India [33], [34]. Shortcomings included inconsistent readings due to soft rather than hard DTH reaction in some individuals; variations in potency between batches due to quality control issues; and, lack of adequate sensitivity and specificity.

Two refined leprosy skin test antigens were identified [35]. The first antigen was a modified Rees antigen: MLSA-LAM (MLSA devoid of lipoglycans, primarily the immunosuppressive and cross-reactive lipoarabinomannan (LAM), and also lipomannan (LM), and phosphatidylinositol mannoside (PIM) and other lipids [36]–[38]). The second antigen was MLCwA (*M. leprae* cell wall antigen), consisting of the powerful immunogens of the cell wall devoid of lipoglycans [39],[40].

Active ingredients of these two intradermal skin test antigens are proteins of *M. leprae*. MLSA-LAM contains soluble protein antigens; over 100 individual proteins were initially recognized on two-dimensional gels, and about 30 of these had been sequenced and the immunological responses studied in part [41], [42]. Foremost among these antigens are the 70 kDa (DnaK), 65 kDa (GroEL), 45 kDa, 38 kDa, 35 kDa major membrane protein-I (MMP-I), 23 kDa superoxide dismutase (SOD), 18 kDa small heat shock protein (SmHSP), 18 kDa bacterioferritin (Bfr), 10 kDa (GroES), and the ribosomal proteins S7/S12 [43]–[48]. More recently, the full spectrum of proteins in soluble and insoluble subcellular fractions of *M. leprae* have been demonstrated and many more identified through the modern-day “proteomics” approach [49]–[51]. MLCwA contains many of the same proteins as MLSA-LAM, particularly the 70 kDa and 65 kDa and degradation products of these, the export/secretory proteins (notably the 30/31 kDa, multigene antigen 85 complex), and also some larger, uncharacterized proteins [50]. Details of the full spectrum of MLCwA constituent proteins have since been published [51].

Both leprosy antigens were chosen as skin test candidates based on adequate yield and biological justification with a robust DTH response in *M. leprae* sensitized compared to *M. tuberculosis* infected guinea pigs [52] and strong induction of lymphocyte proliferation and secretion of IFN-γ from TT leprosy patient immune cells [53], [54]. These early studies led to the development and manufacturing of these antigens [35]. The neglected tropical disease of leprosy is a disease of the poor, living in marginalized countries [55]; hence, commercial interest in the development of new products was lacking. Despite limited experience and resources, product development was implemented in this academic setting [56], The researchers overcame the challenges of developing and manufacturing skin test antigens suitable for human application [57].

## Methods

### Ethics Statement

Protocols for animal use were reviewed and approved by the Animal Care and Use Committee (ACUC) at Florida Institute of Technology (FIT) and Colorado State University (CSU). The CSU approved ACUC protocol number was 02-167A-02. The FIT Armadillo Facility was in-compliance with United States Department of Agriculture-American Public Health Association (USDA-APHA), United States Public Health Service-Office for Protection from Research Risks (USPHS-OPRR), and International ACUC (IACUC) standards. The CSU Laboratory Animal Facility followed IACUC regulations and guidelines. The Phase I trial (registration number: NCT01920750) and phase II trial (registration number: NCT00128193) were registered with ClinicalTrials.gov. The phase I trial was not registered prior to implementation, because the trial was completed (February, 1999), before ClinicalTrials.gov registry was made available to the public (February, 2000). Retrospective registration of the phase I trial was requested for publication. The clinical Phase I Protocol, **Protocol S1**, and Phase II Protocol, **Protocol S2**, are attached as **Supportive Information**; although details of the clinical study will follow in subsequent articles.

### Propagation of *M. leprae* in Armadillos

*M. leprae* cannot grow axenically, but can be propagated in the nine-banded armadillo, *Dasypus novemcinctus* [58]. At the Florida Institute of Technology (FIT), Melbourne, Florida, Eleanor. E. Storrs and subsequently Arvind Dhople et al. under National Institutes of Health (NIH), National Institutes of Allergy and Infectious Diseases (NIAID) with regard to support and authorization, captured armadillos from state or nationally managed land areas in Central Florida for propagation of *M. leprae*. Animals were treated for parasites, quarantined, and tested prior to release by: 1) acid fast staining of ear snips, nasal swabs, and blood for evidence of acid-fast bacilli; 2) culturing of blood samples for sterility in Trypticase Soy Broth and thioglycollate broth; 3) hematology; 4) serodiagnosis for IgM antibodies to phenolic glycolipid-I; and, 5) Lepromin test to determine susceptibility to *M. leprae* [58], [59].

The source of *M. leprae* was a untreated lepromatous leprosy individual from Guyana with large numbers of highly bacilliferous subcutaneous nodules and lepromas. Genetic evidence has since indicated that *M. leprae* isolates are antigenically homogeneous [60], [61]. Infected armadillos were sacrificed and the livers and spleens were homogenized and fractionated to separate *M. leprae* bacilli to serve as the Master Seed Stock in 2 ml volumes (3×10^8^ bacilli/ml) frozen at −70°C. Subsequently infected armadillos with disseminated leprosy were sacrificed and the tissues (liver and spleen), aseptically removed. The infected armadillo tissues were shipped to the Pilot Plant Skin Test Antigen Facility at CSU.

### Tissue Fractionation

**Figure 1:**
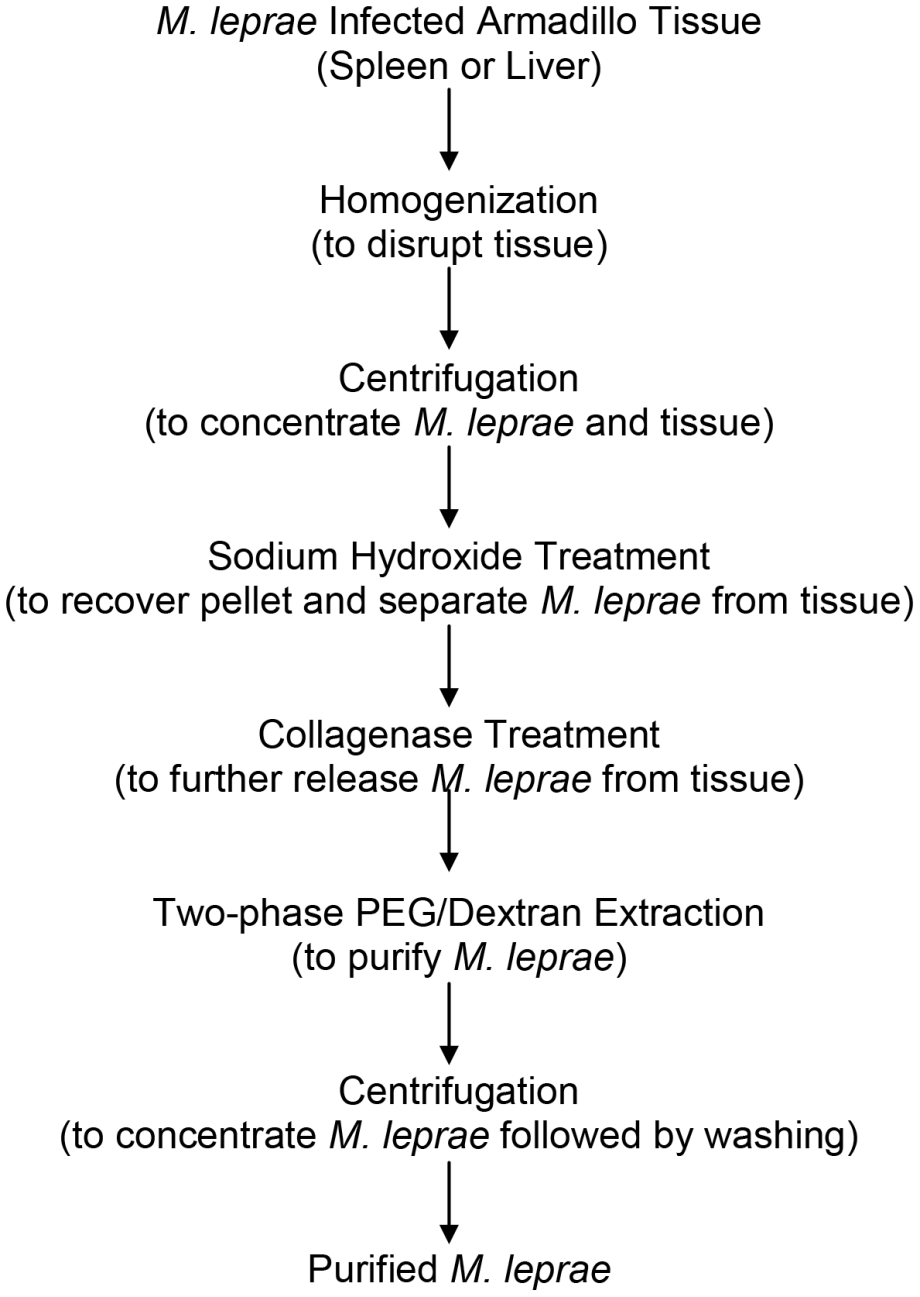
Tissue fractionation flow chart. *M. leprae* was purified from the tissues of experimentally infected armadillos. A total of seven batches were prepared to generate an adequate quantity of bacteria (128.4 mg) for bacterial fractionation.

A total of 242 g of *M. leprae* infected tissue (spleen, 19 g; liver, 223 g) from three infected armadillos [animal nos. A563 (19 g spleen, one preparation), A572 (109 g liver, divided into three preparations), and A581 (114 g liver, divided into three preparations)] were fractionated using a modified 3/77 Draper protocol [62] (**Figure 1**), except for omission of the step involving protease digestion with chymotrypsin and trypsin and alterations in buffer composition. Protease digestion of homogenate was removed since no difference was seen between treated and untreated tissue preparations in terms of purity, protein content, and immunological potency of the recovered *M. leprae*.

Tissue sections ranging from 19 g to 36.5 g were homogenized with 10 mM disodium ethylenediaminetetraacetic acid (EDTA, Sigma, St. Louis, Mo.), pH 8.0 at 3 ml/g of tissue. Homogenates were tested for sterility on brain heart infusion agar, blood agar, and Lowenstein-Jensen agar (BD, Franklin Lakes, NJ). Tissue fragments were pelleted and washed twice with 10 mM EDTA by centrifugation (Sorvall RC5, Thermo Fisher Scientific, Inc., Rockford, IL) at 15,000× g for 10 min at 4°C in 50 ml Teflon Oakridge tubes, followed by extraction with 0.1 M sodium hydroxide (Mallinckrodt Baker Inc., Phillipsburg, NJ) in 10 mM EDTA while stirring at room temperature for 2 h to remove pigment and to separate *M. leprae* from tissue. The suspension was pelleted and washed twice with 0.1 mM sodium phosphate/0.1% Tween 80 (Mallinckrodt/Fisher) followed by digestion with 20 mg collagenase (Sigma, St. Louis, Mo.) and 0.23 mM calcium chloride (Sigma) in 200 ml of the sodium phosphate Tween 80 buffer while stirring overnight at 37°C. The digest was again pelleted and washed prior to two-phase extraction with 6% polyethylene glycol 6,000 and 8% Dextran T-500 (Sigma) in 0.1 M sodium phosphate/150 mM sodium chloride at 10 ml/g of tissue in a separatory funnel. The upper phase containing bacteria was removed and an equal volume of 0.2% Tween 80 added prior to centrifugation at 27,000× g for 30 min at 4°C. Purified *M. leprae* was washed twice at 15,000× g with buffered water and the concentration of bacilli estimated with a 1∶100 and 1∶200 dilution by optical density at A_540_ using an empirically determined conversion factor of 0.362 based on dry weight, i.e., A_540_ of 1.0 = 0.362 mg *M. leprae*/ml multiplied by the dilution factor. Samples of the bacilli were tested for sterility by culturing on brain heart infusion agar, blood agar, and Lowenstein-Jensen agar. Purity was subjectively determined by acid fast staining using methlyene blue as a counterstain for residual tissue, with acceptance criteria of ≥90% [63], [64].

### Bacterial Fractionation

**Figure 2:**
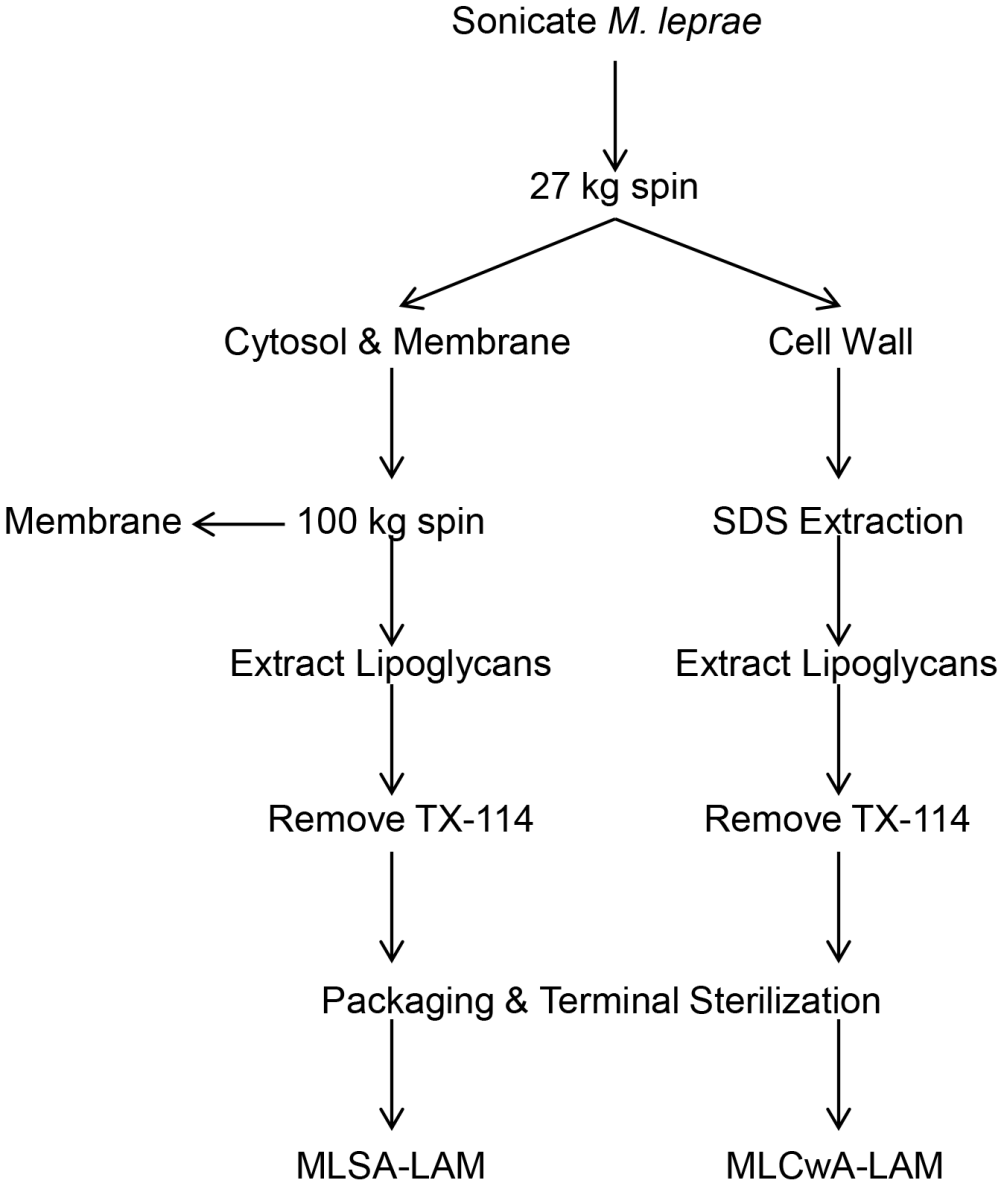
Bacterial fractionation flow chart. Bacteria were sonicated and fractionated into subcellular components: cell wall, cytosol, and membrane. The membrane antigen preparation was not further pursued. Cell wall associated proteins were extracted with SDS and both the cytosol and cell wall fractions were then extracted with TX-114 to remove non-specific hyporesponsive antigens, mostly LAM, LM, and PIM's. Residual detergent was removed by affinity chromatography. Antigens were diluted to prescribed concentrations, vialed, labeled, and autoclaved.

*M. leprae* (128.43 mg) from seven such preparations were pooled and washed twice with 25 ml phosphate buffered saline (PBS) by centrifugation at 27,000× g for 15 min at 4°C (**Figure 2**). Bacteria were suspended in 5 ml PBS and disrupted by sonication on cold packs with an ultrasonic processor (Sanyo Soniprep 150, MSE Ltd., Lower Sydenham, London) at 1.5 MHz, 50% duty, and 1 second pulse intervals over six 5 min cycles with 5 min cooling between each cycle. Pre and post-sonicated bacteria were stained using the TB Acid Fast Stain Kit (Thermo Fisher Scientific Inc.) for counting to verify greater than 80% breakage.

Disrupted bacteria were centrifuged at 27,000× g for 30 min. Supernatant consisting of cytosol and membrane was transferred to a fresh tube and centrifugation repeated. The pellet consisting of *M. leprae* cell wall was washed three times with 10 ml PBS. The cytosol/membrane containing supernatant was transferred to an Ultra Clear 5 ml (13×51 mm) tube and ultracentrifuged (Optima TLX 120, Beckman Coulter Inc., Brea, CA) at 100,000× g for 2 h at 4°C to pellet the membrane. To remove lipoglycans [65] cold 20% condensed Triton X-114 (Baxter, Deerfield, IL) was added to the supernatant (cytosol) to a final concentration of 4%, followed by rocking at 4°C overnight. The tube was placed in a beaker of water at 37°C for 10 min to condense the Triton X-114 followed by centrifugation for 15 min at 3,900× g at 22°C to separate detergent and aqueous layers. The top layer was transferred onto tandem 1 ml Extracti-gel D (Fisher) columns to remove residual detergent. Extraction and removal of residual detergent was then repeated.

Cell wall pellet was resuspended with 2 ml of 2% sodium dodecyl sulfate (SDS, Fisher)/PBS and stirred while heating at 56°C for 1 h followed by centrifugation for 15 min at 27,000× g at 22°C to remove the SDS solubilized *M. leprae* cell wall antigens; the residual *M. leprae* cell walls consisting of the mycolylarabinogalactan-peptidoglycan complex has been the subject of much research [66], [67]. The supernatant was transferred to a fresh tube and the extraction was repeated. The MLCwA preparation was passed over two 1 ml Extracti-gel D columns to remove residual SDS and finally subjected to two rounds of TX-114 extraction followed by removal of residual detergent as described above.

**Figure 3:**
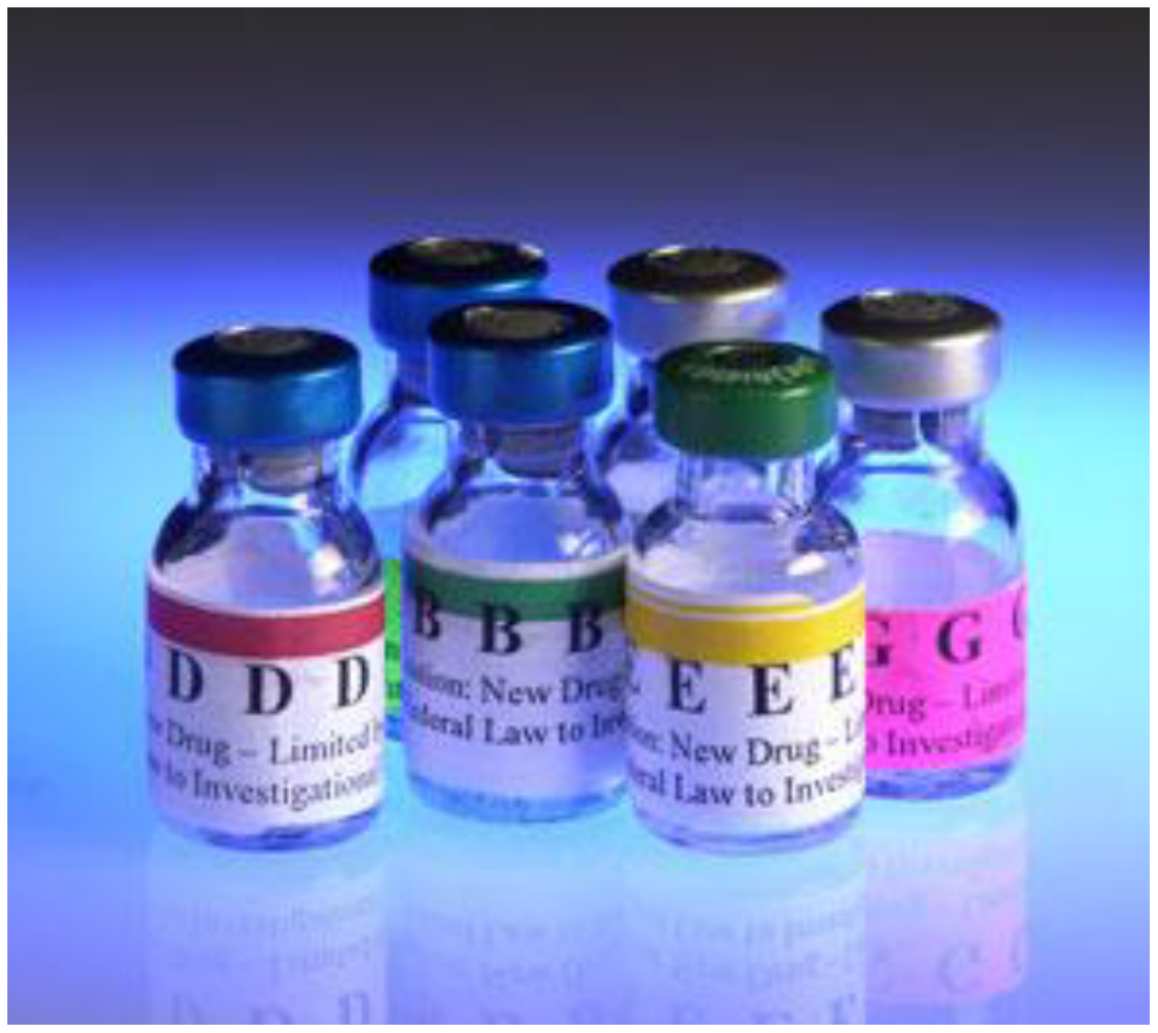
Packaged and labeled leprosy skin test antigens. Product interventions, MLSA-LAM and MLCwA, each at 1.0 µg and 0.1 µg, and the control antigens, saline and Tuberculin 5 TU, were coded by Fisher Bioservices Repository prior to shipment to the phase II clinical site for blinded applications.

The protein concentration of each of the antigen preparations was assessed by the Bicinchoninic Acid assay (Fisher). Antigens were diluted with PBS containing 0.0005% Tween 80 to a final dosage of 10.0, 5.0, 2.5, 1.0, and 0.1 µg protein per 0.1 ml followed by 0.2 µm filtration to remove residual particulates. A total of 1 ml of each of the antigen doses was aliquoted into prewashed and sterilized 2 ml borosilicate vials with 13 mm silicon rubber stoppers and aluminum caps (Wheaton, Millville, NJ). Vials were labeled in accordance with Food and Drug Administration (FDA) labeling requirements, including the statement, “Caution: New Drug-Limited by Federal Law to Investigational Use” [68], autoclaved for 20 min at 121°C; cooled at room temperature, and placed at −70°C for storage as MLSA-LAM and MLCwA batch no. 23 and lot no. 051297. Vials used in the phase I clinical trial remained at CSU, while those used in the phase II clinical trial were sent to Fisher Bioservices Repository (Rockville, MD) for relabeling with randomly assigned codes and shipment to the phase II clinical site (**Figure 3**).

### Quality Control of Antigens

#### Residual collagenase assay

A collagenase enzymatic assay adapted from Sigma was used to test for residual collagenase in skin test antigen preparations [69], [70]. A single unit of collagenase liberates 1 µmole of 4-phenylazobenzyloxycarbonyl (Pz)-Pro-Leu from the substrate Pz-Pro-Leu-Gly-Pro-dArg in 15 min at pH 7.1 at 37°C [71]. Collagenase assay sensitivity was 2.0 µg/ml.

#### Residual SDS assay

Residual SDS was measured by the Anionic Detergent Assay using methylene blue and chloroform [72], [73]. SDS Assay sensitivity was 5 ng/ml SDS.

#### Residual Triton X-114 assay

Residual Triton X-114 was measured by the Nonionic Detergent Assay using dichloromethane and cobaltothiocyanate reagent [74]. Triton X-114 assay sensitivity was 4 µg/ml.

#### Evaluation of protein and soluble carbohydrate identity

Prior to dose formulation, a sample was removed from each antigen to evaluate the protein profile by separation of proteins on reduced 15% polyacrylamide gel electrophoresis gels [75] and staining with silver nitrate to detect proteins [76] or sliver nitrate with periodate to detect glycans [77]. Antigens were loaded onto gels at 1.0, 0.5, 0.25, 0.1, and 0.01 µg per lane. *M. leprae* whole sonicate (2 µg) was used as a reference standard. Antigens were transferred to nitrocellulose in Tris, glycine, methanol transfer buffer for 1 h at 50 V [78]. Nitrocellulose panels were blocked with 1% Bovine Serum Albumin (Sigma) in Tris buffered saline (TBS)/0.05% Tween 80 as diluent for 1 h at room temperature and then incubated in one of the following primary antibodies for 1 h at room temperature: mouse monoclonal antibody (mab) anti-LAM (CS-35), mab anti-GroES (CS-01), mab anti-SOD (CS-18), mab anti-MMP-I (CS-38), mab anti-GroEL (CS-43), and rabbit polyclonal antibody against non-infected armadillo liver. All antibodies were prepared in-house. After washing three times in TBS/0.05% Tween 80, a dilution of goat anti-mouse IgG or goat anti-rabbit IgG conjugated to alkaline phosphatase were added to each panel followed by incubation for 1 h at room temperature. Panels were washed 3 times with TBS and once with water prior to developing in NBT-BCIP substrate (Sigma) for approximately 3 min before stopping the reaction with water.

#### General Sterility Test

The General Sterility Test procedure specified in Title 21 of the Code of Federal Regulations (CFR) Part 610.12 was performed [79].

#### General Safety Test

The General Safety Test procedure specified in 21 CFR 610.11 was performed in mice and guinea pigs with the 5.0 µg/0.1 ml dosage of each antigen preparation [80].

#### Assay for endotoxin content

The Limulus Amebocyte Lysate third generation pyrogen test from BioWhittaker, License No. 709 was used to test for endotoxin content [81]. Endotoxin assay sensitivity was 0.1 EU/ml.

#### DTH guinea pig potency assay

Guinea pigs of the outbred Hartley strain were sensitized by subcutaneous injection in the base of the neck with *M. leprae* inactivated at 80°C and suspended in Freund's Incomplete Adjuvant. After 4–8 weeks, 0.1 ml of the skin test antigens were administered intradermally on the freshly shaven back of each animal. Induration was measured at 24 and 48 hours post-injection [52].

#### General stability test

Skin test antigen batch no. 9, prepared in the general research laboratory was used for preliminary stability testing. Each antigen was diluted with PBS or borate buffer to a dosage of 10.0 µg/0.1 ml and either filtered or filtered and terminally sterilized. Immediately after packaging, each sample was placed at −70°C, 4°C, 37°C, or 56°C. Samples were analyzed for stability in the DTH Guinea Pig Potency Assay on days 45, 90, 120, and 360 at 1.0 µg and 0.1 µg doses. Abbreviated stability testing was performed on the cGMP batch no. 23, lot no. 051297, whereby antigens vialed at doses of 1.0 µg and 0.1 µg were tested at 4°C and 20°C against equivalent antigens stored at −70°C for 90 d, 120 d, 360 d, 2 y and 4 y.

#### Adventitious agent (virus) testing

Liver homogenates from each tissue fractionation and MLSA-LAM and MLCwA final products at 10.0 and 5.0 µg/0.1 ml were tested for human viral pathogens using cell based assays and polymerase chain reaction (PCR). Viral identification by cytopathic effect for adenovirus; parainfluenza 1, 2, and 3 viruses; influenza virus; poliovirus; cytomegalovirus; herpessimplex 1, and 2 viruses; and respiratory syncytial virus was conducted at the University of Colorado Diagnostic Virology Laboratory (Boulder, Colorado). PCR for hepatitis B virus and human immunodeficiency virus was performed by Specialty Laboratories, Inc. [82].

## Results

Product development began in 1992 with the immediate challenges of acquiring adequate expertise and funding, generally offered by an industrial partner. While maintaining a focus on the need for an early diagnostic test for leprosy, primary resources including regulatory, technical, and financial support were identified through government, professional, and industry contacts. Establishing a product development plan was also difficult, since the Product Development Roadmap [83] or FDA Translational Critical Path [84] had not yet been published, and experience with the complicated process was mostly found within pharmaceutical and biotechnology companies. To overcome these hurdles, regulatory and technical assistance were provided by NIH, NIAID, Division of Microbiology and Infectious Diseases (DMID) Regulatory Affairs Specialists, FDA representatives, professional organizations including the Parenteral Drug Association (PDA) and International Society for Pharmaceutical Engineering (ISPE), and quality system consultants. Finally, the mind-set in the research environment required a change from innovation to standardization to develop these two new antigens.

### cGMP Pilot Plant

**Figure 4:**
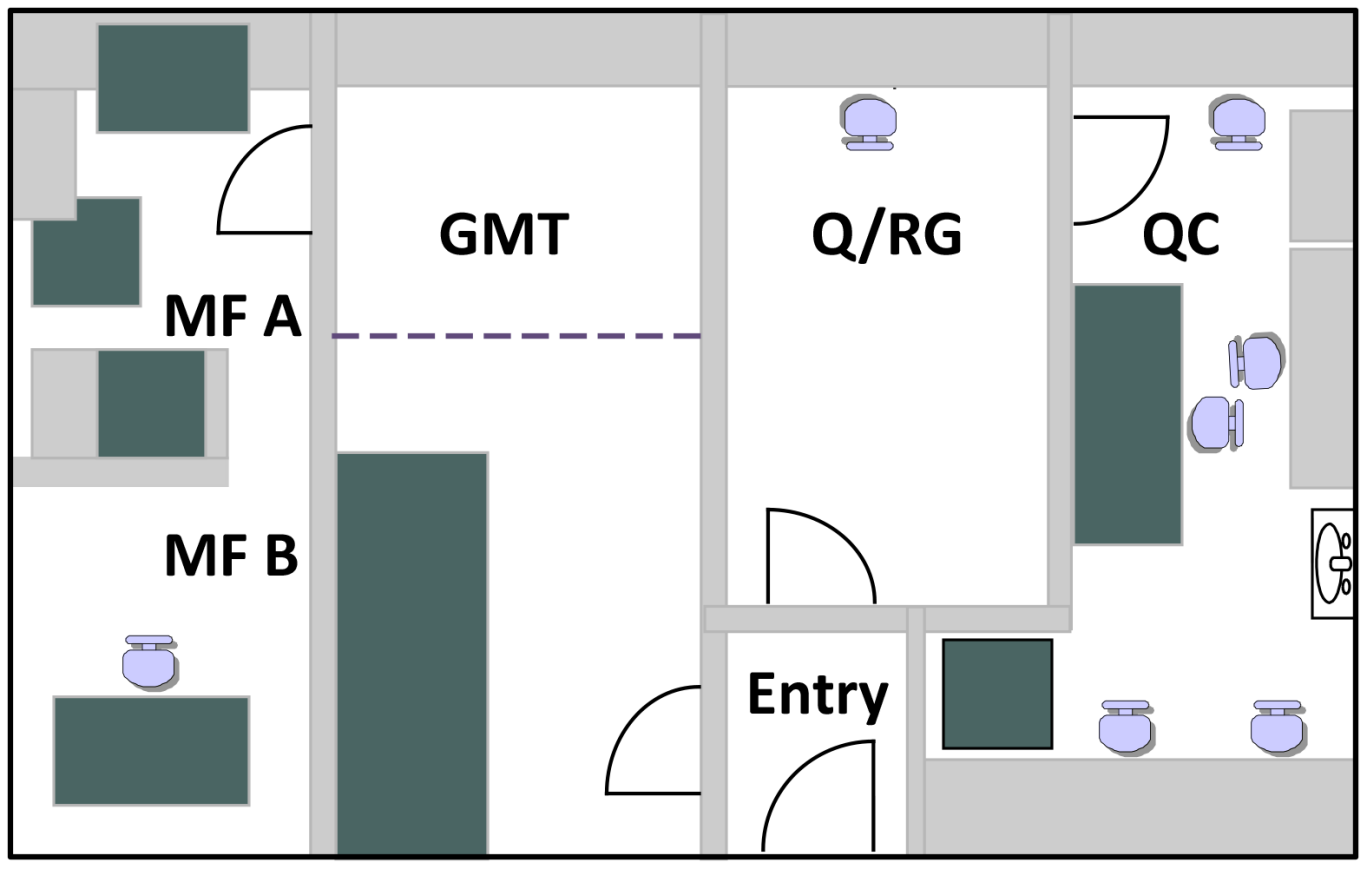
Leprosy skin test antigen pilot facility. In preparation for manufacturing, the five room cGMP suite consisted of: a Gowning and Material Transfer (GMT) ISO8 clean room; a Manufacturing Suite A (MF A) ISO7 clean room; a Manufacturing Suite B (MF B) ISO7 clean room; a Quarantine/Release Goods Room (Q/RG) clean/non-classified clean room; and, a Quality Control Laboratory (QC) ISO8 clean room.

Options for manufacturing the two new leprosy skin test antigens under cGMP, suitable for human application, were limited. Costs for using a contract manufacturing organization (CMO) were prohibitive; it was difficult to find any with an open schedule, and few had biosafety level 2 (BSL-2)/cGMP clean rooms required for safe manufacturing of these antigens. In addition, service providers acknowledged that they were fearful of working with *M. leprae*. Consequently, a retired BSL-3 research laboratory was converted to a cGMP Pilot Facility (**Figure 4**) at CSU for the sole purpose of manufacturing these leprosy skin test antigens. To this end, the manufacturing and testing process for MLSA-LAM and MLCwA was developed to meet 21 CFR parts 210, 211 for current Good Manufacturing Practices (cGMP) [85], [86]. Details of Pilot Plant Facility renovation are available from the authors.

The Pilot Facility consisted of a suite of five rooms, 1) Gowning and Material Transfer Room, 2) Manufacturing Suite A, 3) Manufacturing Suite B, 4) Quarantine/Released Goods Room, and 5) Quality Control Laboratory. Both the manufacturing and quality control rooms were under positive pressure cascading from the innermost room to the entry foyer. Air was supplied by a dedicated heating ventilation air conditioning system with single pass air flow monitored with gauges in the entry room and an anemometer prior to entry of the manufacturing suite. High efficiency particulate air filters were positioned on both the supply and exhaust air streams to purify air entering and exiting the clean rooms. The manufacturing rooms were classified [87] as international standard organization (ISO) 7 clean rooms. The innermost manufacturing room was used for downstream processing (antigen purification, formulation, and vialing), while the outermost manufacturing room was used for upstream processing (tissue fractionation and bacteria sonication). The gowning and material handling room was classified as an ISO8 clean room for personnel aseptic Tyvek gowning, wipe down and transfer of materials and equipment into the manufacturing area, and entering and exiting of personnel. The innermost quality control room, an ISO8 clean room was used for testing raw materials, intermediate product, and final product, while the quarantine/released goods room was a clean, non-classified clean room used for quarantine and release of raw materials.

Commissioning of the cGMP Pilot Plant for manufacturing skin test antigens was performed. Rooms were decontaminated with para-formaldehyde. The Pilot Plant was cleaned and the environment was monitored on three consecutive days and three consecutive weeks following directive documents to assess the cleanliness of the facility. Monitoring viable airborne organisms was performed with the Rotary Centrifugal Air Sampler (Biotest Diagnostics, Brooklyn Park, MN) and settling plates, both using Trypticase Soy Agar strips/plates. Monitoring viable surface organisms was performed with Rodac plates containing Trypticase Soy Agar and neutralizer for cleaning agents. Isolates were identified to the genius and species level using API Test Kits (Biomerieux, Etolile, France; distributed by VWR). Total particle counts in each clean room were measured using a Particle Counter (Metone Instruments, Grants Pass, Oregon). Acceptance criteria were met with each test enabling release of the Pilot Plant for cGMP manufacturing.

### Quality Management System

A quality system [88] was created for processing and testing leprosy skin test antigens in the renovated pilot plant [89]. The documentation system addressed: facility and equipment, materials, production, product labeling, laboratory control, and quality [90]. Two batch records were written, one for fractionation of tissues and the other for bacteria. A total of 255 supporting standard operating procedures (SOPs) were written to cover the quality system and manufacture of antigens. Facility and equipment SOPs were written for operation, maintenance, and calibration of dedicated equipment. SOPs for directing and tracking the chain of custody for raw materials transferred through purchasing, receiving, quarantine, release, and storage were created. Process directives supporting environmental monitoring, gowning, transferring material, manufacturing, in-process testing, and release testing were written into SOP format with data forms to collect relevant information. Explicit details for product labeling were captured in the batch record. All levels of training, including equipment use, biosafety, good laboratory practice (GLP), cGMP, and good clinical practice (GCP) were directed through SOPs. Logs were created to track part numbers, documents, raw materials, sample submission, equipment and room usage. Documents were subjected to the mandated review and approval process prior to implementation [91].

### Antigen Manufacturing and Testing

The manufacture of antigens was a two step process beginning with receipt, tracking, and release of raw materials. The primary raw material was spleen and liver tissues laden with *M. leprae* propagated in armadillos at FIT. Upon aseptic harvest, tissues were tested for the presence of contaminating bacteria using microbiological medium and then sent to the Pilot Plant, where they were frozen at −70°C in a qualified freezer until use. Manufacturing reagents were United States Pharmacopeia grade or equivalent, if available; otherwise, the highest purity was specified. Each reagent was released for use based on a certificate of analysis provided by the vendor, per an approved in-house specification sheet. Materials were tracked using a receiving code and part number system.

Tissue fractionation under the respective batch record was performed to release and purify *M. leprae* from armadillo tissue. A total of seven tissue runs were performed to accumulate 100–150 mg bacteria. Tissue weights ranged from 19–36.5 g for manageability and to maximize yields. A total of 128.4 mg of *M. leprae* was purified from 242 g tissue, resulting in a yield of 0.05% (**Table 1**). Sterility testing was performed on each bacterial lot, and material was stored at −70°C until use. Bacterial fractionation under the respective batch record was performed using the pooled intermediate product. Totals of 4.6 mg of MLSA-LAM and 5.0 mg of MLCwA were obtained, representing a yield of 3.57% and 3.88% from intact bacteria, respectively.

**Table 1:** Leprosy skin test antigen purification yields.

Step	Starting Material	Tissue	Animal No. (tissue wt)	Total Yield	Percent of prior step
1	Tissue	Spleen	A563 (19 g)	---	---
	Tissue	Liver	A572 (109 g)^a^	---	---
	Tissue	Liver	A581 (114 g)^a^	242.0 g	---
2	*M. leprae*	---	---	128.4 mg	0.05%
3	MLSA-LAM	---	---	4.6 mg	3.57%
	MLCwA	---	---	5.0 mg	3.88%

a Liver tissues were divided into three sections with an average weight of 32 g±0.9 g/run.

**Figure 5:**
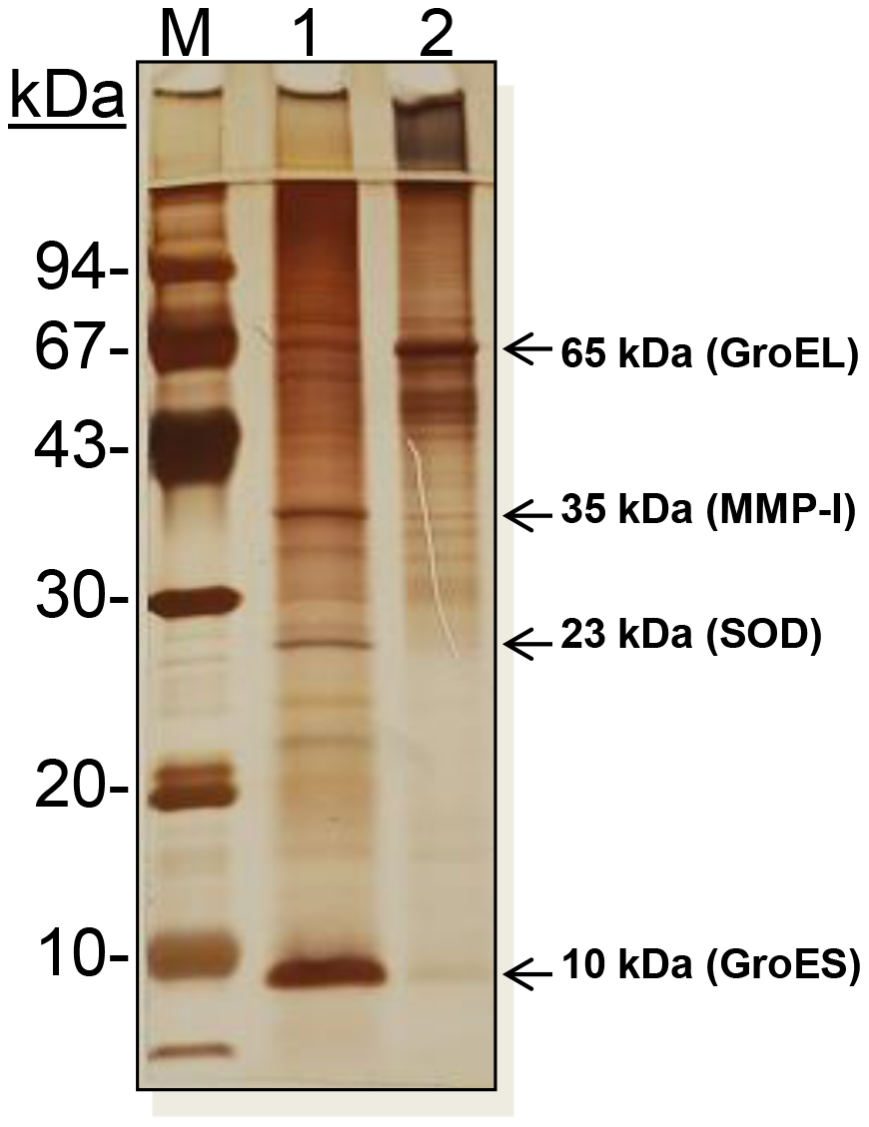
Protein profile of MLSA-LAM and MLCwA. Unstained molecular weight markers (lane M), and 2 µg of pre-autoclaved leprosy skin test antigens MLSA-LAM (lane 1) and MLCwA (lane 2) were separated on a 15% reduced polyacrylamide gel and visualized by staining with silver nitrate. The depicted proteins were located by immunoblot with the corresponding monoclonal antibodies as described in the materials and methods. The SOD protein is a 23 kDa protein based on amino acid sequence, but resolves at 28 kDa under reduced gel electrophoresis conditions [50].

Assays to assess MLSA-LAM and MLCwA critical quality attributes of identity, purity, sterility, potency, and safety were performed [92]. Ten vials of each antigen dose (2.5, 1.0, and 0.1 µg/0.1 ml) planned for clinical studies were tested on all assays with two exceptions. Identity testing by gel electrophoresis and immunoblotting was performed on samples taken prior to autoclaving, which degrades proteins resulting in smearing of bands on gels and immunoblots. A representative silver stained gel of both antigen preparations is shown in **Figure 5**. Immunoblotting results showed that neither antigen preparation had detectable armadillo tissue or LAM present, both contained MMP-I, and only MLSA-LAM contained GroES and SOD, while only MLCwA contained GroEL proteins. Purity testing for adventitious agents was performed on tissue homogenates and concentrated final product (10.0 µg and 5.0 µg/0.1 ml); both were free of detectable human viral pathogens. The presence of collagenase, Triton X-114, and SDS were tested and found to be less than the lower limit of detection. Extracti gel D ligand was not tested, because if released, it would be removed by filtration prior to vialing. Calcium chloride, polyethylene glycol, and Dextran T-500 were not tested, because following multiple washes, the calculated residual concentration in the purified bacteria suspension had decreased by 46-fold and was found to be harmless as demonstrated in animal safety studies.

Antigen preparations were found to be sterile under aerobic and anaerobic conditions and potent when assessed for a DTH response in guinea pigs sensitized with *M. leprae* or infected with *M. tuberculosis*. Stability, although not a critical quality attribute was assessed during product development using a research batch and prior to and during clinical testing, resulting in 4 years of satisfactory results. All test results were used to complete the regulatory package. The Lot Release Summary and stability results for both MLSA-LAM and MLCwA can be found in **Table 2**.

**Table 2:** Lot release and stability summary: MLSA-LAM and MLCwA Batch No. 23, Lot No. 051297.

Quality Attribute	Test Method	Specification	Results	
			MLSA-LAM	MLCwA
Identity	Protein Concentration	Diluted to concentration	Pass	Pass
	Reduced Silver Stain Gel	Expected profile	Pass	Pass
	Immunoblots	Expected profile	Pass	Pass
Purity	Viruses: Culture for CPE, PCR	Not detected	Pass	Pass
	Endotoxin Concentration	≤0.5 EU/ml	Pass	Pass
	Collagenase	Not detected	Pass	Pass
	Residual SDS	≤0.001%	Pass	Pass
	Residual Triton X-114	Not detected	Pass	Pass
Potency	DTH in Guinea Pigs	Induration at 1 µg/0.1 ml	Pass	Pass
Sterility	21 CFR 610.12	No growth	Pass	Pass
Safety	21 CFR 610.11, guinea pigs & mice	All survive, no weight loss at 7 days, no AE	Pass	Pass
Stability	DTH in Guinea Pigs, Temp: 4°C and 20°C, Time: d90, d120, d360, y2 and y4	Induration at 1 µg/0.1 ml	Pass	Pass

### Investigational New Drug

In 1994, a draft Investigational New Drug (IND) [93] was formulated and specific questions related to IND enabling studies, manufacturing, and phase I clinical trial design was sent to our NIH, NIAID, DMID program officer at the time (the late Dr. Darryl Gwinn) and Regulatory Affairs Specialist (Ms. Carol Manning) for submission to the FDA Center for Biologics and Evaluation Research (CBER) for preliminary review and comment.

A FDA Response Letter with a comprehensive list of queries was received. The first topic of focus was the armadillo infected tissue and included questions on the following subject matters: 1) the origin, isolation, and characterization of the *M. leprae* strain; 2) creation, storage, maintenance, and viability testing of the master seed stock; 3) armadillo quarantine, test for human pathogens, and general health status; 4) potential human infectivity of indigenous armadillo microorganisms; 5) armadillo inoculation procedures and biosafety procedures for staff; and 6) test for viral adventitious agents. The second topic of concern centered on the manufacturing and characterization process, including questions on: 1) procedural flow charts; 2) potential or known human toxicities and quantitative tests for reagents used in the manufacturing process; 3) qualitative compositional analyses for each skin test antigen; 4) presence of cross-reactive antigens; 5) level of host contamination, endotoxin, and sterility; 6) i*n-vitro* and *in-vivo* potency assays conforming to intended clinical use in humans; 7) stability testing prior to clinical studies; and 8) preclinical testing of clinical lots for safety, activity, and skin test conversion in a dose ranging study. Further questions were raised regarding the clinical phase I study design: 1) clinical study details; 2) potential impact of anergy regarding leprosy and HIV patients; 3) consent form and Institutional Review Board for each study site; 4) Case Report Forms for data collection; 5) references supporting related antigens and clinical studies; and 6) distinguishing subjects that are infected or harboring live bacilli from those who are infected and cured. A reply to the FDA Response Letter was satisfactory and a Pre-IND Meeting followed to review details of the manufacturing and testing process.

Skin test antigens were manufactured in May, 1997. The IND chemistry, manufacturing, and control (CMC) section was then completed and our DMID Study Sponsor submitted the IND Application to CBER for review. In September, 1998, FDA allowed the clinical investigation of two new drugs, MLSA-LAM and MLCwA, to proceed each at 3 doses (2.5, 1.0, and 0.1 µg) initially in a phase I clinical trial with ten healthy subject residing in a non-endemic region for leprosy, and subsequently in a phase II clinical trial with healthy subjects, leprosy patients, leprosy patient contacts, and tuberculosis patients residing in an endemic region for leprosy.

## Discussion

A tool for the detection of pre-symptomatic leprosy is an urgent need [94], [95]. How to address the treatment of individuals with evidence of specific leprosy exposure is a matter of debate [10]; chemoprophylaxis is proving highly efficacious in the short term, as applied to household contacts [96]. Individuals positively identified as pre-symptomatic could be a tool in the identification and further management of the disease, particularly reduction of incidence, i.e. NCD. Serological and gene approaches had not proven satisfactory for the purpose of diagnosing leprosy [56]; although these and other test methods are continually being refined and evaluated, in particular: details of new *M.* leprae antibodies [97], [98], new approach in the application of *M. leprae specific* DNA polymerase chain reaction [99]–[101], and cell-mediated immune response assays primarily based on IFN-γ release [102], [103]. While tests for PGL-I IgM antibodies have found favor for certain applications, most are not suitable for epidemiological application [104]. However, the two new leprosy antigens described here, MLSA-LAM and MLCwA, showed promise in guinea pig DTH studies and IFN-γ release assays [53], [54], [105]. Skin testing is the only means for mass epidemiological screening. Antigens for this purpose were targeted for product development as new leprosy skin test antigens.

Notwithstanding significant challenges, the development and manufacturing of these two leprosy skin test antigens suitable for human application was successfully accomplished. Securing adequate funding, identifying a large team of experts, and establishing a product development plan were key achievements that benefited the entire development phase. Changing the focus and practices of the research staff from basic to applied research enabled production of the skin test antigens. The magnitude of effort necessary in meeting regulatory requirements, in particular, substantial documentation, compounded by limited staff, funding, and experience was demanding A special attribute of this undertaking was the oversight of NIH, NIAID, DMID sponsor who provided financial, technical, and regulatory assistance, and served as a conduit to the FDA for cGMP and IND related questions.

The positive impact of developing and manufacturing these two new leprosy skin test antigens in an academic setting was realized only after successful implementation. The effort produced knowledge, skill, and understanding of the product translational process at the academic institutional level. Students were a valuable asset and in return, gained a unique learning opportunity. Looking forward, this work provides a product development template for products of neglected tropical diseases. Academic institutions cannot carry the heavy load of full product development alone, but this prototype presents alternative opportunities to move viable product ideas from the bench to the clinic.

The outcome was two new leprosy skin test antigens, suitable for human application, produced in a setting inexperienced in the manufacture of products for human use. This was necessitated by our focus on one of the major neglected tropical diseases of our time, and one of little commercial value. A consequence of this effort was the establishment of a contract manufacturing organization, Biopharmaceutical Manufacturing in an Academic Research Center (BioMARC), at Colorado State University for developing and manufacturing biological products to test in early clinical studies.

## Supporting Information

Protocol S1 **Phase I clinical protocol.** The phase I clinical trial was conducted in a non-endemic region for leprosy. The final revised version of the protocol (version 2.0, dated February 25, 1998) is attached.
(PDF)

Protocol S2 **Phase II clinical protocol.** The phase II clinical trial was conducted in an endemic region for leprosy. The final revised version of the protocol (version 9.0, dated March 2, 2009) is attached.
(PDF)
