# Supplementary material for: The Challenge of Producing Skin Test Antigens with Minimal Resources Suitable for Human Application against a Neglected Tropical Disease; Leprosy
Source: PLoS Negl Trop Dis. 2014 May 29;8(5):e2791. doi: 10.1371/journal.pntd.0002791 (PMC4038479; doi:10.1371/journal.pntd.0002791)
Supplement: Protocol S1 — Phase I clinical protocol. The phase I clinical trial was conducted in a non-endemic region for leprosy. The final revised version of the protocol (version 2.0, dated February 25, 1998) is attached. (PDF) [file pntd.0002791.s001.pdf]

Phase I Study to Evaluate New Leprosy Skin Test Antigens: MLSA-LAM and MLCwA

Investigators:

Jane Austen Higgins, M.D.
Cheri L. Lazar, R.N.
Patrick J. Brennan, Ph.D.
Stephen M. TerLouw, B.S.

Site:

Hartshorn Health Services Center
Colorado State University
Fort Collins, CO 80523

Version 2.0
February 25, 1998

Phase I Study to Evaluate New Skin Test Antigens: MLSA-LAM and MLCwA

Precis

This is a small Phase I trial to evaluate the safety of the use as skin test antigens of the immunologically active proteins from the soluble/cytosol and insoluble cell wall of *Mycobacterium leprae*. The overall objective is to generate new leprosy skin-test antigens, equivalent to tuberculin-PPD in the tuberculosis context, to be used (i) for the early diagnosis of leprosy; and (ii) as epidemiological tools to measure the incidence of disease. Evaluating new leprosy skin test antigens may provide a better way to diagnose leprosy in its early stages of infection. With the early administration of drug therapy, infected individuals can then be cured of this disease before nerve damage occurs or nodules (lepromas) start to develop on the skin.

Accordingly, the initial and immediate objectives are focused: (i) to determine that the soluble proteins of *M. leprae*, devoid of approximately 80% of the cell's immunosuppressive lipid-carbohydrate (MLSA-LAM), and the cell-wall associated proteins (MLCwA) are safe for humans as skin test antigens; and (ii) to help determine the best concentrations of these products to be used as skin test antigens. The products to be tested (MLSA-LAM and MLCwA) may not provide the requisite specificity, but they are first generation antigens that require testing. From them will probably emerge antigens with the necessary specificity and sensitivity.

Five individuals will be selected for testing MLSA-LAM and five will receive MLCwA. Each will receive single 0.1 ml intracutaneous injections of three titrated doses (1, 10 and 25 µg/ml) of the skin-test antigen, one of mock antigen (i.e., physiological saline), and one of control antigen (the product now in use; Rees MLSA, 10 µg), divided equally between the subject's two arms. Test sites will be evaluated for areas of induration by the same methods used at the Hartshorn Health Services, Colorado State University, to determine the response to tuberculin/PPD, i.e., induration and erythema, using calipers or a ruler. Results will be recorded at 15 min, 48 h, 72 h, and 28 days post-injection. It is expected that all concentrations of the two leprosy skin test antigens will evoke similar negative responses. If so, all three concentrations will be used for subsequent studies. If some of the higher doses (e.g., 25 µg/ml) result in untoward erythema/necrosis, these doses will be dropped.

020

Background

The dominant development in leprosy (Hansen's Disease) over the past five years has been the dramatic decline in worldwide prevalence from a figure of 5.5 million cases in 1991 to 2.3 million in 1994 (1). The initial dramatic drop was attributed to the medical redefinition of a case of leprosy and to the same type of sociological developments that resulted in the elimination of leprosy in Europe in the 1800s. However, it is now clear that the aggressive implementation of multidrug therapy (MDT) and of case finding, combined with favorable sociological developments in endemic areas, are the major factors in the continuing dramatic decline. As yet, no predisposing relationships between HIV infection and leprosy have been observed, and relapse/drug resistance (with a cumulative failure/relapse rate of less than 1% over a 9-year follow-up period) is not a problem (1, 2).

In the absence of effective tests for early/sub-clinical leprosy, there is little concrete information on incidence and, hence, on the total disease; we do know that the number of new cases continues to rise, currently at a rate of approximately 650,000 per year (1,3). Development of tests for early diagnosis and charting of the full epidemiology of leprosy is now the greatest need. It is agreed by most of those contributing to the global leprosy elimination program that the most important contribution from current research endeavors will be "tools to identify sub-clinical infection of sufficient sensitivity and specificity . . . to facilitate epidemiological monitoring of the disease in the community" (S.K. Noordeen in Ref. 1). Serological and gene amplification approaches have not met the demanding requisites of such epidemiological tools in terms of specificity, sensitivity, and ease of operation (4). The remaining hope lies in the development of new skin test antigens on par with, if not better than, the tuberculin/PPD test in tuberculosis.

We have learned much by analyzing the historical progression of skin testing in leprosy patients. The earliest published information by Mitsuda in 1919 (5) involved skin testing of volunteers with an autoclaved suspension of macerated nodules from untreated leprosy patients. This antigen (Lepromin-H), when injected into patients with lepromatous leprosy, did not evoke any type of response, but, when injected in patients with tuberculoid leprosy (and in a certain percentage of indeterminate and borderline patients), produced, 30 days later, a nodule varying in size from 3 to 4 mm, termed the Mitsuda reaction. In 1940, Fernandez (6) described another response produced by this antigen, which appeared 48 h after injection in

021

those individuals with tuberculoid leprosy and in a certain number of normal people who had been in contact with persons with leprosy. The presence of a Fernandez reaction is considered indicative of some degree of cell mediated immunity (CMI) against *M. leprae*. A derivative of Lepromin-H, a chloroform ether-extracted suspension of *M. leprae*, labeled Dharmendra Lepromin, regularly produced only the 48 h reaction (7), the protocol for this leprosy skin test antigen is on file with the FDA (BB-IND-2399) (8). This second induction peak is unique and is considered to be a measure of response capability to *M. leprae* antigens. However, because about 90% of normal subjects in endemic areas produce a positive response, an induration of 3 mm or more, 21 days after intradermal injection of lepromin, the test is not considered useful for diagnosing leprosy.

As leprosy declined, and as MDT was widely implemented, the number of patients with fulminating leprosy declined and the availability of human lepra nodules was reduced dramatically. Hence it became important to find another source of antigen. In 1975, it was shown that armadillo-derived Lepromin-A showed equal or better delayed type hypersensitivity (DTH) and Mitsuda reactions than Lepromin-H in preliminary human clinical studies (9-11). These results led to the submission and subsequent FDA approval of an IND for Lepromin-A (BB-IND 2401) in 1981 via Dr. W. A. Krotoski and Dr. R.C. Hastings at the Gillis W. Long Hansen's Disease Center (GWLHDC), Carville/Louisiana State University, Baton Rouge, LA; Lepromin-A is now prepared there for domestic and international use (8). Lepromin-A has prognostic value solely for classifying disease type. By January 1993, under a contract with the WHO, approximately one million doses of Lepromin-A had been distributed to physicians and institutions in endemic areas. Its general safety and effectiveness are well established. However, two unfavorable results are apparent. First and foremost is the frequent appearance of a nodule and/or necrosis at the site of injection after 3-4 weeks, commonly referred to as the Mitsuda reaction. Second, the standard Lepromin-A by itself can act as a weak vaccine and immunologically disturb any population receiving this reagent (12).

It became apparent by 1984 that a skin test reagent capable of specifically diagnosing leprosy would have to come from fractionated *M. leprae*. To mimic the classical tuberculin PPD in which activity/specificity are related to low molecular weight, secretory proteins in the culture filtrate (13), efforts were directed to the soluble fraction of the leprosy bacilli, since the equivalent of culture filtrate proteins of *M. leprae* could

not be obtained. Two different laboratories formulated two different subcellular skin test antigens at about the same time: Convit's antigen, (also know as SPA, Soluble Protein Antigen or SA, Soluble Antigen) and Rees' Antigen, (also known as MLSA, *M. leprae* Soluble Antigen or Leprosin) (14). Convit's SPA is the soluble component prepared from "live" *M. leprae* purified from armadillo organs by the so-called "1/79 Draper protocol" (15), disrupted by French press, and centrifuged at 48,000 x g for 1 h. The supernatant (i.e., cytosolic and some membrane components) was filtered through a 0.45μ filter followed by isolation of low molecular weight components using an ultra-filter with a 30,000 Dalton cut-off. The soluble protein antigens (SPA preparation) was again filtered through a 0.45μ filter prior to dilution in borate buffer and bottling in vials. These were then autoclaved, cooled and stored at -20°C. Rees' antigen, or MLSA, on the other hand, consists of "pure" cytosolic fraction. Gamma-irradiated *M. leprae*-infected armadillo tissues are subjected to the so-called "3/77 Draper protocol" (16) to extract pure *M. leprae* followed by sonication to disrupt the bacilli, a 27,000 x g centrifugation to remove the cell wall, and a 30 min 105,000 x g centrifugation to remove the membranes. The soluble fraction is filtered through 0.8μ and 0.2μ filters prior to dilution in borate/Tween buffer and bottling. This reagent is stored at 4°C.

These efforts did represent an appreciable advancement in leprosy diagnosis. Extensive testing of human subjects with both antigens has been undertaken in Malaysia, Malawi, Venezuela and elsewhere (14, 17-20) with no adverse effects. Reactions observed in these studies ranged from "soft," meaning that the reaction merged almost imperceptibly with the surrounding skin, to "hard," meaning that the reaction was quite distinct in the margins (reactions to tuberculin are almost invariable hard in consistency). The reason for this soft reaction is unknown, but obviously makes the test extremely difficult to read consistently (17). However, results indicate that the Rees and Convit antigens are not useful in the identification of *M. leprae* infection or in the confirmation of leprosy diagnosis in a leprosy endemic population with a high prevalence of non-specific sensitivity (18). It should be mentioned that neither Convit's nor Rees' antigen exhibit sensitizing potential, both are very potent immunologically, and their use in the human vaccine trials in Venezuela, Malawi, and India (14, 19) demonstrates that they are certainly safe, and, in a limited sense, useful.

Work by Samuel *et al.* (20) in India, Uganda, Kenya, Nepal, and Bhutan documented that the Rees antigen reaction were positive in highly resistant forms of leprosy and negative in low-resistant lepromatous forms. Wide variations in response to both of these antigens are well documented by Gupte *et al.* (18). Reasons for these differences could be the use of different batches of antigens, prevalence of different levels of leprosy endemicity and non-specific desensitization, as well as the differences in populations studied in different geographical locations. In general, the results of these studies indicate that Convit's and Rees' antigen skin test responses are variable due to product or population variation, may not be sensitive enough to detect leprosy, and do not appear to be specific enough to confirm clinical diagnosis of leprosy. Thus, in these limited trials, both the Convit and Rees skin test antigens meet the ideal for potency while falling short in terms of sensitivity and specificity.

At this juncture, we wish to establish the safety of MLSA-LAM and MLCwA and then proceed to the questions of sensitivity and specificity by testing them directly in an appropriate population and by fractionating and testing the progeny.

Study Objectives

The overall objective is to generate a new leprosy skin test antigen, equivalent to PPD/tuberculosis, as a diagnostic epidemiological tool to measure the incidence of leprosy. The specific objectives are:

- To determine that *M. leprae* soluble antigens devoid of immunosuppressive lipid-carbohydrate moieties (MLSA-LAM) and *M. leprae* cell wall antigens (MLCwA) are safe to use in humans as a skin test antigen.

- To determine that the range of concentrations chosen for skin testing does not elicit a reactive response in a negative control group of human subjects.

Study Design

Ten healthy, leprosy-unexposed, PPD-negative people from the non-endemic area of Fort Collins, Colorado, will be studied. Five individuals will be randomly selected for testing MLSA-LAM and the other five will receive MLCwA.

024

The general approach to be followed in evaluating the drug involves the recruitment of volunteers, an explanation of the purposes of the study, the risks and benefits involved, and the signing of an informed consent form.

Before recruitment into the study, each volunteer will complete a questionnaire to determine health status and, if female, will be asked to provide a urine sample for pregnancy testing. Only healthy, non-pregnant individuals who are skin test negative to PPD/tuberculin will be enrolled in the study. Each individual will receive three injections of titrated doses (in concentrations of 1 µg of protein content/ml, 10 µg/ml, and 25 µg/ml in 0.1 ml volumes in sterile diluent; PBS/Tween 80) of one of two skin test antigens, one injection of control antigen (Rees' MLSA at 10 µg/ml), and one injection of mock antigen (diluent). The concentration ranges to be tested are based on previous skin testing in humans with Rees' antigen (optimum dosage is 1 µg, i.e., 10 µg/ml), as well as skin test results in guinea pigs.

Skin test reagents will be administered in 0.1 ml volumes at the above concentrations in normal sterile diluent. Three test sites will be on the flexor surface of the forearm about 2 inches, 3 inches, and 6 inches below the bend of the elbow. The other two test sites will be at 3 inches and 6 inches below the bend of the elbow on the other forearm. The skin of the forearm will first be cleansed with alcohol and allowed to dry. The test dose will be administered with a sterile, 1 ml syringe calibrated in tenths and fitted with a sterile, one-half inch, 26 or 27 gauge needle. The rubber stopper of the vial will be wiped with a sterile piece of cotton moistened with alcohol and allowed to dry. The needle will then be inserted gently through the rubber septum and the required amount of the test antigen drawn into the syringe. The point of the needle will be inserted into the most superficial layers of the skin with the needle bevel pointing upward. If the intracutaneous injection is performed properly, a definite white blob will rise at the needle point, about 10 mm (3/8 inch) in diameter. This will then disappear within minutes. No dressing is required.

Test sites will be evaluated for area of induration by palpation and measurement of induration and erythema, using a ruler or calipers, and results will be recorded at 15 min, 48 h, 72 h, and 28 days post-injection.

All concentrations of each antigen resulting in a reaction less than 10 mm in duration will be chosen to administer to the larger test group of volunteers in future studies.

Study Population

Ten healthy, tuberculin-negative volunteers from within the staff of the Mycobacteria Research Laboratories, Department of Microbiology, Colorado State University, Fort Collins, Colorado, will be selected for this study. The volunteers will be recruited by direct contact after placing notices in the Department of Microbiology at Colorado State University. There are approximately 40 faculty/staff/students in this population and all are routinely tested (PPD skin-testing or chest X-ray) by the staff at the Hartshorn Health Service Center in the context of the Department's tuberculosis research.

A. Inclusion Criteria

- Born in the United States (less chance of previous exposure to tuberculosis or leprosy)
- Healthy individuals who are free of any evidence of leprosy or tuberculosis by established clinical examinations
- Between the ages of 18 and 40 years old
- Females of child-bearing age (defined from the period of menarche to menopause, usually between the ages of 15 to 45), certified as not pregnant (as determined by a pregnancy test performed within 7 days prior to admission into the study)
- Agreement to participate in the study after verbal explanation by the physician and nurses and signing of an informed consent form.
- Weight greater than 100 lbs. (female) and 140 lbs. (male)
- No known hypersensitivities or allergies
- Negative tuberculin skin test (as determined by skin tests performed 3 weeks prior to study entry)

B. Exclusion Criteria

- Pregnant or lactating females
- Oral corticosteroid treatment
- Chronic illness
- Immunosuppressive condition
- Tuberculosis
- Leprosy
- Age <18 or >40

- Weight <100 lbs. (female) or <140 lbs. (male)
- Positive tuberculin skin test
- Extensive travel (2-3 trips/year) in leprosy/tuberculosis endemic regions

Compensation

Volunteers will receive $50 compensation if they complete the study, which includes a total of four visits to the Hartshorn Health Service Center for administration of research antigens or examination of antigen sites at specific times. If the volunteer withdraws before the study is completed, he/she will receive compensation of $12.50 only for those visits made on time and the remaining amount will be forfeited.

Investigational Drug

Human trials will be conducted only with the one large batch each of MLSA-LAM and MLCwA prepared in the Pilot plant facility at Colorado State University. The "drug substances" to be tested are the soluble proteins of *M. leprae* (i.e., MLSA, *M. leprae* soluble antigen with minimal amounts of the immunosuppressive lipoglycan mostly LAM, i.e., lipoarabinomannan) called **MLSA-LAM** and the cell wall-associated proteins of *M. leprae* called **MLCwA**. The active ingredients of these two intradermal skin test antigens are their protein antigens.

Antigen **MLSA-LAM** is derived from soluble *M. leprae* extract following sonication and centrifugation at 27,000 x g then 100,000 x g, leaving the cytosol (MLSA). This soluble material is then extracted with detergent (Triton X-114) to reduce the amount of carbohydrate and lipid constituents (lipoglycans). MLSA-LAM contains the soluble protein antigens of *M. leprae*; over 100 individual proteins are recognized on 2-dimensional gels, and less than 30 of these have been sequenced and the immunological responses studied in part (24). Foremost among them are the 70 kDa (DnaK), 65 kDa (GroEL), 45 kDa, 38 kDa, 35 kDa major membrane protein (MMP-I), 22 kDa superoxide dismutase (SOD), 18 kDa small heat shock protein (SmHSP), 18 kDa bacterioferritin (Bfr), 10 kDa (GroES), and the ribosomal proteins S7/S12 (21-26). The full spectrum of proteins in soluble and insoluble subcellular fractions of *M. leprae* have been recently demonstrated (27, 28).

Antigen **MLCwA** is the 27,000 x g pellet extracted three times at 56°C with 2% sodium dodecylsulfate (SDS) followed by removal of SDS by column chromatography. MLCwA is further treated with Triton X-114 to reduce the

027

amount of lipoglycans. MLCwA contains many of the same proteins as MLSA-LAM, particularly the 70 kDa and 65 kDa, the export/secretory proteins (notably the 30/31 kDa, multigene antigen 85 complex) (22) and also contains some larger uncharacterized proteins. The full spectrum of its proteins has been submitted for publication (28).

The structural formulae of only some of the protein-antigen constituents of these two formulations are known. In the two *M. leprae* extracts, there are dozens of proteins, but the structural formulae of only about 30 are known, notably the 70 kDa/DnaK homolog, the two 18 kDa products, the 22 kDa, the 10 kDa/GroES homolog, a number of small proteins, several S and L subunit ribosomal proteins, the cysA/Sulfate sulfurtransferase/rhodanese homolog and the Avi-3 homolog (21-28).

The pharmacological class of these two formulations is considered as intradermal skin test antigens. The dosage formulations of each skin test reagent is in concentrations of 1 µg/ml, 10 µg/ml, 25 µg/ml and the route of injection will be intradermally in 0.1 ml volumes.

Outcome Parameters

A. End-Point and Expected Results

The skin-test reactions to the various concentrations of MLSA-LAM and MLCwA will be compared (historically) to that evoked in tuberculosis-negative individuals by standard PPD and, directly, to that of the Rees soluble antigen (which in PPD-negative individuals gives a response similar to that of PPD). It is expected that all concentrations of the two leprosy skin-test antigens will evoke similar negative responses. If so, all three concentrations will be used for subsequent studies. If some of the higher doses (e.g., 25 µg/ml) result in untoward erythema/necrosis, these doses will be dropped.

B. Potential Risks

The anticipated risks are the same as those encountered with other intradermal antigens such as tuberculin. Areas of erythema and induration will occur in those responding to the antigens(s), but these are localized responses and, generally, do not cause any discomfort. For strongly reactive individuals, ulceration and necrosis may occur at the injection site. These areas will be examined daily to ensure that secondary infection does not occur and to confirm satisfactory healing. Individuals sensitive to Tween 80

may exhibit additional reactions and discomfort at the injection site. In these individuals, cold packs or topical steroids may be applied for symptomatic relief of associated pain and discomfort. Epinepherine will also be available to treat any acute, systemic hypersensitivity reactions that might occur. The study will be conducted under the supervision of Jane Austen Higgins, M.D., and performed by Dr. Higgins and Cheri L. Lazar, R.N., of the Hartshorn Health Service Center.

C. Potential Benefits

Evaluating new leprosy skin test antigens may provide a better way to diagnose leprosy in its early stages of infection. With the early administration of drug therapy, infected individuals can be cured of this disease before nerve damage occurs or lepromas (tumor-like growths) start to develop on the skin. Participation in this study may not directly benefit persons involved in the studies. However, the information gained about the early detection of individuals infected with leprosy should be beneficial to others with this affliction.

Data Analysis

Five individuals will be selected for testing MLSA-LAM and five will receive MLCwA. Subjects alternately will receive either MLSA-LAM or MLCwA. Each will receive single 0.1 ml intracutaneous injections of three titrated doses (1, 10 and 25 μg/ml) of the skin-test antigen, one of mock antigen (i.e., physiological saline), and one of control antigen (the product now in use; Rees MLSA, 10 μg), divided equally between the subject's two arms. Test sites will be evaluated for areas of induration by the same methods used at the Hartshorn Health Services Center, Colorado State University, to determine the response to tuberculin/PPD, i.e., evaluation and erythema, using calipers. Results will be recorded at 15 min, 48 h, 72 h, and 28 days post-injection. It is expected that all concentrations of the two leprosy skin test antigens will evoke similar negative responses. If so, all three concentrations will be used for subsequent studies. If some of the higher doses (e.g., 25 μg/ml) result in untoward erythema/necrosis, these doses will be dropped.

References

1. Anon. CTD (Control of Tropical Diseases) News. UNDP/WORLD BANK/WHO Special Programme for Research and Training in Tropical Diseases (TDR). TDR News. No. 45, p. 5, June 1994; also Anon. Weekly Epidemiological Record. World Health Organization (May 22, 1992) 67: 153-160, 1992; also Noordeen, S.K. Elimination of leprosy as a public health problem. State-of-the-art lecture, XIV Leprosy Congress, Orlando, FL (29 August - 4 September 1993). Int. J. Lepr. 62: 278-283, 1994

2. Noordeen, S.K. Eliminating leprosy as a public health problem; why the optimism is justified. Int. J. Lepr. 63: 559-566, 1995.

3. Smith, P.G. Revised estimates of global leprosy numbers. (Editorial.) Lepr. Rev. 63: 317-318, 1992; also Feenstra, P. Will there be a need for leprosy control services in the 21st century? (Paper presented by P. Feenstra at NSL press conference, Amsterdam, 27 January 1994). NSL (Netherlands Leprosy Relief) Assoc., Vol. 6. Pp. 1 and 8, May 1994. Lepr. Rev. 65: 297-299, 1994.

4. Brennan, P.J. Prospects for contributions from basic biological research to leprosy control. Int. J. Lepr. 63: 285-286, 1995.

5. Mitsuda, K. On the value of a skin reaction to a suspension of leprosy nodules. Jap. J. Derm. Urol. 19: 697-708, 1919.

6. Fernandez, J.M.M. The early reaction induced by Lepromin. Int. J. Lepr. 8: 1-14, 1940.

7. Dharmendra, D. The immunological skin tests in leprosy: the isolation of a protein antigen of *Mycobacterium leprae*. Ind. J. Med. Res. 30: 1-7, 1942.

8. Krotoski, W.A., T.F. Mroczkowski, T.H. Rea, B.C. Clements, R.E. Neimes, M.K. Kahkonen, C.K. Job, and R.C. Hastings. Lepromin skin testing in the classification of Hansen's disease in the United States. Am. J. Med. Sci. 305: 18-24, 1993; Krotoski, W.A., T.F. Mroczkowski, E.J. Shannon, L.E. Millikan, R.M. Sanchez, and R.C. Hastings. Lepromin responses in recipients of a candidate anti-leprosy bacterin vaccine (WHO-IMMLEP *Mycobacterium leprae* killed preparation in the USA). Int. J. Dermat. 32: 191-193, 1993.

9. Convit, J., M.E. Pinardi, J.L. Avila, and N. Atanzazu. Specificity of the 48-hour reaction to Mitsuda antigen. Bull. W.H.O. 52: 187-191, 1975.

10. Meyers, W.M., S. Kvernes, and C.H. Binford. Comparison of Reaction to human and armadillo Lepromins in leprosy. Int. J. Lepr. 43: 218-225, 1975.

11. Millar, J.W., C. Gannon, C.S. Chan. Comparison in leprosy patients of Fernandez and Mitsuda reactions using human and armadillo antigens. A double-blind study. Int. J. Lepr. 43: 226-233, 1975.

12. IMMLEP Steering Committee 1982. Testing of purified armadillo-derived *M. leprae* in man. Document finalized by the IMMLEP Steering Committee at its meeting, 10-12 June 1981. World Health Organization. TDR/IMMLEP/SC/TEST/81.1.

030

13. Seibert, F.B. The isolation and properties of the purified protein derivative of tuberculin. Am. Rev. Tuberc. Pulmon. Dis. (Supplement) 30: 713-720, 1934.

14. Vaccination trials against leprosy: a meeting of the epidemiology subgroup of Scientific Working Group on the Immunology of Leprosy, Geneva, 11-13 February, 1985; TDR/IMMLEP/EDP/85.3: p. 7-8.

15. Draper, P. Protocol 1/79: Purification of M. leprae. Annex 1 of the Enlarged Steering Committee for Research on the Immunology of Leprosy (IMMLEP) Meeting of 7-8 February 1979. Geneva: World Health Organization, 1979, p. 4.

16. WHO (1977) Report of the Third IMMLEP Scientific Working Group on Leprosy. Protocol 3/77. WHO Document TDR/SWG/IMMLEP (3)/77, p. 20.

17. Gupte, M.D., and D.S. Anantharaman. Use of soluble antigens in leprosy epidemiology. Lepr. Rev. 59: 329-335, 1988.

18. Gupte, M.D., D.S. Anantharaman, B. Nagaraju, S. Kannan, and R.S. Vallishayee. Experiences with *Mycobacterium leprae* soluble antigens in a leprosy endemic population. Lepr. Rev. 61: 132-144, 1988.

19. Convit, J., C. Sampson, M. Zuniga, P.G. Smith, J. Plata, J. Silva, J. Molina, M.E. Pinardi, B.R. Bloom, and A. Salgado. Immunoprophylactic trial with combined *Mycobacterium leprae*/BCG vaccine against leprosy: preliminary results. The Lancet 339: 446-450, 1992.

20. Samuel, N.M., J.L. Stanford, R.J.W. Rees, T. Fairbain, and R.B. Adign. Human vaccination studies in normal and contacts of leprosy patients. Int. J. Lepr. 56: 36-44, 1984.

21. Thole, J.E.R., B. Wielgs, J.E. Clark-Curtiss, T.H.M. Ottenhoff and T.F. Rinke de Wit. Immunological and functional characterization of *Mycobacterium leprae* protein antigens: and overview. Mol. Microbiol. 18: 791-800, 1995; Young, D.B., S.H.E. Kaufmann, P.W.M. Hermans, and J.E.R. Thole. Mycobacterial protein antigens: a compilation. Mol. Microbiol. 6: 133-145, 1992.

22. Pessolani, M.C.V., and P.J. Brennan. *Mycobacterium leprae* produces extracellular homologs of the antigen 85 complex. Infect. Immun. 60: 4452-4459, 1992.

23. Rivoire, B., M.C.V. Pessolani, C.M. Bozic, S.W. Hunter, S.A. Hefta, V. Mehra, and P.J. Brennan. Chemical definition, cloning, and expression of the major protein of the leprosy bacillus. Infect. Immun. 62: 2417-2425, 1994.

24. Mehra, V., B.R. Bloom, A.C. Bajardi, C.L. Grisso, P.A. Sieling, D. Alland, J. Convit, X. Fan, S.W. Hunter, P.J. Brennan, T.H. Rea, and R.L. Modlin. A major T cell antigen of *Mycobacterium leprae* is a 10-kD heat-shock cognate protein. J. Exp. Med. 175: 275-284, 1992.

25. Pessolani, M.C.V., D.R. Smith, B. Rivoire, J. McCormick, S.A. Hefta, S.T. Cole, and P.J. Brennan. Purification, characterization, gene sequence, and significance of a bacterioferritin from *Mycobacterium leprae*. J. Exp. Med. 180: 319-327, 1994.

26. Hunter, S.W., B. Rivoire, V. Mehra, B.R. Bloom, and P.J. Brennan. The major native protein of the leprosy bacillus. J. Bio. Chem. 265: 14065-14068, 1990.

27. Pessolani, M.C.V., and P.J. Brennan. Molecular definition and identification of new proteins of *Mycobacterium leprae*. Infect. Immun. 64: 5425-5427, 1996.

28. Marques, M.A.M., S. Chitale, P.J. Brennan, and M.C.V. Pessolani. Mapping and identification of the major cell wall-associated proteins of *Mycobacterium leprae*. Infect. Immun. Submitted for publication.

032
