# Supplementary material for: The Challenge of Producing Skin Test Antigens with Minimal Resources Suitable for Human Application against a Neglected Tropical Disease; Leprosy
Source: PLoS Negl Trop Dis. 2014 May 29;8(5):e2791. doi: 10.1371/journal.pntd.0002791 (PMC4038479; doi:10.1371/journal.pntd.0002791)
Supplement: Protocol S2 — Phase II clinical protocol. The phase II clinical trial was conducted in an endemic region for leprosy. The final revised version of the protocol (version 9.0, dated March 2, 2009) is attached. (PDF) [file pntd.0002791.s002.pdf]

Two New Leprosy Skin Test Antigens: MLSA-LAM and MLCwA Phase II Study in a Leprosy-Endemic Region

DMID Protocol Number: 00-002

Sponsored by:
National Institutes of Health, National Institute of Allergy and Infectious Diseases,
Division of Microbiology and Infectious Diseases

DMID Funding Mechanism: NIH, NIAID, DMID Contract N01 AI-25469

Pharmaceutical Support Provided by: N/A

Principal Investigators: *Min Bahadur Thapa, M.B.B.S. (Nepal).
+Patrick J. Brennan, Ph.D.

* Anandaban Hospital, Kathmandu, Nepal
+ Department of Microbiology, Immunology & Pathology,
Colorado State University, Fort Collins, Colorado, USA

DMID Clinical Project Manager: Robin M. Mason, M.S.

DMID Medical Monitor: Jonathon Rose, MD, MPH, DABPM

DMID Medical Officer Mamodikoe Makhene, M.D.

Version Number: 9.0
March 2, 2009

Statement of Compliance

This study will be carried out in accordance with Good Clinical Practice (GCP) as required by the following:

- U.S. Code of Federal Regulations dealing with clinical studies (45 CFR 46 and 21 CFR including parts 50 and 56 concerning informed consent and Institutional Review Board [IRB] regulations).
- Colorado State University IRB
- Nepal Health Research Council (NHRC)
- Completion of Human Subjects Protection Training

http://grants.nih.gov/grants/guide/notice-files/NOT-OD-01-061.html

http://cme.cancer.gov/c01/

SIGNATURE PAGE

The signatures below constitute the approval of this protocol and the attachments, and provide the necessary assurances that this trial will be conducted according to all stipulations of the protocol, including all statements regarding confidentiality and according to local legal and regulatory requirements and to the principles outlined in applicable U.S. federal regulations and ICH guidelines.

Sponsor – NIH, NIAID, DMID, Bethesda, Maryland, USA

Signed: ______________________________ Date: ______________

Name: Robin M. Mason, M.S.
Title: Clinical Trials Specialist, Respiratory Diseases Branch, DMID

Principal Investigator - Anandaban Hospital, Kathmandu, Nepal

Signed: ______________________________ Date: ______________

Name: Min Bahadur Thapa, M.B.B.S. (Nepal)
Title: Medical Officer

Principal Investigator - Department of Microbiology, Immunology & Pathology, Colorado State University, Fort Collins, Colorado, USA

Signed: ______________________________ Date: ______________

Name: Patrick J. Brennan, Ph.D.
Title: University Distinguished Professor

Table of Contents

page
Statement of Compliance i
Signature Page ii
List of Abbreviations vii
Protocol Summary 1

1 Key Roles 7
2 Background Information and Scientific Rationale 9
2.1 Background Information 9
2.1.1 Leprosy Today 9
2.1.2 Leprosy Skin Test Antigens, 1919-Today 10
2.2 Rationale 13
2.3 Potential Risks and Benefits 14
2.3.1 Risks 14
2.3.1.1 Skin Testing Risks 14
2.3.1.2 Blood Sampling Risks 15
2.3.2 Known Potential Benefits 15
3 Objectives 16
3.1 Primary Objectives 16
3.2 Secondary Objectives 16
3.3 Intervention and Doses being Evaluated 17
3.4 Method of Assessing Study Outcome 17
4 Study Design 18
4.1 Rationale for Design Features 18
4.2 Description of Study Groups 19
4.3 Approximate Time to Complete Study Enrollment 19
4.4 Sequence and Expected Duration of Subject Participation 19
4.5 Methods for Collecting Data for Assessment of Study Objectives 20
4.6 Covariates 21
4.7 Interim Analysis Plans 22
4.8 Safety Oversight, per DMID Guidelines; SMC 22
5 Study Population 24
5.1 Selection of the Study Population 24
5.1.1 Percentages of Women, Minorities and Children 24
5.1.2 Losses to Follow-Up 24
5.1.3 Subject Recruitment and Retention 25
5.1.4 Screening Subjects 25
5.1.5 Enrollment 27
5.1.6 Randomization 27
5.2 Subject Inclusion Criteria 27

Table of Contents - *continued*

page
5.3 Subject Exclusion Criteria 28
6 Randomization/Enrollment/Blinding Procedures 30
7 Study Procedures/Evaluations....31
7.1 Clinical Evaluations 31
7.1.1 Medical History 31
7.1.2 Medication(s) History 32
7.1.3 Physical Examination....33
7.1.4 Counseling....33
7.1.5 Reactogenicity Assessments 33
7.1.6 Review of Diary Cards 34
7.2 Concomitant Medications/Treatments 35
7.2.1 Permitted Medications/Treatments 35
7.2.2 Prohibited Medications/Treatments....35
7.3 Laboratory Evaluations....35
7.3.1 Clinical Laboratory Evaluations....35
7.3.1.1 Pregnancy test 35
7.3.2 Special Assays or Procedures 35
7.3.2.1 Laboratory Assays 35
7.3.3 Specimen Preparation, Handling, and Shipping....36
7.3.3.1 Specimen Preparation, Handling, and Storage 36
8 Study Visit Schedule....37
8.1 Screening Visit 37
8.2 Enrollment/Baseline Visit....38
8.2.1 Enrollment 38
8.2.2 Administration of Study Product....40
8.3 Follow-up Visits 42
8.3.1 Initial Follow-up....42
8.3.2 Subsequent Follow-up 43
8.4 Final Study Visit 46
8.5 Early Termination Visit 46
9 Study Intervention/Investigational Product 47
9.1 Study Product Acquisition 47
9.1.1 Formulation, Packaging and Labeling....47
9.1.2 Control Products: Saline and Tuberculin/PPD® 48
9.2 Product Storage and Stability....48
9.3 Training of Staff, Preparation, Dosage and Administration of Investigational Product....49
9.3.1 Training of Test Administration Staff....49
9.3.2 Preparation of Investigational Product 50

Table of Contents - *continued*

page

9.3.3 Dosage of Investigational Product 50
9.4 Accountability Procedures for the Investigational Product(s) 51
10 Assessment of Scientific Objectives (e.g., Safety or Sensitivity/specificity) 52
10.1 Methods and Timing for Assessing, Recording, and Analyzing Appropriate Outcome Measures 52
10.2 Modification and Discontinuation of Study Intervention/Investigational Product for a Participant 54
10.2.1 Dose/Schedule Modifications for a Subject 54
10.2.2 Criteria for Discontinuation of Study Intervention/Product for Withdrawal of a Subject (or a Cohort) 55
11 Assessment of Safety 56
11.1 Specification of Safety Parameters 56
11.2 Methods and Timing for Assessing, Recording, and Analyzing Safety Parameters 56
11.2.1 Adverse Events, Reactogenicity and Serious Adverse Events 56
11.2.1.1 Definitions 56
11.2.2 Management of Possible Adverse Events 58
11.2.3 Management of Possible Serious Adverse Events 58
11.3 Reporting Procedures 58
11.3.1 Adverse Events 58
11.3.2 Serious Adverse Events 58
11.3.3 Reporting of Pregnancy 61
11.4 Halting Rules 61
12 Clinical Monitoring Structure 62
13 Statistical Considerations 63
13.1 Overview and Study Objectives 63
13.1.1 Scientific Rationale 63
13.1.2 Objectives 63
13.2 Study Population 64
13.3 Study Design 66
13.4 Study Outcome Measures 67
13.5 Study Hypotheses 68
13.6 Sample Size Considerations 68
13.6.1 Safety 68
13.6.2 Leprosy Detection/Diagnosis 70
13.7 Participant Enrollment and Follow-Up 72
13.8 Planned Interim Analyses 72
13.8.1 Rules for Handling any SAE: 73
13.8.2 Rules for Handling Multiple AEs: 74

Table of Contents - *continued*

page

13.8.3 Safety Outcomes 74
13.8.4 Statistical Rules to Halt Enrollment 75
13.9 Analysis Plan 75
13.9.1 Study Enrollment and Compliance 75
13.9.2 Safety 75
13.9.3 Diagnostic Sensitivity/Specificity 76
13.9.4 Assay for Immune Responses 76
14 Access to Source Data/Documents 77
15 Quality Control and Quality Assurance 78
16 Ethics/Protection of Human Subjects 79
16.1 Declaration of Helsinki 79
16.2 Institutional Review Board 79
16.3 Informed Consent Process 79
16.4 Exclusion of Women, Minorities, and Children (Special Populations) 81
16.5 Subject Confidentiality 81
16.6 Study Discontinuation 82
17 Data Handling and Record Keeping 83
17.1 Data Management Responsibilities 83
17.2 Data Capture Methods 83
17.3 Types of Data 84
17.4 Timing/Reports 84
17.5 Provisions for Blinding 84
17.6 Study Records Retention 85
17.7 Protocol Deviations 85
18 Publication Policy 86
19 Literature References 87

APPENDICES
A. Schedule of Procedures/Evaluations
B. IFN-gamma Responses of Sensitized T-cells Exposed to Different Leprosy Skin Test Antigens
C. Volunteer Breakdown and Estimated Time for Completion of Testing

List of Abbreviations

AE	Adverse Event
AH	Anandaban Hospital
AUC	Area under the ROC Curve
BL	Borderline Lepromatous Leprosy
BP	Blood Pressure
BT	Borderline Tuberculoid Leprosy
CD	Compact Disk
CFR	Code of Federal Regulations
CMI	Cell-Mediated Immunity
CRF	Case Report Form
DCC	Data Coordinating Center
DMID	Division of Microbiology and Infectious Diseases
DRF	Data Resolution Form
DTH	Delayed Type Hypersensitivity
eCRF	Electronic Case Report Form
ENL	Erythema Nodosum Leprosum
ER	Emergency Room
FDA	Food and Drug Administration
FPR	False Positive Rate
FWA	Federal-Wide Assurance
GCP	Good Clinical Practice
GEE	Generalized Estimating Equations
HRC	Human Research Committee
IB	Investigator’s Brochure
ICF	Informed Consent Form
ICH	International Conference on Harmonisation
IEC	Independent or Institutional Ethics Committee
IMB	Internal Monitoring Board
IND	Investigational New Drug
INF-γ	Interferon-gamma
IRB	Institutional Review Board
ISM	Independent Safety Monitor
LAM	Lipoarabinomannan
LL	Lepromatous Leprosy
LP	Lepromatous Leprosy Patient
MDT	Multidrug Treatment
MLCwA	*M. leprae* Cell Wall Antigen
MLSA	*M. leprae* Soluble Antigen
MLSA-LAM	*M. leprae* Soluble Antigen minus LAM and other Lipoglycans
MPA	Multiple Project Assurance
MO	Medical Officer
MOO	Manual of Operations
MRL	Mycobacteria Research Laboratory
N	Number (typically refers to subjects)
NCR	No Carbon Required
NHRC	Nepal Health Research Council

List of Abbreviations - *continued*

NIAID	National Institute of Allergy and Infectious Diseases
NIH	National Institutes of Health
ORA	Office of Regulatory Affairs
PPD	Purified Protein Derivative
PI	Principal Investigator
PO	Project Officer
Prn	Pro re nata (as needed)
QA	Quality Assurance
QC	Quality Control
OHRP	Office of Human Research Protection
ROC	Receiver Operating Characteristic
SAE	Serious Adverse Event
SC	Study Coordinator
SMC	Safety Monitoring Committee
SPA	Single Project Assurance
SPA	Soluble Protein Antigen
SA	Soluble Antigen
SOP	Standard Operating Procedure
TB	Tuberculosis
TD	Tuberculoid Leprosy Patient
TLMI	The Leprosy Mission International
TPR	True Positive Rate
TT	Tuberculoid Leprosy
WHO	World Health Organization

Protocol Summary

Title:
Two New Leprosy Skin Test Antigens: MLSA-LAM and MLCwA; Phase II Study in a Leprosy-Endemic Region

Phase:
Phase II Clinical Study

Population:
This study will be conducted in Kathmandu, Nepal. It will be divided into three stages: A, B, and C (Stage C will be subdivided into two parts; Stage C-1 and Stage C-1b). Study participants will total 260, with the following health status:

Stage A: 10 healthy non-exposed participants

Stage B: 90 healthy non-exposed participants

Stage C-1: 80 total participants
- 20 borderline lepromatous/lepromatous (BL/LL) leprosy patients
- 20 borderline tuberculoid/tuberculoid (BT/TT) leprosy patients
- 20 healthy contacts of BL/LL leprosy patients*
- 20 TB patients

Stage C-1b: 80 total participants
- 20 borderline lepromatous/lepromatous (BL/LL) leprosy patients
- 20 borderline tuberculoid/tuberculoid (BT/TT) leprosy patients
- 20 healthy contacts of BL/LL leprosy patients*
- 20 TB patients

Participants will be either male or female; however, not less than one-third will be recruited from either gender, overall. Each will be between the ages of 18 and 60 years old, with a weight greater than 30 Kg (66 lbs) for females, and 38 Kg (83 lbs) for males. All will be Nepali residents, including expatriates from India. Participants must not be concurrently enrolled in a separate clinical trial or have participated in prior stages of this clinical trial. All subjects screened for the study and found to have leprosy or tuberculosis will be treated or referred for treatment as per standard hospital procedure.

* A leprosy contact is defined as a co-inhabitant of a leprosy patient for a duration of at least six months, and within 6 months prior to study enrollment, or a person who has been professionally

exposed to leprosy for a duration of at least 5 years, and within 6 months prior to enrollment. (Enrollment of contacts of leprosy patients for Stages C-1 and C1b will include only co-inhabitants of BL/LL leprosy patients.)

Number of Sites:
Three campuses have been identified in Kathmandu, Nepal for participant recruitment for stages A and B, and four additional campuses have been identified in Kathmandu or Lalgadh for stage C (see Appendix C, Volunteer Breakdown and Estimated Timeline for Completion of Testing, and Section 5.1, “Selection of the Study Population”). Additional campuses/hospitals may also be selected to participate.

Study Duration:
Individual volunteers’ participation in each stage of the study will last for 7 days (± 1 day). If induration greater than 10 mm is observed at any site during the first 7 days post injection, an additional study visit will occur at 28 days. Total time involvement for each participant for all visits is approximately 5.0 hours.

Description of Agent or Intervention:
The drug substances to be tested are the soluble proteins of *M. leprae* [MLSA, *M. leprae* soluble antigen with minimal amounts of immunosuppressive lipoglycans, which are mostly lipoarabinomannan (LAM)], called MLSA-LAM, and the cell wall-associated proteins of *M. leprae,* called MLCwA. The active ingredients of these two intradermal skin test antigens are protein antigens of *M. leprae*.

Objectives:
The overall objective is to evaluate two new leprosy skin test antigens as diagnostic-epidemiological tools designed to measure the incidence of leprosy infection in a leprosy endemic area.

Primary:

- To evaluate the safety of these two new leprosy skin test antigens
- To estimate the specificity and sensitivity of these skin test antigens in detecting *M. leprae* infection by:
  - Selecting a dosage of the MLSA-LAM and MLCwA antigens that causes minimal induration in healthy non-exposed subjects
  - Selecting a size of induration that will serve as a definition of a positive skin test reaction for MLSA-LAM and MLCwA in leprosy patients
  - Comparing the proportion of positive skin test reactors in healthy subjects to the proportion in BT/TT and BL/LL leprosy patients, contacts of leprosy patients, and tuberculosis patients

Secondary:

- To compare the mean size of induration in response to each test antigen in healthy subjects versus BT/TT and BL/LL leprosy patients, contacts of leprosy patients, and tuberculosis patients as a measure of specificity and sensitivity
- To compare the specificity and sensitivity of the two new antigens with tuberculin/PPD (purified protein derivative), in patients with clinical leprosy, contacts of leprosy patients, and healthy, unexposed subjects (non-patient contacts)
- To quantify the release of interferon-gamma (IFN-γ) from lymphocytes in whole blood from leprosy patients, leprosy patient contacts, TB patients, and healthy non-exposed subjects, following *in vitro* stimulation with leprosy skin test antigens and PPD, using the QuantiFERON-CMI kit (Cellestis Limited, Valentia, California). Results will be compared to the magnitude of the skin test response.
- To determine if antibodies against a *M. leprae* specific antigen, Phenolic Glycolipid – I (PGL-I) are present in serum from leprosy patients, leprosy patient contacts, TB patients, and healthy non-exposed subjects, using a lateral flow immunodiffusion rapid test kit provided by Dr. Sang Nae Cho, Seoul, South Korea. Results will be compared to the magnitude of the skin test response.

Schematic of Study Design:

Stage A: Safety Study in Non-Contacts (Ramping)

Stage	Group	Sample/Size	Antigens	Dose	Intervention*
A	1	5<br>Non-Contacts	MLSA-LAM<br>MLSA-LAM<br>PPD<br>Saline	1.0 µg<br>0.1 µg<br>5 TU<br>N/A	Day –10 to –1: Recruitment<br>Day 0: Consent, Physical Exam, Pregnancy Test, History, **Administer Antigens** (~15 minute)<br>Day 2 (45-51 h): Reading<br>Day 3 (69-75 h): Reading<br>Day 25 to 31: Reading for positive reactors
	2	5<br>Non-Contacts	MLCwA<br>MLCwA<br>PPD<br>Saline	1.0 µg<br>0.1 µg<br>5 TU<br>N/A	

* Adverse Event assessment occurs at each reading time point.

Upon completion of Stage A, the Safety Monitoring Committee (SMC) will review the data and a report will be sent to DMID, NHRC, and CSU IRB. Stage B will proceed upon approval from each of these authorities.

Stage B: Safety Study in Non-Contacts (Full-Scale)

Stage	Group	Sample/Size	Antigens	Dose	Intervention*
B	1	45<br>Non-Contacts	MLSA-LAM<br>MLSA-LAM<br>PPD<br>Saline	1.0 µg<br>0.1 µg<br>5 TU<br>N/A	Day –10 to –1: Recruitment<br>Day 0: Consent, Physical Exam, Pregnancy Test, History, **Administer Antigens** (~15 minute)<br>Day 3 (69-75 h): Reading<br>Day 6 to 8: Reading<br>Day 25 to 31: Reading for positive reactors
	2	45<br>Non-Contacts	MLCwA<br>MLCwA<br>PPD<br>Saline	1.0 µg<br>0.1 µg<br>5 TU<br>N/A	

* Adverse Event assessment occurs at each reading time point.

Upon completion of Stage B, the SMC will review the data and a report will be sent to DMID, NHRC, and CSU IRB. Stage C will proceed upon approval from each of these authorities. Size and frequency of reactions will be a consideration in determining the use of one or both antigens as well as antigen dosages, in Stage C-1.

Stage C-1: Safety Study in Target Populations

Stage	Group	Sample/Size	Antigens	Dose	Intervention
C-1	1	20<br>Contacts of BL/LL leprosy patients<br>(co inhabitants only)	MLSA-LAM<br>MLCwA<br>PPD	1.0 µg<br>1.0 µg<br>2 TU	Day –10 to –1: Recruitment<br>Day 0: Consent, Physical Exam, Pregnancy Test, History, Blood Sampling, **Administer Antigens** (~15 minute)<br>Day 2 to 4: Reading<br>Day 6 to 8: Reading<br>Day 25 to 31: Reading for positive reactors
	2	20<br>BL/LL leprosy patients			
	3	20<br>BT/TT leprosy patients			
	4	20<br>TB patients			

In order to further assess the safety of skin test antigens in individuals who are more likely to present with a robust skin test response to the antigens, a small sample of 20 individuals from each group of the eventual target populations (healthy contacts of BL/LL leprosy patients, BL/LL leprosy patients, BT/TT leprosy patients, and TB patients) will receive the high dose (1.0µg) of each test antigen, and a 2TU dose of tuberculin/PPD. (Based on findings from preliminary testing of participants enrolled in Stage C-1, Tuberculin/PPD dose has been decreased from 5TU to 2TU for the remainder of Stage C-1 and for Stage C-1b.) Data will be reviewed by the SMC, and a report will be sent to DMID, NHRC, and CSU IRB.

Stage C-1b: Safety Study in Target Populations (Low dose)

Stage	Group	Sample/Size	Antigen	Dose	Intervention
C-1b	1	20<br>Contacts of BL/LL leprosy patients<br>(co inhabitants only)	MLSA-LAM<br>MLCwA<br>PPD	0.1 µg<br>0.1 µg<br>2 TU	Day –10 to –1: Recruitment<br>Day 0: Consent, Physical Exam, Pregnancy Test, History, Blood Sampling, **Administer Antigens** (Readings will be done ~15 and ~30 minutes after antigen administration)<br>Day 2 to 4: Reading<br>Day 6 to 8: Reading<br>Day 25 to 31: Reading for positive reactors
	2	20<br>BL/LL leprosy patients			
	3	20<br>BT/TT leprosy patients			
	4	20<br>TB patients			

Stage C-1b is a continuation of Stage C-1 to assess the reactivity of the low dose of each investigative antigen. There are indications from the blinded results arising from Stage C-1 that at least some individuals within the TB group react to more than one antigen (presumably the PPD and one of the leprosy antigens). Thus the inference is that the antigen concentration in some of the leprosy antigens is sufficiently high as to be cross reactive for TB and leprosy patients. For that reason Stage C-1b was designed to test the reactivity of the leprosy skin test antigens as the lower dose across the spectrum of patients. A small sample of 20 individuals from each group of the target populations (healthy contacts of BL/LL leprosy patients, BL/LL leprosy patients, BT/TT leprosy patients, and TB patients) will receive the low dose (0.1µg) of each test antigen, and a 2TU dose of tuberculin/PPD. Data will be reviewed by the SMC, and a report will be sent to DMID, NHRC, and CSU IRB.

1 KEY ROLES

For questions regarding this protocol, please contact Robin Mason at DMID via telephone at (301) 451-3746 or via email at [rmason@niaid.nih.gov].

Individuals:

Sponsor:
Christine F. Sizemore, Ph.D.
NIH/NIAID/DMID
Chief for Tuberculosis, Leprosy,
and Other Mycobacterial Diseases
Respiratory Diseases Branch, DMID
6610 Rockledge Dr., Room 6033
Bethesda, MD 20892
Tel: (301) 435-2857
Fax: (301) 496-8030
E-mail: csizemore@niaid.nih.gov

Sponsor's Clinical Project Manager:
Robin Mason, M.S.
NIH/NIAID/DMID
Clinical Trials Specialist
Respiratory Diseases Branch, DMID
6610 Rockledge Dr., Room 5036
Bethesda, MD 20892
Tel: (301) 451-3746
Fax: (301) 496-8030
E-mail: rmason@niaid.nih.gov

Sponsor's Medical Monitor for the Trial:
Jonathon Rose, MD, MPH, DABPM
Medical Officer
Office of Clinical Research Affairs
Division of Microbiology and Infectious Diseases
National Institute of Allergy and Infectious Diseases
National Institutes of Health
6610 Rockledge Drive, Room 6057, MSC 6603
Bethesda, Maryland 20892-6603
P: 301-443-7707
F: 301-480-0728
E-mail: rosejon@niaid.nih.gov

Sponsor's Medical Officer for the Trial:
Mamodikoe Makhene, M.D.
NIH/NIAID/DMID
Medical Officer
Respiratory Diseases Branch
6610 Rockledge Dr., Room 5043
Bethesda, MD 20892

Tel: (301) 402-8544
Fax: (301) 496-8030
E-mail: mmakhene@niaid.nih.gov

Investigator (on-site):
Min Bahadur Thapa, M.B.B.S. (Nepal)
Medical Officer PO Box 151
Deanna Hagge, Ph.D.
Head, Mycobacterial Research Labs
Tel: 977 (1) 429 0545
Mobile: 977 (1) 984 117 2400
977 (1) 974 115 6148
Fax: 977 (1) 429-0538
E-mail: drminthapa@gmail.com
Email: deannah@tlmnepal.org
Email: anandaban@tlmnepal.org

Qualified Physician (on-site) - ISM:
Bharat Kumar Yadav, M. D.
Patan Hospital
P.O. Box 252
Lagankhel, Lalitpur NEPAL
Tel: 011-977 (1) 552-2266 (Patan Hospital)
011-977 (1) 553-0003 (Patan Quarters)
977-1-557-2921 (home)
977-984-128-4476 (mobile)
Fax: 011-977 (1) 522-5559
E-mail: patan@hospital.wlink.com.np

Investigator (off-site):
Patrick J. Brennan, Ph.D.
University Distinguished Professor
Colorado State University
Department of Microbiology,
Immunology and Pathology
1682 Campus Delivery
Fort Collins, Colorado 80523
Tel: (970) 491-6700
Fax: (970) 491-1815
E-mail: Patrick.brennan@colostate.edu

2 BACKGROUND INFORMATION AND SCIENTIFIC RATIONALE

2.1 Background Information

2.1.1 Leprosy Today

The dominant development in leprosy, Hansen's Disease, over the past five years has been the dramatic decline in worldwide prevalence from a figure of about 2.4 million cases in 1991, to 1.2 million in 1994, to 0.8 million in 1998, and to less than 0.5 million currently (1). The initial dramatic drop was attributed to the medical redefinition of a case of leprosy, and to the same type of sociological developments that resulted in the elimination of leprosy in Europe in the 1800s. However, it is now clear that the aggressive implementation of multidrug therapy (MDT) and case finding, combined with favorable sociological developments in endemic areas, are the major factors in the continuing dramatic decline. As yet, no predisposing relationships between HIV infection and leprosy have been observed, and relapse/drug resistance (with a cumulative failure/relapse rate of less than 1% over a 9-year follow-up period) is not a serious problem (1, 2).

Given the absence of effective tests for early/sub-clinical leprosy, there is little concrete information on incidence and, hence, on the total numbers of leprosy patients. We do know that the number of new cases continues to rise, currently at a rate of over 600,000 per year (1, 3). This has been attributed by some to aggressive new case finding in the past few years. However, in the absence of true measures of incidence, true new cases cannot be distinguished from old ones. Development of tests for early diagnosis in order to chart the full epidemiology of leprosy is now among the greatest research needs. It is agreed by most of those contributing to the global leprosy elimination program that the most important contribution from current research endeavors will be "tools to identify sub-clinical infection of sufficient sensitivity and specificity . . . to facilitate epidemiological monitoring of the disease in the community" (S.K. Noordeen in Ref. 1). Serological and gene amplification approaches have not met the demanding requisites of such epidemiological tools in terms of specificity, sensitivity, and ease of operation (4). On the other hand, the recent recognition that the blood based QuantiFERON-TB Gold (Cellestis, Valentia, CA) kit can detect early immune responses to *M. tuberculosis* infection holds promise for similar specific cell-mediated immune tests for the presympotamic detection of leprosy. However, until leprosy specific antigens can be identified and evaluated in such clinical studies, the current hope for early detection of leprosy lies in the

development of new skin test antigens on a par with, if not better than, the tuberculin/PPD test used for tuberculosis.

2.1.2 Leprosy Skin Test Antigens, 1919-Today

We have learned much by analyzing the historical progression of skin testing in leprosy patients. The earliest published information, by Mitsuda in 1919 (5), involved skin testing of volunteers with an autoclaved suspension of macerated nodules from untreated leprosy patients. This antigen (Lepromin-H), when injected into patients with BL/LL leprosy, did not evoke any type of response, but, when injected in patients with BT/TT leprosy (and in a certain percentage of indeterminate and borderline patients), produced, 30 days later, a nodule varying in size from 3 to 4 mm, termed the Mitsuda reaction. In 1940, Fernandez (6) described another response produced by this antigen, which appeared 48 h after injection in those individuals with BT/TT leprosy, as well as in a certain number of normal people who had been in contact with persons with leprosy. The presence of a so-called Fernandez reaction is considered indicative of some degree of cell mediated immunity (CMI) against *M. leprae*. A derivative of Lepromin-H, a chloroform ether-extracted suspension of *M. leprae*, labeled Dharmendra Lepromin, regularly produced only the 48 h reaction (7). This second induction peak is unique and is considered to be a measure of response capability to *M. leprae* antigens. However, because about 90% of normal subjects in endemic areas produce a positive response, (defined as an induration of 3 mm or more, 21 days after intradermal injection of lepromin), the test is not considered useful for diagnosing leprosy.

As leprosy declined, and as MDT was widely implemented, the number of patients with fulminating leprosy declined and the availability of human lepra nodules was reduced dramatically. Hence, it became important to find another source of antigen. In 1975, it was shown that armadillo-derived Lepromin-A showed delayed type hypersensitivity (DTH) and Mitsuda reactions, equal to or better than Lepromin-H in preliminary human clinical studies (8-10). These results led to the submission and subsequent FDA approval of an Investigational New Drug (IND) for Lepromin-A in 1981 via Dr. W. A. Krotoski and Dr. R.C. Hastings at the Gillis W. Long Hansen's Disease Center (GWLHDC), Carville/Louisiana State University, Baton Rouge, LA. Until recently, Lepromin-A was prepared there for domestic and international use (11). Lepromin-A has clinical value solely for classifying disease type. By January 1993, under a contract with the WHO, approximately one million doses of Lepromin-A had been distributed to physicians and institutions in endemic areas. Its general safety and effectiveness are well established, however, two unfavorable results are apparent. First and foremost is the frequent appearance of a nodule and/or necrosis at the site of injection after 3-4 weeks. Second, the standard Lepromin-A by itself can act as a weak vaccine, not strong enough to protect from infection, yet immunologically compromising any population receiving this reagent (12). Thus, Lepromin-A is rarely used nowadays and is no longer produced at GWLHDC.

It became apparent by 1984 that a skin test reagent capable of specifically diagnosing early stages of leprosy would have to come from

fractionated *M. leprae*. To mimic the classical tuberculin/PPD, in which activity/specificity are related to low molecular weight, secretory proteins in the culture filtrate (13), efforts were directed to the soluble fraction of leprosy bacilli, since the equivalent of culture filtrate proteins of *M. leprae* could not be obtained. Two individual laboratories formulated two different subcellular skin test antigens at about the same time: Convit's antigen (also known as SPA, Soluble Protein Antigen or SA, Soluble Antigen), and Rees' antigen (also known as MLSA, *M. leprae* Soluble Antigen or Leprosin) (14). Convit's SPA is the soluble component prepared from "live" *M. leprae* purified from armadillo organs by the so-called "1/79 Draper protocol" (15), disrupted by French press, and centrifuged at 48,000 x g for 1 h. The supernatant (i.e., cytosolic and some membrane components) was filtered through a 0.45µ filter followed by isolation of low molecular weight components using an ultra-filter with a 30,000 Dalton cut-off. The soluble protein antigens (SPA preparation) were again filtered through a 0.45µ filter prior to dilution in borate buffer and bottling in vials. The vials were then autoclaved, cooled, and stored at –20ºC. Rees' antigen (or MLSA), by comparison, consists of "pure" cytosolic fraction. Gamma-irradiated *M. leprae*-infected armadillo tissues were subjected to the so-called "3/77 Draper protocol" (16) to extract pure *M. leprae,* followed by sonication to disrupt the bacilli, a 27,000 x g centrifugation to remove the cell wall, and a 30 min 105,000 x g centrifugation to remove the membranes. The soluble fraction was filtered through 0.8µ and 0.2µ filters prior to dilution in borate/Tween buffer and bottling. This reagent was stored at 4ºC.

These efforts did represent an appreciable advance in leprosy diagnosis. Extensive testing of human subjects with both antigens had been undertaken in Malaysia, Malawi, Venezuela, and elsewhere (14, 17-20), with no adverse effects. Reactions observed in these studies ranged from "soft," meaning that the reaction merged almost imperceptibly with the surrounding skin, to "hard," meaning that the reaction produced a distinct induration. The reason for this soft reaction is unknown, but obviously makes the test extremely difficult to read consistently (17). It should be mentioned that neither Convit's nor Rees' antigens exhibit sensitizing potential. Both are potent immunologically, and their use in the human vaccine trials in Venezuela, Malawi, and India (14, 18) demonstrates that they are certainly safe, and, in a limited sense, useful.

Work by Samuel *et al*. (19) in India, Uganda, Kenya, Nepal, and Bhutan documented that the Rees' antigen reactions were positive in BT/TT forms of leprosy and negative in BL/LL forms of leprosy (due to the noted immunological anergy at the LL pole of the disease). However, wide variations in response to both of these antigens were well documented by Gupte *et al*. (20). Reasons for these variations could be the use of different batches of antigens (very little quality control was conducted), prevalence of different levels of leprosy endemicity and non-specific desensitization, as well as the differences in populations studied in different geographical locations. In general, the results of these studies indicated that Convit's and Rees' antigen skin test responses are variable due to product or population variation, may not be sensitive enough to detect leprosy, and do not appear to be specific enough to confirm clinical diagnosis of leprosy. Thus, in these limited trials, both Convit's and Rees' skin test antigens meet the ideal for potency, while falling short in terms of sensitivity

and specificity. Thus, these skin test antigens fell out of favor and are no longer produced.

In 1992, given the pressing need for a diagnostic test to evaluate the incidence of leprosy, we started developing two new leprosy skin test antigens. In our attempts to instigate a new skin test initiative, we modified the Rees-type MLSA so as not to remove the large molecular weight, specific antigens, but to remove cross-reactive immunosuppressive components [such as LAM, LM (lipomannan), PIMs (phosphatidylinositolmannosides), and other lipids]. Secondly, we introduced cell wall proteins into the skin test format. In extensive *in vitro* immunological studies and guinea pig DTH testing with B.R. Bloom, V. Mehra, and R.L. Modlin (21, 22), we demonstrated, contrary to earlier reports (23), that the cell wall proteins of *M. leprae* (which were discarded in the Rees and Convit preparations) are powerful immunogens, and, accordingly, cell wall-derived preparations were included in the first phase of studies. The drug substances developed were: (i) the soluble proteins of *M. leprae* (i.e., MLSA, *M. leprae* soluble antigen), with minimal amounts of the immunosuppressive lipoglycan (mostly LAM, but also LM and the PIMs) called MLSA-LAM, and (ii) the cell wall-associated proteins of *M. leprae,* called MLCwA (*M. leprae* cell wall antigen). The active ingredients of these two intradermal skin test antigens are their protein antigens. A full description of these two antigens can be found in Section 9.1.1, "Formulation, Packaging and Labeling".

As a prelude to this study, Dr. Paul Roche tested MLSA-LAM, MLCwA, and Rees' Antigen in T-cell assays, using blood from various population groups at Anandaban Hospital (AH), Kathmandu, Nepal. The level of IFN-γ expressed was measured at 24 hours (See Appendix B). Results showed that there were significantly higher levels of IFN-γ produced in leprosy exposed as compared to unexposed Nepali subjects (including expatriates from India). From these data, we concluded that there should be distinct differences in skin test response of leprosy exposed compared to unexposed individuals.

These three antigens were also used in a Phase I study, which was performed on ten volunteers in Fort Collins, CO, during January and February of 1999. Five volunteers received three titrated, 100 µl doses of MLSA-LAM (0.1 µg protein, 1 µg, and 2.5 µg), one dose of a positive control (Rees antigen at 1 µg), and one 100 µl injection of mock antigen (saline). The other five volunteers received similar injections, except that the MLSA-LAM doses were replaced by MLCwA in similar doses. The injection sites were read at ~15 min, 48 h, 72 h, and 28 days.

All ten volunteers commented that, upon injection, all five sites stung as compared to a tuberculin/PPD injection. This symptom was attributed to the fact that tuberculin/PPD contains phenol, which acts as a local anesthetic, whereas MLSA-LAM and MLCwA are devoid of phenol. None of the volunteers mentioned any residual stinging after day 0 (the day of injection). Of the ten individuals tested, nine had no induration throughout the study, at any of the five sites. One volunteer showed indurations only at the 2.5 µg MLCwA and Rees antigen injection sites at 48 h and 72 h post injection. It should be noted that this volunteer could have been exposed to tuberculosis or leprosy antigens at work, and although she did not test positive to tuberculin/PPD, the fact that she was the only volunteer who did work on mycobacteria, is a consideration.

All volunteers had varying sizes of erythema and also experienced itching associated with the injection sites, especially in the case of the higher dosages of test antigens (2.5 µg MLSA-LAM and 2.5 µg MLCwA) and the control antigen (Rees antigen at 10 µg/ml). It was noted that in darker pigmented individuals, the erythema would probably go unrecognized, and therefore would not play a part in reading positive reactions. In most volunteers the itching disappeared after 72 h. Throughout the study some patients complained of various symptoms ranging from headaches to cold-like effects that were evaluated as not related to the study product.

2.2 Rationale

The eradication of leprosy requires the development of sensitive and specific tools to measure the prevalence of sub-clinical infections in the community so that persons with early or sub-clinical leprosy can be recognized and treated, to prevent transmission. Such tests should be safe, low cost, rapid, and easy to conduct. A skin test similar to tuberculin/PPD, but with specificity for leprosy, would allow the measurement of exposure to leprosy in community wide studies. The resources of leprosy control programs could then be focused on communities with high levels of exposure. In addition, the control programs would have a tool to measure the impact of their activities on the transmission of leprosy in the community.

Tools to assist with the eradication of leprosy are limited. Only traditional clinical diagnosis is widely used today. Due to concerns of sensitization when using Lepromin-A as a skin test reagent, and the fact that it is no longer being prepared in the United States, it cannot be used as a gold standard to compare the two new leprosy skin test antigens MLSA-LAM and MLCwA. In fact, the only means of estimating that these two antigens are measuring early infection is to confirm by clinical diagnosis. Assuming the infection is too early to detect by clinicians, subjects must be followed annually for a period of 3-5 years to monitor manifestations of leprosy disease, and thereby validate the diagnostic-epidemiological potential of these two new leprosy skin test antigens. A Phase III epidemiological study, including follow-up and sequential testing, may follow to substantiate the results of this clinical study.

Through an early initiative of the World Health Organization, Immunology of Leprosy (IMMLEP) Expert Committee, and through the guidance and resources of the National Institute of Allergy and Infectious Diseases, National Institutes of Health, Professor Patrick J. Brennan and colleagues of the Mycobacterial Research Laboratory, Department of Microbiology, Colorado State University, have been developing these two fractions of the leprosy bacillus, namely MLSA-LAM and MLCwA, as skin test antigens for human use. The Phase I studies in ten healthy volunteers at Colorado State University were conducted under a Food and Drug Administration (FDA) IND application. The Mycobacterial Research Laboratory and AH, Kathmandu, Nepal, have now combined to conduct the first trial of these new skin test antigens in Nepali (including expatriates from India) leprosy patients and healthy subjects. The aim of these studies is to establish the sensitivity and specificity of these antigens in leprosy patients as compared to TB patients. These studies are a critical step toward the development of these antigens as a tool for leprosy control.

The Phase II clinical plan comprises three separate Stages (A, B and C), to be completed successively. The first two Stages (A and B) will be performed to establish

the dosage of the two antigens inducing an optimal (less than 10mm) induration in healthy Nepali individuals (including expatriates from India). An induration less than 10mm is considered optimal for healthy non-exposed individuals based on interpretation of results from TB Mantoux testing. By TB Mantoux standards, an induration size smaller than 10mm is considered negative in moderate risk groups, that is, children under 4 years of age, foreign born individuals from high prevalence countries, and mycobacterial laboratory personnel, to name a few. Individuals recruited for Stage A and B are at moderate risk being from an endemic region for leprosy. A cut off less than 5mm is considered negative for TB exposure in individuals having close contact with a TB patient, those suspected of having TB disease, HIV infected individuals, or organ transplant individuals on immunosuppressive therapy, while those with no specific risk factors are considered negative with an induration less than 15mm. Until data is collected to determine baseline induration limits and induration cut-off limits for each study group, an optimal level of 10mm induration will be referred to herein.

Stages A and B will serve to re-confirm the U.S. data on the safety of these products, which will be done in two steps - first in 10 subjects (Stage A) and then in 90 subjects (Stage B). Having found an optimal safe dose for each antigen, both reagents will then be tested in 80 leprosy patients, 40 contacts of leprosy patients, and 40 tuberculosis patients (Stage C), and the results compared with the licensed TB reagent tuberculin/PPD. If the study results indicate that one or both leprosy skin test antigens show specificity and sensitivity in the order of 90% or above, future studies will be planned to measure the extent of leprosy infection in communities around the world, particularly the high prevalence countries of India, Nepal, Brazil, and some of the African countries.

Study Hypothesis: Both leprosy skin test antigens, MLSA-LAM and MLCwA at doses of 1.0µg and/or 0.1µg, will be safe and efficacious as diagnostic-epidemiological tools to detect and treat patients earlier and to measure the extent of leprosy infection in human subjects living in a leprosy endemic area.

2.3 Potential Risks and Benefits

2.3.1 Risks

2.3.1.1 Skin Testing Risks

The anticipated risks from administration of the skin test antigens are the same as those encountered with other intradermal antigens such as tuberculin/PPD. Areas of erythema and induration will occur in those responding to the antigens, but these are localized responses and generally, do not cause significant discomfort. For strongly reactive individuals, ulceration and necrosis may occur at the injection site. Injection sites which ulcerate will be examined daily to ensure that secondary infection does not occur and to confirm satisfactory healing. Individuals sensitive to Tween 80 may exhibit additional reactions and discomfort at the injection site for approximately 24 h after administration. In these individuals, cold packs or topical steroids may be applied for symptomatic relief of associated pain and discomfort. AH will provide

appropriate treatment for any adverse reactions in any participant directly caused by these procedures. Medical personnel and equipment will be available to those subjects who have adverse reactions caused by the procedures.

2.3.1.2 Blood Sampling Risks

Blood sampling by venipuncture may cause a bruise and/or bleeding at the needle site. Occasionally a person feels faint when blood is drawn. There is a rare chance that an infection may develop, but if this happens volunteers will be referred for appropriate follow up and treatment.

2.3.2 Known Potential Benefits

There are no known direct benefits to study participants, however, evaluating new leprosy skin test antigens may provide a better way to diagnose leprosy in its early stages of infection. With the early administration of drug therapy, infected individuals can be cured of this disease before nerve damage occurs. Participation in this study may not directly benefit the volunteers, however, the information gained about the early detection of individuals infected with leprosy should be beneficial to others with this affliction.

3 OBJECTIVES

The overall objective is to evaluate two new leprosy skin test antigens as diagnostic-epidemiological tools designed to measure the incidence of leprosy infection in a leprosy endemic area.

3.1 Primary Objectives

- To evaluate the safety of these two new leprosy skin test antigens
- To estimate the specificity and sensitivity of these skin test antigens in detecting *M. leprae* infection by:
    - Selecting a dosage of the MLSA-LAM and MLCwA antigens that causes minimal induration in healthy non-exposed subjects
    - Selecting a size of induration that will serve as a definition of a positive skin test reaction for MLSA-LAM and MLCwA in leprosy patients
    - Comparing the proportion of positive skin test reactors in healthy subjects to the proportion in BT/TT and BL/LL leprosy patients, contacts of leprosy patients, and tuberculosis patients

3.2 Secondary Objectives

- To compare the mean size of induration in response to each test antigen in healthy subjects versus BT/TT and BL/LL leprosy patients, contacts of leprosy patients, and tuberculosis patients as a measure of specificity and sensitivity
- To compare the specificity and sensitivity of the two new antigens with tuberculin/PPD (purified protein derivative), in patients with clinical leprosy, contacts of leprosy patients, and healthy, unexposed subjects (non-patient contacts)
- To quantify the release of IFN-γ from lymphocytes in whole blood from leprosy patients, leprosy patient contacts, TB patients, and healthy non-exposed subjects, following *in vitro* stimulation with leprosy skin test antigens and PPD, using the QuantiFERON-CMI kit (Cellestis Limited, Valentia, California). Results will be compared to the magnitude of the skin test response.
- To determine if antibodies against a *M. leprae* specific antigen, Phenolic Glycolipid – I (PGL-I) are present in serum from leprosy patients, leprosy patient contacts, TB patients, and healthy non-exposed subjects, using a lateral flow immunodiffusion rapid test kit provided by Dr. Sang Nae Cho,

Yonsei University, Seoul, South Korea. Results will be compared to the magnitude of the skin test response.

3.3 Intervention and Doses being Evaluated

Depending on the study stage, each individual will receive two 100 µl intradermal injections of titrated doses, or a single dose, of one or both of the two skin test antigens in concentrations of 1 µg protein content/ml and 10 µg protein/ml in the sterile diluent, one injection of 0.9% sodium chloride (sterile, approved for human use, Abbott Laboratories) in Stages A and B only, and one 100 µl injection of tuberculin/PPD Tubersol® 5TU; Aventis Pasteur Inc., Swiftwater, PA in Stages A and B and Tuberculin PPD RT 23 SSI, 2T.U./0.1 ml, solution for injection, Statens Serum Institute, Copenhagen, Denmark in Stages C-1 and C-1b. All injections will be in 0.1 ml volumes, resulting in 0.1µg and 1.0µg doses of test antigens, respectively. The concentration ranges to be tested are based on results of our Phase I study (see p. 12), previous skin testing in humans with Rees' antigen (optimum dosage is 1 µg) as well as our skin test results in guinea pigs.

3.4 Method of Assessing Study Outcome

The injection sites for all skin test antigens in the 260 individuals in this Phase II study (10 from Stage A, 90 from Stage B, and 160 from Stage C) will be evaluated by the same methods used to determine the response to tuberculin/PPD, i.e., evaluation of induration and erythema. The results from the healthy non-contacts studied in Stages A and B will be grouped. The results from this entire group will be compared to the results from the clinical leprosy patients, contacts of leprosy patients, and clinical tuberculosis patients in Stage C-1 and C-1b. Blood samples from each subject in Stage C will be collected to perform leprosy specific serology and blood based gamma interferon test.

4 STUDY DESIGN

This study will be a double-blind Phase II clinical trial conducted in three Stages (A, B, and C). Antigens are coded, and subjects are randomly assigned a template for placement of skin test antigens. Stage A will provide an initial indication of the safety of the two new test antigens in ten healthy members of this leprosy endemic population (five subjects per antigen at two dosages each). Stage B will expand this analysis by an additional 90 healthy subjects (45 subjects per antigen). If any of the subjects in Stage A or B show ulcerations at the 1.0µg dose of MLSA-LAM or MLCwA test sites, then only the 0.1µg dose will be used for Stage C. The final stage, Stage C, is divided into two parts. The first part, Stage C-1, will assess the safety of both antigens at the high dose (1.0µg) in populations at a higher risk of developing ulcerations at the skin test site. A total of eighty subjects will be recruited: 20 household contacts of BL/LL leprosy patients, 20 BL/LL leprosy patients, 20 BT/TT leprosy patients, and 20 TB patients The second part, Stage C-1b, is a continuation of Stage C-1 with the same number of subjects recruited from the same groups to assess the reactivity of both antigens at the low dose (0.1 µg). Safety data will be collected following each stage and reviewed by the SMC. A report will be forwarded to DMID, NHRC, and CSU IRB for review and approval.

This study will define a positive skin test reaction for MLSA-LAM and MLCwA and this definition will be used in estimating sensitivity and specificity for each skin test antigen and dosage. The skin test reactions (as compared to reactions from tuberculin/PPD, and Phase I studies of MLSA-LAM and MLCwA) will vary among the groups participating in this study. It is expected that the BT/TT leprosy patients and healthy contacts of leprosy patients will have larger indurations at both *M. leprae*-derived antigen sites, and a variable reaction at the site of the tuberculin/PPD. The non-contacts, BL/LL leprosy patients, and tuberculosis patients will have smaller indurations at all leprosy skin test sites, and a variable reaction at the site of tuberculin/PPD. Finally, the tuberculosis patients will react with a large induration at the tuberculin/PPD site.

4.1 Rationale for Design Features

The rationale behind this study design is to ensure the safety of each antigen at both dosages in a low risk population (non-contacts, who are healthy individuals without known exposure to leprosy), before moving to a higher risk population (contacts of leprosy patients, clinical leprosy patients, and tuberculosis patients). Tuberculin/PPD will be used as a control to measure reactivity of not only BCG vaccination, but also tuberculosis exposure, and exposure to non-pathogenic environmental mycobacteria to some extent. In all subjects, we will be comparing diameters of the erythema and induration reactions to tuberculin/PPD with those to the new leprosy skin test antigens. Reactogenicity of antigens (or lack thereof) in non-contacts will establish the baseline reaction and aid in establishing a “cut off” point to define a “positive” skin test. Saline will serve as a diluent control in Stage A and B only. Specificity and sensitivity will be determined for each antigen and dosage.

4.2 Description of Study Groups

Stage	Study group	Number of groups	Sample size/group	Total sample size	No. injection sites	Estimated completion time
A	Non-contacts	2	5	10	4	1 month
B	Non-contacts	2	45	90	4	3 months
C-1	Contacts (co inhabitants) of BL/LL leprosy patients<br>BL/LL leprosy patients<br>BT/TT leprosy patients<br>Tuberculosis patients	4	20	80	3	3 months
C-1b	Contacts (co-inhabitants) of BL/LL leprosy patients<br>BL/LL leprosy patients<br>BT/TT leprosy patients<br>Tuberculosis patients	4	20	80	3	3 months

4.3 Approximate Time to Complete Study Enrollment

The total estimated time to complete the protocol is 17 months, however, this is not a continuum, because safety data will be collected after each stage of the study (A, B, C-1, and C-1b), reviewed by the SMC, and the resultant SMC report will be forwarded to DMID, NHRC, and CSU IRB for approval. Estimated time for this review and approval process is 4 months (minimum) per stage, resulting in an additional 16 months. Thus, the total estimated time to complete all stages of this Phase II Clinical Trial is 33 months (or 2.75 years).

4.4 Sequence and Expected Duration of Subject Participation

Subject participation for each stage of the trial will be 7 days (± 1 day). If induration > 10mm at any injection site is observed during the first 7 days, involvement in the study will last for 28 days (± 3 days) or until all reactions are resolved or stabilized. Total time involvement for each participant is approximately 5.0 hours, as follows:

Trial Period	Description	Stage					Est. Duration (hours)
		A	B	C1a	C1b		
Day –10 to –1	Description of the study to allow informed consent	X	X	X	X		1.00
Day 0	Informed consent process<br>Screening:<br>Health Questionnaire/History Form<br>Physical Examination<br>Eligibility Checklist/Enrollment<br>Blood Sampling	X<br>X<br><br><br>X 	X<br>X<br><br><br>X 	X<br>X<br><br><br>X<br>X	X<br>X<br><br><br>X<br>X		0.50<br>1.50<br><br><br>0.25<br>0.25
Day 0	Administration of antigen<br>Reaction recorded ~15min and 30 min (for stage C1b only)	X	X	X	X		0.25<br>0.25
Day 2	Reading skin tests	X					0.25
Day 3	Reading skin tests	X	X	X	X		0.25
Day 7	Reading skin tests		X	X	X		0.25
Day 28	Reading of positive reactors	X	X	X	X		0.25
Total Time							
	Study participants						5.0

4.5 Methods for Collecting Data for Assessment of Study Objectives

Administration of antigens and reading of induration will normally be performed by separate teams, although it will be acceptable for an administrator to also be a reader of the same subject. Administrators and readers will be blinded to the antigen identity at each site. Each antigen site will be read and recorded by two independent readers at ~15 min, 45-51 h and 69-75 h for Stage A, and ~15 min, 69-75 h, and 6-8 days for Stage B, ~15 min,~30 min (for stage C1b only), 2-4 days, and 6-8 days for Stage C. The method for reading will be as follows: The site will be gently palpated, the limits of the induration determined with the fingers, and the largest transverse diameter will be measured with a soft, flexible, transparent ruler by two independent readers. The maximal diameter will be recorded by each reader, and an average of the two will be used. Readings from both readers will be analyzed for correlation (the most experienced administrator/reader will be used as the 'gold standard'). In the case that there are systematic reading errors on the part of the second reader, the reader will be given more supervision. In the case that the second reader shows 'terminal digit preference' in his/her readings (i.e., his/her readings always end in an even or odd number), further training will be given without explaining to the reader the reason for the additional training, in order to avoid correcting one bias by potentially introducing another [adapted from "Guidelines for Conducting Skin Test Surveys in High Prevalence Countries," issued by the International Union Against Tuberculosis and Lung Disease (24)]. Erythema will also be measured (by ruler) and recorded at each time point.

Any subject with an induration greater than 10 mm at either the 45-51 h or 69-75 h reading for Stage A, and 69-75 h or 6-8 day readings for Stage B, and 2-4 day or 6-8 day readings for Stage C, will be asked to return at 25-31 days for follow-up. Those subjects who need to return at day 25-31 will have their study/subject number posted on the bulletin board of the appropriate college/campus (primarily healthy non-contacts in all stages) and/or will be given instructions and a card indicating the date of their next visit (primarily patients and contacts of leprosy patients in Stage C) to serve as a reminder, without revealing the identity of the subject. Subjects experiencing any persistent reaction or adverse event will be followed until the resolution or stabilization of symptoms. Adverse events will be graded and recorded in the CRFs during each study visit.

Prior to study enrollment, all participants will have provided informed consent after having been told about the study and what is expected for participation. Source documents pertaining to individual study subjects will include the Skin Test Reading forms and Case Report Forms (CRFs) as follows:

- Eligibility Checklist
- Informed Consent Form
- Demographics/Medical History Form
- Antigen Administration Form
- Follow-up Examination and Reaction Form
- Skin Test Reading Forms for each time point
- Study Termination Form
- Adverse Event Form
- Serious Adverse Event Form

Most data on the CRF will be obtained directly from the participant. Some clinical data relating to the treatment history of the participant for tuberculosis (TB) or leprosy as applicable, will be confirmed from their medical records following completion of a separate Informed Consent Document. Only the information specified in the CRF will be derived from these records. Data from the CRFs and Skin Test Reading forms will be entered into electronic files at AH, and sent monthly via Compact Disk (CD) to the Data Control Center (DCC), EMMES Corporation, where the data will be analyzed by uploading onto the EMMES computer database. As a quality control measure, the data will be checked for anomalies and inconsistencies through The EMMES Corporation's Data Integrity Evaluation System. The Data Manager will send a Data Resolution Form (DRF) outlining missing values, out-of-range values, and other data anomalies to the central clinical site for reconciliation of data discrepancies.

4.6 Covariates

In addition to the primary and secondary outcome measures described above, potential confounding factors will be evaluated, as follows:

- Healthy non-contacts: Age, sex, BCG status, and tuberculin/PPD reaction

- Contacts of leprosy patients: Age, sex, BCG status, type of contact, length of contact, characteristics of index case, and tuberculin/PPD reaction
- Leprosy patients: Age, sex, BCG status, type of leprosy, smear result, length of treatment, history of reaction, and tuberculin/PPD reaction
- TB patients: Age, sex, BCG status, length of treatment, and tuberculin/PPD reaction

First, the influence of these confounders on induration size and proportion positive will be evaluated at a univariate setting. Factors found to be significant at univariate level will be entered into the multivariate model. These analyses will not only be used to test the differences between groups, but will also allow us to estimate the adjusted geometric mean reaction size for each group (after transformation to the natural reaction size). The multivariate logistic regression model for “positive reaction“ outcome will also allow us to obtain sensitivity and specificity, both adjusted for important confounding factors.

4.7 Interim Analysis Plans

Upon the completion of each stage, The EMMES Corporation will analyze and compile data into a safety report to be reviewed by the SMC. Data from Stage A will be reported “blinded,” since Stage A is merely a ramping stage for Stage B. Following Stage B, both Stage A and B will be unblinded and analyzed together. Upon review of Stages A/B, C-1 and C-1b, the SMC will compose a report to be sent to DMID, NHRC, and CSU IRB. In all cases of unblinding, the identity of antigens will only be made available to key study personnel.

4.8 Safety Oversight, per DMID Guidelines; SMC

An on-site, qualified physician will serve as an Independent Safety Monitor (ISM). This individual will be responsible for reviewing all adverse events (AEs) and serious adverse events (SAEs) and reporting the findings to the Principal Investigator or Subinvestigator, who will forward the report to PPD Development Inc., DMIDs pharmacovigilance contractor, and DMID. The ISM will also serve as a member of the SMC. The SMC consists of 4 physicians with extensive experience in leprosy and/or tuberculosis disease, and 1 biostatistician. The purpose of this committee is to review all serious adverse events that occur in the study, based on information that will be provided. They will then analyze all serious adverse events (SAEs), and make recommendations to DMID, NHRC, and CSU IRB, as to whether the study should be stopped or the protocol altered, as necessary. Reasons to stop the study will include, but not necessarily be limited to, any severe anaphylactic reaction to one or both of the new skin test antigens. Severe ulceration and necrosis at the skin test antigen sites or

urticaria, or an anaphylactic reaction would also be reasons for stopping the study temporarily for review and advice before potentially proceeding.

5 STUDY POPULATION

5.1 Selection of the Study Population

Three campuses have been identified to perform Stage A and B: Lalitpur Nursing Campus, Sanepa, Kathmandu; The Central Department of Microbiology, Tribhuvan University, Kirtipur, Kathmandu; and, The Institute of Medicine, Tribhuvan University, Maharajgunj, Kathmandu. Four additional campuses have been identified to perform Stage C: Patan Hospital TB Clinic; Lalgadh Leprosy Hospital; Green Pastures Leprosy Hospital; and AH. Other campuses/hospitals may also be selected to participate. All participants will be residents of Nepal, including expatriates from India, because they have been exposed to similar environments, as opposed to expatriates from other countries. Of these 260 individuals, 100 will be healthy non-contacts, 40 will be cases of leprosy from Lalgadh Leprosy Hospital, Green Pastures Leprosy Hospital, and AH, 40 will be healthy contacts of BL/LL and/or BT/TT leprosy patients, and 40 will be tuberculosis patients recruited from the Patan Hospital TB clinics. A leprosy contact is defined as a co-inhabitant of a leprosy patient for a duration of at least six months, and within 6 months of enrollment into the study or a person who has been professionally exposed to leprosy for a duration of at least 5 years and within 6 months prior to enrollment in the study. Enrollment of contacts of leprosy patients for Stage C-1 and Stage C-1b will include only co-inhabitants of BL/LL leprosy patients. The duration of contact with the index case, and details of the index case will be recorded in the CRFs. The volunteers will be free of intercurrent tuberculosis, and will not have been taking immunosuppressive therapy for at least 4 weeks prior to study enrollment.

5.1.1 Percentages of Women, Minorities and Children

Males and females are to be recruited for this study, with not less than one-third of either gender in the overall study. Race and ethnic demographics will be collected, however specific targeted numbers of subjects of a specific minority (excepting women) were not specified in the original approved protocol. Upon moving into a Phase III trial, specific minorities will be targeted to comply with NIH Guide Notice NOT-OD-02-001, October 9, 2001. On the other hand, minors are excluded, because the research may involve greater than minimal risk and there is no direct benefit to the participants at this early stage of the study, thus not satisfying additional federal regulations for inclusion of children outlined in 45 CFR 46, subpart 401-409 and NIH Guide Notice 98-024, March 6, 1998.

5.1.2 Losses to Follow-Up

Our experience in this region of Nepal suggests that no more than 10% of subjects will be lost to follow-up. We are not planning to administer skin tests to additional subjects should some of the subjects not return for their first or later readings.

5.1.3 Subject Recruitment and Retention

In all stages of the study, the process of recruitment will be the same: explanation by means of a recruitment talk (in the case of healthy controls recruited at campuses), or individual explanation using a flip chart and the consent form (in the case of patients or patient contacts in leprosy/TB clinics). A recruitment talk will be delivered at campuses where recruitment of healthy subjects into Stages A and B of the study is planned. The talk will be presented in Nepali by a senior member of the research team, or in English and simultaneously translated into Nepali. It is anticipated that this talk will be given on the day that informed consent will be sought and screening tests will be performed, approximately 1 week prior to administration of the skin test antigens. The talk will be followed by a question period when all students can ask the investigators about any concerns they may have. Recruitment for Stage C-1 and C-1b will be slightly more informal. Study investigators will explain the research study to interested individuals using a flip chart. These individuals will also be given consent forms in their own language (Nepali or Hindi). In the case of illiterate subjects, the information will be read to them by a study staff member. Subjects will be compensated for their time and inconvenience by receiving a small gift valued at approximately US$11.

5.1.4 Screening Subjects

Stages A and B will be performed at the following campuses in Nepal: Lalitpur Nursing Campus, Sanepa, Kathmandu; The Central Department of Microbiology, Tribhuvan University, Kirtipur, Kathmandu; and, The Institute of Medicine, Tribhuvan University, Maharajgunj, Kathmandu. Other campuses/hospitals may also be selected to participate. A total of 100 (10 for Stage A and 90 for Stage B) healthy, non-pregnant Nepali volunteers (including expatriates from India) from the above college campuses, with no known exposure to leprosy or TB, will be studied. A tuberculin/PPD test will not be performed as part of the screening process. Volunteers will be contacted directly and invited to participate after an informational talk about the research study given by the investigators at the listed campuses, one week prior to antigen administration. All subjects entering the study will be asked to read or have read to them a detailed consent form, which they will sign. Fifty individuals (5 for Stage A, and 45 for Stage B) will be randomly selected for testing MLSA-LAM and the other fifty individuals (5 for Stage A, and 45 for Stage B) will receive MLCwA. It is expected that, as with tuberculin/PPD, the two new leprosy skin test antigens will evoke negative responses in healthy, unexposed subjects.

Stage C will be performed at the following campuses in Nepal:

- AH, Kathmandu
- Lalitpur Nurses Campus, Sanepa, Kathmandu
- The Central Department of Microbiology, Tribhuvan University, Kirtipur, Kathmandu
- The Institute of Medicine, Tribhuvan University, Maharajgunj, Kathmandu

- Patan Hospital TB Clinic, Kathmandu
- Lalgadh Leprosy Hospital, Lalgadh
- Green Pastures Leprosy Hospital, Pokhara.

Other campuses/hospitals may also be selected to participate. All subjects entering the study will be asked to read (or have read to them) and sign a detailed consent form. Of these 160 individuals, 80 will be cases of leprosy from the AH (40 BL/LL and 40 BT/TT leprosy patients), 40 will be healthy contacts of BL/LL leprosy patients , and 40 will be tuberculosis patients recruited from the Patan Hospital TB clinics. Participants other than TB patients will be free of intercurrent tuberculosis, and will not have taken immunosuppressive therapy for at least 4 weeks prior to study participation. TB patients must have completed the intensive phase of anti-tuberculosis therapy and taking anti-tuberculosis medicines at the time of testing. Volunteers will be contacted directly and invited to participate after an informational talk about the study given by the investigators approximately 1-2 weeks before study initiation at the listed campuses or at hospital wards, or in the case of tuberculosis patients, given during the weekly clinic or hospital ward visits.

Each volunteer who agrees to enter the study and provides informed consent will be assigned a subject screening number. This number and the name of the volunteer will be recorded in a log kept by the Study Coordinator (SC). In order to protect the privacy of each participant, only their subject number will be used on all CRFs and data analysis reports.

To assess eligibility, volunteers will be asked a series of health related questions, given a general physical exam and an examination for signs of leprosy, tested for pregnancy (if female), demographic information collected, and have their BCG scar measured (if present). These data are recorded on the Screening Form and Demographics/Medical History Form, and used to assess eligibility for the study. The general medical exam includes vital signs and body weight. The pregnancy test involves obtaining a urine sample and performing a simple in-clinic test purchased from a certified vendor. The whole body exam includes looking for skin lesions associated with leprosy, and checking major nerve trunks for enlargement at standard sites, including the greater auricular nerve, ulnar nerve at elbows, radial nerve at wrists, lateral popliteal nerve at the back of the knees, and posterial tibial nerve at the heel. If a healthy volunteer or contact subject should show a lesion suspicious for leprosy or signs of tuberculosis, they will be excluded from the study, and treated as per standard hospital procedure. At the time of physical examination for leprosy, subjects will be examined for the presence of a BCG scar and the scar, if present, will be measured across the greatest diameter and recorded on the Demographics/Medical History Form.

If the participant is a healthy contact of a leprosy subject, additional information will be collected on the Demographics/Medical History Form. Such information will include: type of contact (professional or household); relationship to the index case; index case leprosy type, initial skin smear results; treatment history including months of treatment and length of contact. If the subject has multiple household contacts, information will be recorded on a Supplemental Household Contact form.

5.1.5 Enrollment

Eligibility for enrollment is determined by data collected on the Screening Form, and Demographics/Medical History Form. The medical officer, nurse, or paramedic will review the data and complete the eligibility checklist after the physical examination has been completed. If eligible for the study, the subject will be assigned a study subject number. If a subject is not enrolled in the study, source documentation for the screening procedure will be maintained at the site and CRFs will not be submitted to the DCC.

5.1.6 Randomization

To minimize observer bias for placement of the skin test antigens, templates have been developed for Stage A and B, Stage C-1 and C-1b. Each subject in Stage A or B will be randomly assigned one of the templates created for their respective antigen group (MLSA-LAM or MLCwA) upon enrollment into the study. Subjects in Stage C will be assigned one of the templates designed for Stage C-1 or C-1b upon enrollment into the study to randomize placement of the antigen administration sites. The template number will be recorded on the subject's CRF and in the data logs. Antigen administrators will inject subjects according to their assigned template. Following administration, independent skin test readers will see the subjects in a random order at ~15 min, 45-51h and 69-75 h for Stage A, 69-75 h and 6-8 days for Stage B and 15min, 30 min (for stage C1b), 2-4 days and 6-8 days for Stage C, and, if needed, 25-31 days for all stages.

5.2 Subject Inclusion Criteria

The subject must meet the following inclusion criteria to participate in this study:

<u>All Subjects</u>

- Between the ages of 18 and 60 years old
- Male or female; not less than 30% for one gender
- Agree to participate in the study after verbal explanation by the physician and nurses, as indicated by signing an informed consent form
- Weight greater than 30 Kg (female) and 38 Kg (male)
- Available for skin test readings
- Nepali residents, including expatriates from India

<u>Healthy, Non-Contacts</u>

- Healthy (determined by history and physical examination)
- No household or working contact with tuberculosis or leprosy patients

<u>Contacts of Leprosy Patients</u>

- Healthy (determined by history and physical examination)

- Household contact of a person with leprosy for at least 6 months duration, and within 6 months of this study, or a person professionally exposed to leprosy for at least 5 years duration, and within 6 months of this study

Persons with Leprosy

- Having one or more of the following symptoms:
  - Hypopigmented or erythematous skin lesion(s) with definite loss of sensation
  - Damage to the peripheral nerves as demonstrated by palpable thickening with or without impairment of sensation and/or weakness of the muscles of hands, feet or face
  - Presence of acid-fast bacilli in slit skin smears
  - Histological changes diagnostic of leprosy in skin biopsy
- Receiving standard MDT treatment for leprosy or completed treatment for leprosy no more than 4 years prior to study enrollment

Persons with Tuberculosis

- Having active tuberculosis as defined by one of the following:
  - Extra-pulmonary tuberculosis if confirmed by culture
  - Pulmonary tuberculosis, defined as:
    - Having a history of a productive cough of more than 3 weeks duration that may be accompanied by night sweats, loss of appetite, haemoptysis, weight loss, chest pain, or shortness of breath, and
    - Having one or more of the following diagnostic criteria:
      - Sputum smear-positive, defined as one or more of the following: at least 2 of 3 successive sputum samples positive for acid-fast bacilli by microscopy; or at least one sputum specimen positive and x-ray abnormalities consistent with pulmonary tuberculosis; or at least one positive sputum specimen that is culture positive for *Mycobacterium tuberculosis*
      - Sputum smear-negative, defined as three sputum specimens negative for acid-fast bacilli but with x-ray evidence consistent with pulmonary tuberculosis and that does not clear with non-tuberculous antibiotics; or three sputum samples negative for acid-fast bacilli by microscopy but culture positive for *Mycobacterium tuberculosis*
- Completed the intensive phase of chemotherapy for tuberculosis, but still undergoing the continuation phase of therapy

5.3 Subject Exclusion Criteria

All subjects

- Pregnant (as determined by a urine pregnancy test performed on females of child-bearing age on Day 0, prior to admission into the study) or lactating females

- Currently on oral corticosteroid or other immunosuppressive treatment
- Cancer, diabetes, or other chronic illness
- Extra-pulmonary tuberculosis not confirmed by culture
- Known hypersensitivities or allergies
- Expatriates other than those from India
- Participation in an earlier stage of this study
- Concurrent participation in another clinical trial

Healthy, Non-Contacts

- History of treated tuberculosis or leprosy
- Clinical signs of leprosy or tuberculosis
- Known contact with persons with leprosy or tuberculosis

Healthy Contacts of Leprosy Patients

- History of treated tuberculosis or leprosy
- Clinical signs of leprosy or tuberculosis

Person with Leprosy

- Leprosy patients in reversal reaction or erythema nodosum leprosum (ENL) reaction or those being treated with corticosteroids or thalidomide for these conditions
- History of treated tuberculosis
- Clinical signs of tuberculosis
- Completed full course of standard MDT for leprosy more than 4 years prior to study enrollment

Persons with Tuberculosis

- History of treated leprosy
- Clinical signs of leprosy
- Completed full course of standard tuberculosis treatment
- Known contact with leprosy patients

HIV testing will not be performed on any subject in this study since the prevalence of HIV seropositivity is currently only 0.2% in Nepal.

6 RANDOMIZATION/ENROLLMENT/BLINDING PROCEDURES

Upon successful completion of the screening procedures, the SC will assign each participant a unique subject number indicating enrollment. Each subject will be assigned to an antigen administration template derived from a randomization sequence provided by EMMES Corporation. This sequence randomly associates one of the templates to a study number, and is generated using a fixed blocked size to ensure template balance within each block. In order to preserve the randomization, the subject numbers must be assigned sequentially.

Should some of the subjects not return for the first or later readings, there is no plan to administer skin tests to additional subjects. The analyses presented in Section 13.6, “Sample Size Considerations”, show that the study will be able to meet the primary statistical objectives, even should up to 10% of the subjects be lost to follow-up.

The person administering the antigens will be blinded as to their identity. Moreover, there is no recognized medical benefit of knowing the identity of an antigen in the event a subject must be treated for an adverse event. Therefore, unblinding will not occur during the study unless recommended by the SMC and approved by the DMID Project Officer (PO).

Adverse events, including serious adverse events, will not in and of themselves provide a cause for unblinding. The ISM may temporarily suspend enrolling further subjects pending discussion with the DMID PO and SMC. The Principal Investigator will report all SAEs to PPD Development Inc., DMIDs pharmacovigilance contractor, who will then forward the information to the DMID Study Coordinator for distribution to the DMID PO and the SMC to determine if the antigens should be unblinded, and if the study needs to be amended as a consequence of the SAE. In all cases of unblinding, the identity of antigens will only be made available to key study personnel and SMC members as necessary.

7 STUDY PROCEDURES/EVALUATIONS

7.1 Clinical Evaluations

7.1.1 Medical History

On day 0, participants will give informed consent to be a participant in this study. They will be interviewed to obtain information regarding demographics, and some parts of their medical history. Medical history collected from each participant will vary according to the study group or subject group and may include additional forms (see below). Leprosy patient and tuberculosis patient subjects will have specific relevant additional information recorded from their medical records. Healthy contacts of leprosy patients will identify their contact who has leprosy (possibly a non-study subject), and the leprosy patient will be asked to provide written consent allowing investigators to view and record relevant information from their medical records (as described below).

<u>Specific medical history information obtained</u>:

1. Healthy Non-Contacts:
    - BCG scar (presence or absence, number of scars, and diameter of largest scar)

2. Healthy Contacts of Leprosy Patients:
    - BCG scar (presence or absence, number of scars, and diameter of largest scar)
    - Type of contact (household or professional); if the subject has multiple contacts a supplemental form is used to collect information on each contact.
        - Household contact
            - Index case number
            - Relationship
            - Leprosy type
            - Initial skin smear results
            - Treatment history (months of treatment)
            - Length of contact (years and months)
        - Professional contact
            - Place of work
            - Type of work (doctor, nurse, paramedic, physiotherapist, other)
            - Length of exposure (years and months)

3. Persons with Leprosy:
   - BCG scar (presence or absence, number of scars, and diameter of largest scar)
   - Medical record number from the subject's chart
   - Hospital name
   - Leprosy clinical classification (TT, BT, BL, LL); Patients will be classified according to Ridley-Jopling, but will only be included if they fall into either BL/LL or BT/TT categories.
   - Treatment type (PB MDT or MB MDT)
   - Length of treatment (months)
   - Date released from treatment
   - Initial skin smear result
   - History of reactions (Type I or ENL); Type I or reversal lepra reaction is an example of Type IV hypersensitivity (allergic) reaction. Erythema nodosum leprosum (ENL) is a Type 2 lepra reaction exemplified by humoral hypersensitivity. It is not associated with alteration in the CMI

4. Persons with Tuberculosis:
   - BCG scar (presence or absence, number of scars, and diameter of largest scar)
   - Medical record number from the subject's chart
   - Hospital name
   - Type of TB and smear result (pulmonary TB, non-pulmonary TB)
   - TB confirmed by culture
   - Type of Treatment
     - Type I - New sputum smear-positive patients, short course regimen
     - Type II – Retreatment patients
     - Type III – New smear-negative and extra-pulmonary patients, short course regimen
   - Length of treatment (months)

7.1.2 Medication(s) History

A complete medications history from each subject will be collected for this study. Information regarding current prescription or over-the-counter medications is not critical, however, individuals currently on oral corticosteroid or other immunosuppressive treatment will not be eligible for enrollment. Leprosy patients currently taking MDT and tuberculosis patients on chemotherapy are eligible for study participation. Assessment of eligibility is dependent on these criteria.

It should be noted that if an SAE occurs, the subject will be queried about concomitant medication(s) he/she had been taking since the initiation of the study. If the subject is taking medication, all medications will be recorded on page 2 of the SAE form.

7.1.3 Physical Examination

Subjects will undergo three types of examination on day 0 of the study: (1) general medical examination; (2) examination for signs of clinical leprosy; and, (3) examination of the BCG vaccination scar. First, the general medical examination will measure body weight and vital signs (temperature, pulse, and blood pressure). For all female subjects, a sample of urine will be taken and a pregnancy test performed. Second, the examination for clinical signs of leprosy will include checking the whole body of each subject for skin lesions, and checking major nerve trunks for enlargement at the standard sites indicated for leprosy: greater auricular nerve in the neck, ulnar nerve at elbows, radial nerve at wrists, lateral popliteal nerve at the back of the knees, and posterial tibial nerve at the heel. If a healthy volunteer, TB patient, or contact subject should show a lesion suspicious for leprosy, a leprosy physician will re-examine the subject, and those felt to have signs of early leprosy will be excluded from the study. Information gathered from the general exam and leprosy exam will be recorded on the screening form. Finally, at the time of examination for leprosy, subjects will be examined for the presence of a BCG scar. If present, the largest scar will be measured across the greatest diameter and results recorded on the Demographics /Medical History Form.

7.1.4 Counseling

If during the physical examination, a healthy volunteer, TB patient, or contact of a leprosy patient should show a lesion suspicious for leprosy, a leprosy physician will re-examine the subject. Individuals felt to have signs of early leprosy will be excluded from the study. Counseling, education, and free anti-leprosy treatment will be provided to these volunteers by AH (or the leprosy treatment facility nearest to the patient) outside of this protocol.

7.1.5 Reactogenicity Assessments

Three forms will be used to record reactogenicity: Follow-up Examination and Reaction Form, Adverse Event Form, and Serious Adverse Event Form, as described below.

SKIN				
Reactogenicity<br>Form(s) used	Grade 1<br>Reaction Form	Grade 2<br>Reaction Form	Grade 3<br>Adverse Events and Reaction Forms	Grade 4<br>SAE, Adverse Events and Reaction Forms
Mucocutaneous	erythema;<br>pruritus	diffuse, maculo papular rash, or dry desquamation	vesiculation or moist desquamation or ulceration<br><br>infection at injection site	exfoliative dermatitis, mucous membrane involvement or erythema, multiforme or suspected Stevens-Johnson, or necrosis requiring surgery<br><br>anaphylaxis<br><br>systemic infection
Induration	< 15mm	15-30 mm	>30mm	N/A
Pain at Injection Site	Barely noticeable at injection site	Slightly uncomfortable at injection site`	Moderately uncomfortable at injection site	Prevents minimal activity
Erythema	< 15mm	15-30 mm	>30mm	N/A
Edema	< 15mm	15-30 mm	>30mm	N/A
Rash at Injection Site Other than Erythema	< 15mm	15-30 mm	>30mm	N/A
Pruritus	slight itching at injection site	moderate itching at injection extremity	itching over entire body	N/A

7.1.6 Review of Diary Cards

The Diary Card is used only for subjects enrolled in Stages A and B to monitor reactions to the antigens between visits. The subject is to record any pain, swelling, redness, itching, blistering, or swollen lymph nodes that occur on the day of the skin test and for the next three days (Stage A) or 7 days (Stage B) following the skin test. The Diary Card will be submitted to the SC at the 7 day reading for review by the SC and/or Medical Officer (MO). The study volunteer will be interviewed and examined as necessary, at that time. Additional study personnel will be notified accordingly. Data from the Symptom Diary will not be entered into the study database, unless relevant information such as an AE is recorded. Any findings of relevance will be entered into the appropriate CRF (Follow-up Form, and/or AE Form). Diaries will be stored by the SC for a period of 2 years after the end of the trial, as per FDA IND requirements. At the end of the specified study period, the Diary Cards will be destroyed.

7.2 Concomitant Medications/Treatments

7.2.1 Permitted Medications/Treatments

- Permitted medications for leprosy and tuberculosis therapy.
  - Anti-leprosy medications
    - Multiple Drug Therapy (MDT): Dapsone, Rifampicin, and Clofazimine (or a variation of these three medications)
    - Minocycline, Ofloxacin, and/or Rifabutin
  - Anti-tuberculosis medications
    - Continuation treatment phase: Rifampicin, Isoniazid, and Pyrazinamide
- Rescue medications
  - Ringers solution (for fainting)
  - Topical antibiotics/antiseptics (for infection and abscess, as needed)
    - Mild: Soframycin or Betadine
  - Metaclorpramide or Prochlorperazine (for nausea)
  - Pheniramine Maleate (for urticaria)
  - Salbutamol and Hydrocortisone with Ringers Lactate (as needed for mild to severe cyanosis)

7.2.2 Prohibited Medications/Treatments

- Corticosteroids (prednisolone) and other immunosuppressive treatments within 4 weeks before enrollment in the study
- Thalidomide for treatment of reversal reaction or ENL in leprosy patients

7.3 Laboratory Evaluations

7.3.1 Clinical Laboratory Evaluations

7.3.1.1 Pregnancy test

On day 0, women will be asked to provide a fresh sample of urine for this test. The test is for human chorionic gonadotrophin (hCG) in the urine, and will be performed according to manufacturer's instructions. All subjects with positive test results will be excluded from the study.

7.3.2 Special Assays or Procedures

7.3.2.1 Laboratory Assays

Two laboratory tests to measure antibody presence or absence to a leprosy specific antigen, and to measure cell mediated immunity to

both skin test antigens, will be performed at AH. A total of 5ml heparinized blood and 3ml of whole blood (to prepare serum) will be collected from each subject on day 0, prior to antigen administration, using a syringe. Blood samples will be labeled with the proper subject study ID number and date, and transported at 22ºC ± 5ºC to AH. The anti-PGL-I antibody assay will be performed using the lateral flow immunodiffusion test (Rapid PGL-I Antibody Test). This test is of limited use for the avowed objectives, because it provides a measurement of humoral immunity rather than cell mediated immunity. Moreover, it is most sensitive for multibacillary (BL/LL), the more reactive form of leprosy, but is of little value at the paucibacillary (BT/TT) end, or early stages of leprosy. Still it is a specific test and is therefore included. In addition, the QuantiFERON-CMI test will be performed using at a minimum the two leprosy skin test antigens and tuberculin/PPD. This test will measure the IFN-γ response following stimulation of T-cells in blood against the antigens, providing data which may be compared to the DTH response.

7.3.3 Specimen Preparation, Handling, and Shipping

7.3.3.1 Specimen Preparation, Handling, and Storage

Blood samples will be labeled with the subject study ID and the date of collection. They will be placed in a rack to prevent breakage, incubated at 22ºC ± 5ºC in a portable incubator, and transported by vehicle to AH to run laboratory assays (QuantiFERON assay will be performed within 6-12 hours of blood collection and the Rapid PGL-I Antibody test will be performed within 3 days of blood collection).

Whole blood samples will be completely expended by AH at the time of performing the QuantiFERON-CMI assay. Remaining serum samples following Rapid PGL-I Serology testing will be stored at -20°C,. Upon completion of the study, all remaining serum samples will be autoclaved and discarded.

8 STUDY VISIT SCHEDULE

8.1 Screening Visit

Following recruitment talks (day –10 to -1), interested individuals will be asked to come to the clinic on a specified day and time on day 0 for a screening visit. They will be greeted by one of the leprosy healthcare fieldworkers recruited to help with this study. Each staff member participating in this trial will be a licensed paramedic who is both qualified and experienced in the administration of injections. Volunteers will be asked to provide written informed consent in their native language (Nepali or Hindi, as appropriate) for their participation after the research study has been re-explained to them.

A fieldworker will explain the consent form to the subject. A friend, family member, or staff member (not linked to the study) will witness the reading and signing of the consent form. The attending qualified staff member (fieldworker, SC, or investigator) on the Anandaban team will sign the consent form as the Investigator. In the case of illiterate subjects, the information will be read to them by a fieldworker, and the person who has read the form to the volunteer must complete the associated declaration at the bottom of the consent form. A copy of the completed consent form will immediately be given to the subject, and the SC will keep the original document in the study file at AH.

After signing a consent form, each subject will be issued a sequential screening subject number (unique subject number preceded with an "S") before beginning the screening process. This number and the name of the volunteer will be recorded in the Screening Log, which will be retained only by the SC. Identification of subjects on all case report forms and data analysis reports will be by screening number, subject number, and subject initials in order to protect the privacy of each participant.

Subjects will then be asked to answer a series of questions about their health and demographics and undergo a physical examination for general health, signs of clinical leprosy, and BCG vaccination as described in Section 7.1, "Clinical Evaluations". At this time, women are also given a urine pregnancy test. Healthy contacts of leprosy patients will be asked questions about their interaction with leprosy patients, while leprosy patient index cases of household contacts will be asked to sign a Medical Records consent form to allow staff member's permission to confirm or complete information on the medical history form. Leprosy and TB patients in the study will be asked questions about their clinical disease, and medical records will be checked to confirm and/or complete this information, as needed. Screening tests must be completed on the day of enrollment (day 0).

8.2 Enrollment/Baseline Visit

8.2.1 Enrollment

Once all of the examinations have been completed, the MO, nurse, or paramedic will assess the eligibility of subjects from screening visits using the Eligibility Checklist. If a subject meets study criteria (outlined in Section 5.2 and 5.3) and is available for each skin test reading, the subject will be enrolled in this study. This document summarizes responses recorded in the Health Questionnaire section of the Screening Form, and Demographics and Medical History Form, relative to the inclusion and exclusion criteria. The Eligibility Checklist directs the staff member on whether or not to exclude a subject from the study. In brief, if answers from study subjects to the following inclusion criteria (shown as "X" in the example below) are "no", then the subject must be excluded from the study:

Inclusion Criteria	Healthy unexposed	Healthy Contact	Leprosy Patient	TB Patient
Between the ages of 18 and 60, Nepali resident including expatriate from India	X	X	X	X
Agreed to participate in the study and has signed Informed Consent	X	X	X	X
Weight greater than 30kg for female, 38kg male	X	X	X	X
Available for skin test readings	X	X	X	X
Healthy as determined by history and exams	X	X		
Household contact with a person with leprosy for at least 6 months, or at least 5 years professional exposure, within not more than 6 months of being enrolled in the study		X		
One or more symptoms of leprosy (as defined in Section 5.2, "Subject Inclusion Criteria")			X	
Receiving treatment for leprosy, or having received treatment within 4 years of enrollment in the study			X	
Not on immunosuppressive therapy or 4 week washout period since last therapy treatment			X	
Active TB (as defined in Section 5.2, "Subject Inclusion Criteria")				X
Completed intensive phase of chemotherapy for TB, but still in continuation phase				X

If answers from study subjects to the following exclusion criteria (shown as "X" in the example below) are "yes" then the subject must be excluded from the study:

Exclusion Criteria	Healthy unexposed	Healthy Contact	Leprosy Patient	TB Patient
If female, pregnant or lactating	X	X	X	X
Currently taking oral corticosteroids or other immunosuppressive treatment, or taken within 4 weeks prior to enrollment in the study	X	X	X	X
Cancer, diabetes, or other chronic illness	X	X	X	X
Extra-pulmonary TB, not confirmed by culture	X	X	X	X
Known hypersensitivities or allergies	X	X	X	X
Known contact with a person with leprosy or TB	X			(X) (Contact with leprosy)
Exhibits clinical signs of TB (as defined in Section 5.2, "Subject Inclusion Criteria")	X	X	X	
History of treated TB	X	X	X	
Reversal or ENL reaction or being treated with corticosteroids or thalidomide for these rxns			X	
Completed a full course of therapy for leprosy more than 4 years prior to study enrollment			X	
Completed a full course of standard TB treatment				X
History of treated leprosy	X	X		X
Exhibits clinical signs of leprosy (as defined in Section 5.2, "Subject Inclusion Criteria")	X	X		X
Participation in another clinical trial	X	X	X	X
Expatriate from any country except for India	X	X	X	X
Participated in Stage A or B or current study	X			

Eligibility is then assessed as "yes" or "no" by the answers to the questions listed above. If the subject is eligible, the subject is assigned a unique subject identification number and given a subject card. They will be required to produce this card on each occasion for reading, so that their case report forms

can be drawn. If by chance the subject forgets to bring his/her study card, the SC will look up the subject ID number in the Screening Log, and issue a new card. If the subject is not eligible, the reason (did not meet enrollment criteria, decided not to participate, or other) is indicated on the Eligibility Checklist, which is signed and dated by the qualified staff person completing the form.

Measurement of baseline signs and symptoms relative to study intervention are not applicable in this study, since study intervention, (i.e., administration of leprosy skin test antigens), will occur on day 0, which is the same day as enrollment by the above mentioned parameters.

8.2.2 Administration of Study Product

Stage A : Ten healthy, non-pregnant Nepali volunteers (including expatriates from India) with no known exposure to leprosy or TB will be studied. A tuberculin/PPD test will not be performed before administration of skin test antigens. Five individuals will be randomly selected for testing MLSA-LAM, and the remaining five individuals will receive MLCwA. It is expected that as with tuberculin/PPD, the two new leprosy skin test antigens will evoke negative responses in healthy, unexposed subjects.

Each individual will receive a total of four injections. Two injections will be titrated doses of one of the two skin test antigens (in concentrations of 1μg and 10μg of protein content/ml in the sterile diluent, 0.9% NaCl, approved for human use, Abbott Laboratories). Additionally, each individual will receive one injection of 5 TU tuberculin/PPD (Tubersol®; Aventis Pasteur Inc., Swiftwater, PA), and one injection of diluent. All injections will be 0.1 ml in total volume from vials containing 0.5ml, 1ml or 2ml total. All injections will be made within 8 hours of a vial being opened. Cold storage (2-8ºC) will be maintained during this maximum 8 hour period. The concentration ranges to be tested are based on results of our Phase I study (Section 2.1.2, "Leprosy Skin Test Antigens, 1919-Today"), previous skin testing in humans with Rees' antigen (optimum dosage is 1 μg, i.e., 100 μl of 10 μg/ml), as well as our skin test results in guinea pigs.

The four test sites will be on the flexor surface of each forearm, about 3.5 inches and 5.5 inches below the bend of the elbow on each forearm. To minimize observer bias, templates for placement of the skin test antigens have been developed (see Section 6, "Randomization/Enrollment/Blinding Procedures"). Each subject will be randomly assigned one of the templates created for their respective test antigen group (MLSA-LAM or MLCwA). The assigned template number will be recorded on the subject's Case Report Forms and in the data log. The readers will see the subjects in a random order at ~15 min, 45-51 h, 69-75 h, and if needed, 25-31 days after antigen administration, and record results in a register by injection site (i.e., not seeing the template number at the reading time). Any reaction that persists will be followed-up until resolved or stabilized. This, along with the coding of the antigens, will prevent reader bias.

The skin of each forearm will first be cleansed with soap and water, swabbed with alcohol, and allowed to air dry. The test dose will be administered with a sterile 1 ml tuberculin syringe with a permanently attached, one-half inch,

27 gauge needle, calibrated in one-tenths. The rubber stopper of the vial will be wiped with a sterile piece of cotton, moistened with alcohol and allowed to dry. The vial will be inverted three times and a needle will then be inserted gently through the rubber septum, and 0.1 ml of the test antigen drawn into the syringe. The point of the needle will be inserted into the most superficial layers of the skin with the needle bevel pointing upward. If the intradermal injection is performed properly, a definite white bleb will rise at the needle point, about 10 mm (3/8 inch) in diameter. This will disappear within minutes. If there is significant leakage of antigen from the site of injection, the antigen will be re-injected approximately 1 inch medial to the original injection site. The Antigen Administrator will record the vial number used for each antigen, whether each antigen was administered, whether a bleb was present, if antigen leaked, and if so, if it was reapplied. No dressing will be necessary.

Each injection site will be marked with waterproof ink on the skin, to indicate the location of the injection. The subject will then be directed to a place to sit for the initial 15 minute wait until the necessary observations are conducted (see Section 8.3.1, " Initial Follow-up").

Stage B: Upon the completion of Stage A, and with approval from DMID, NHRC, and CSU IRB (no earlier than 4 days after the final reading of Stage A), ninety (90) healthy Nepali individuals (including expatriates from India) who are free of clinical signs of leprosy and did not participate in Stage A will be recruited for Stage B. Participants will be randomly assigned to receive MLSA-LAM or MLCwA (ratio 1:1) in the same blocked design as used in Stage A to ensure balance throughout the enrollment period. Antigens will be administered exactly as stated in Stage A, above (Section 8.2.2, "Administration of Study Product"), and readings will be taken at ~15 min, 69-75 h, 6-8 days, and if needed, 25-31 days.

Stage C: Upon completion of Stage B, and with approval from DMID, NHRC, and CSU IRB (no sooner than 4 days after the final reading of Stage B), 160 Nepali individuals (including expatriates from India) will be studied in Stage C. This stage is broken down into two parts: Stage C-1 and Stage C-1b, in which 80 subjects from four high risk groups will be studied in each Stage to measure the safety and/or reactivity of the antigens at the high dose (1.0µg) and low dose (0.1µg), respectively. In addition, each subject will provide a 5ml tube of heparinized blood for the laboratory personnel to perform a QuantiFERON-CMI test using leprosy antigens and a 3ml serum separator tube of blood to perform the Rapid PGL-I Antibody Test.

Stage C-1: In order to assess the likelihood of large ulcerating reactions in subjects in higher risk groups, we will first assess the safety of the injections in 80 subjects (20 from each group, except healthy non-contacts) before enrolling any additional subjects. Each individual will receive three 100µl injections (one of each high dose from each test antigen, and one of 2TU Tuberculin/PPD). The injections will be administered as previously described for Stage A, with one exception: the test sites will be at 1.5 inches, and 3.5 inches below the bend of the elbow on one arm and 3.5 inches below the bend of the elbow on the other arm, allowing a total of three injection sites. Each subject will be

randomly assigned one template for antigen administration (see Section 6, "Randomization/Enrollment/ Blinding Procedures"). Safety data will be collected and reviewed by the SMC, and their report will be forwarded to DMID, NHRC, and CSU IRB.

Stage C-1b: To further assess the reactivity of the skin test antigens, the low dose of each antigen will be administered to 80 subjects (20 from each group, except healthy non-contacts) exactly as stated in Stage C-1.

The skin test reactions (as compared to reactions from tuberculin/PPD, and Phase I studies of MLSA-LAM and MLCwA) will vary among the different groups participating in this study. It is expected that the BT/TT leprosy patients and healthy contacts of leprosy patients will have larger indurations at all or some *M. leprae*-derived test antigen sites (two dosages of MLCwA and two dosages of MLSA-LAM), and a variable reaction at the site of the tuberculin/PPD. In contrast, it is expected that the non-contacts, BL/LL leprosy cases, and tuberculosis patients will have smaller or no indurations at all leprosy test antigen sites. The tuberculosis patients will react with a large induration at the tuberculin/PPD site, and may react with an induration of less than 10 mm in diameter at the other four sites.

8.3 Follow-up Visits

8.3.1 Initial Follow-up

Follow-up will occur ~15 minutes; and ~30min (for stage C1b only) after antigen administration. Each skin test site will be examined by the administrator or other qualified staff member, for leakage, bleeding, or other local reactions (pain, erythematous, urticaria, and angioedema). In addition, subjects are asked if they are feeling faint, nauseated, or short of breath. Indication of symptoms will be recorded on the 15 Minute and 30 minute Reaction Record Form as "yes" or "no". If any of the symptoms are classified as an adverse event (see Section 11.2.1.2, "Management of Possible Adverse Events"), the Adverse Events Form must be completed. If the symptom is a Serious Adverse Event (see Section 11.2.1.3, "Management of Possible Serious Adverse Events"), the SAE Form must be completed as well. After the initial follow-up examination, it will be determined whether the subject is fit to be discharged. A "yes" or "no" will be marked on the 30 – Minute Reaction Record Form to indicate suitability for discharge. If the subject is not ready to be discharged, the AE Form and SAE Form (depending on severity of the event) must be completed. If the subject is ready to be discharged s/he will be given exact instructions regarding where and when to return for the next skin test reading, and will be given a contact phone number in case of emergency or if the subject cannot come to the test reading session.

Refer to Appendix A, Schedule of Procedures/Evaluations, for a detailed list of the sequence of events and evaluations that should occur during enrollment, administration of study product, and the follow-up.

8.3.2 Subsequent Follow-up

Subsequent follow-up visits to measure reactogenicity of skin test antigens and to monitor subject health will follow at 45-51h (Stage A only), 2-4 days, and 6-8 days (Stages B and C, only). Any subject with an induration of greater than 10 mm in diameter, observed during any of these readings, will be asked to return at 25-31 days for follow-up. Those subjects who need to return at day 25-31 will have their study/subject number posted on the bulletin board of the appropriate college/campus (primarily healthy non-contacts in all stages) and/or will be given instructions and a card indicating the date of their next visit (primarily patients and contacts of leprosy patients in Stage C) to serve as a reminder, without revealing the identity of the subject. Subjects experiencing any persistent reaction or adverse event will be followed until the resolution or stabilization of symptoms.

The method for measuring reactogenicity has been previously described in Section 4.5, “Methods for Collecting Data for Assessment of Study Objectives”. At each reading session, subjects will be interviewed and examined by an MO, nurse, or paramedic. Reactogenicity will be assessed, and measurements of induration (mm) and extent of erythema (Stage B, C-1and C-1b, only) will be recorded for each subject on the Skin Test Reading Form. A Follow-up Examination and Reaction Form will then be completed, similar to the 15 Minute Follow-up Form. The presence of symptoms including pain, erythema, bleeding, urticaria, infection, and blistering/ulcerations at each test site will be recorded as “yes” or “no”. A physical examination of the axillary lymph node will be performed and results (normal, painful, swollen, lymphangitis, or tender) will be indicated. If any of the symptoms are suspicious of an AE and/or SAE, the attending physician will examine the subject and complete the AE Form and/or SAE Form, if applicable. The subject’s axillary temperature will be taken using a thermometer placed under the arm pit and recorded. If the subject has a fever ($\geq$ 104 ºF) for more than 3 hours, both an AE and SAE Form must be completed. Upon completion of the follow-up examination, a “yes” or “no” will be marked on the Follow-Up Examination and Reaction Form to indicate subject suitability for discharge. Indications for discharge have been previously described (see Section 8.3.1, “Initial Follow-Up”).

In addition to interviewing and examining subjects for reactogenicity and adverse events, each subject in Stages A and B only will be asked to complete a Symptom Diary Card. Records of local and systemic reactions, including pain, swelling, redness, itching, blistering, or swollen lymph nodes, that occur on the day of the skin test and subsequent days following the skin test (three days for Stage A and seven days for Stage B), will be recorded. The subject will submit the Diary Card to the SC at the day 7 reading for review by the SC and/or the MO. Data from the Symptom Diary will not be entered into the study database, unless relevant information such as an AE is recorded. Any findings of relevance will be entered into the appropriate CRF (Follow-up Form, and/or AE Form).

The specific sequence of follow-up events includes:

- Day 0 (Stages A, B and C)
  - Screening
  - Enrolling
  - Blood sampling - Stage C only
  - Randomization
  - Study Product Administration Form
  - Complete the 15 minute follow-up
    - Interview
    - Examine skin test sites
  - Complete the 30 minute follow up (stage C1b only)
    - Interview
    - Examine skin test sites
    - Determine subject suitability to discharge
      - If not ready to discharge
        - Complete AE
        - Complete SAE, if needed
      - If subject is ready to discharge
        - Remind subject of next reading
        - Provide subjects (Stages A and B) with Symptom Diary Cards

- Day 2 is 48h ± 3h post antigen administration (Stage A, only)
  - Complete Skin Test Reading Form
    - Measure induration
  - Complete the Follow-Up Examination and Reaction Record
  - Interview subject
    - Examine skin test sites
    - Determine subject suitability to discharge
      - If not ready to discharge
        - Complete AE
        - Complete SAE, if needed
      - If subject is ready to discharge
        - Remind subject of next reading

- Day 3 ± 3h post antigen administration (Stage A and B) or ± 1 day post antigen administration (Stage C)
  - Review Symptom Diary Cards and collect - Stage A, only
  - Complete Skin Test Reading Form
    - Measure induration
    - Measure erythema (Stage B and C, only)
  - Complete the Follow-Up Examination and Reaction Record

        - Interview subject
        - Examine skin test sites
        - Determine subject suitability to discharge
            - If not ready to discharge
                - Complete AE
                - Complete SAE, if needed
            - If subject is ready to discharge
                - Remind subject of next reading

- Day 7 ± 1 day post antigen administration (Stages B and C)
    - Review Symptom Diary Cards and collect – Stage B, only
    - Complete Skin Test Reading Form
        - Measure induration
        - Measure erythema
    - Complete the Follow-Up Examination and Reaction Record
        - Interview subject
        - Examine skin test sites
        - Determine subject suitability to discharge
            - If not ready to discharge,
                - Complete AE
                - Complete SAE, if needed
            - If subject is ready to discharge
                - Remind subject of next reading, if applicable
                - Study Termination, if applicable

- Day 28 ± 3 days post antigen administration (Stage A, B and C – Subjects with an induration greater than 10mm at any reading)
    - Complete Skin Test Reading Form
        - Measure induration
        - Measure erythema
    - Complete the Follow-Up Examination and Reaction Record
        - Interview subject
        - Examine skin test sites
        - Determine subject suitability to discharge
            - If not ready to discharge
                - Complete AE
                - Complete SAE, if needed
            - If subject is ready to discharge
                - Study Termination

8.4 Final Study Visit

The final study visit will be at 69-75h (Stage A) or day 6-8 (Stage B and C), unless a positive reaction of greater than 10 mm in diameter is measured during this or any prior visit, or if an SAE or AE is on-going. In this event, the subject will be asked to return for a final visit on day 25-31 for examination and/or measurement of the skin test sites. Any reaction or event that persists will be followed-up until resolved or stabilized. Following the final subject interview and examination of skin test sites, the Termination Study Form will be completed, and the subject will be released from the study. At this time, in return for their time and inconvenience, subjects will receive a small gift valued at approximately US$11.

8.5 Early Termination Visit

Both the subject and the investigator have the authority to terminate an individual's participation in the study. The subject can voluntarily withdraw from this study at any time without penalty, as indicated in the Informed Consent Form. The investigator can elect to have a study participant removed from the study if warranted by an any immediate signs of hypersensitivity during the injection period, as indicated in the Manual of Operations (MOO) or if in the best interest for the subject. In addition, the sponsor of the study has the authority to terminate the entire study at any time.

If the subject has requested to withdraw voluntarily from the study, the MO/nurse/paramedic will ask the subject to continue scheduled follow-up visits, including the end-of-study visit. The final study visit will include a final measurement and examination of each skin test site, and a final interview of the subject. If a skin test reaction is greater than 10mm, the subject will be asked to return on day 25-31 for examination. Further follow-up will continue until the reaction has been resolved. The subject may or may not be willing to complete the scheduled study visits, and has the right to withdraw at any time.

On the other hand, if the subject is being discontinued from the study by the investigator due to an intervention caused by the study product, such as a serious adverse event that might make the subject uncomfortable or impede measurement of the skin test reaction, such as a local immediate hypersensitivity reaction, urticaria, or edema, s/he will be treated with the appropriate care under medical supervision and asked to return for follow-up visits until the symptoms of the event have resolved or the subject's condition becomes stable. At the point of resolving reactions greater than 10mm in diameter and/or mild to severe adverse events, a Study Termination Form will be completed by the attending staff member; specifically indicating the reason for termination.

9 STUDY INTERVENTION/INVESTIGATIONAL PRODUCT

9.1 Study Product Acquisition

9.1.1 Formulation, Packaging and Labeling

The "drug substances" to be tested are the soluble proteins of *M. leprae* (i.e., MLSA, *M. leprae* soluble antigen with minimal amounts of immunosuppressive glycans, most of which are LAM, called MLSA-LAM, and the cell wall-associated proteins of *M. leprae* called MLCwA. The active ingredients of these two intradermal skin test antigens are protein antigens of *M. leprae*. Human trials will be conducted with a single large batch of each antigen [MLSA-LAM (Batch 23) and MLCwA (Batch 23)] prepared in the Pilot Plant Facility at Colorado State University. Batch 23 was also used for Phase I testing.

Antigen MLSA-LAM is derived from soluble *M. leprae* extract following sonication and centrifugation at 27,000 x g then 100,000 x g, leaving the cytosol (MLSA). This soluble material is then extracted with detergent (Triton X-114) to reduce the amount of carbohydrate and lipid constituents (lipoglycans). MLSA-LAM contains the soluble protein antigens of *M. leprae*; over 100 individual proteins were initially recognized on 2-dimensional gels, and about 30 of these had been sequenced and the immunological responses studied in part (25). Foremost among these antigens are the 70 kDa (DnaK), 65 kDa (GroEL), 45 kDa, 38 kDa, 35 kDa major membrane protein (MMP-I), 22 kDa superoxide dismutase (SOD), 18 kDa small heat shock protein (SmHSP), 18 kDa bacterioferritin (Bfr), 10 kDa (GroES), and the ribosomal proteins S7/S12 (26-31). More recently, the full spectrum of proteins in soluble and insoluble subcellular fractions of *M. leprae* have been demonstrated and many more identified through the modern-day "proteomics" approach (32-34).

Antigen MLCwA is the 27,000 x g pellet extracted three times at 56°C with 2% sodium dodecylsulfate (SDS) followed by removal of SDS by column chromatography. MLCwA is further treated with Triton X-114 to reduce the amount of lipoglycans. MLCwA contains many of the same proteins as MLSA-LAM, particularly the 70 kDa and 65 kDa, the export/secretory proteins (notably the 30/31 kDa, multigene antigen 85 complex) (25), and also contains some larger, uncharacterized proteins. Details of the full spectrum of MLCwA constituent proteins have been published (32).

The structural formulae of only some of the protein-antigen constituents of these two formulations are known. In the two *M. leprae* extracts, there are hundreds of proteins, but the structural formulae of only about 70 are known, notably the 70 kDa/DnaK homolog, the two 18 kDa products, the 22 kDa, the 10 kDa/GroES homolog, a number of small proteins, several S and L subunit ribosomal proteins, the cysA/sulfate sulfurtransferase/rhodanese homolog, the Avi-3 homolog, etc. (25-34).

The pharmacological class of these two formulations is considered as intradermal skin test antigens. The dosage formulations of each skin test reagent are in concentrations of 1 μg/ml and 10 μg/ml and the route of injection will be intradermally in 0.1ml volumes. Antigens will be supplied in 2ml Wheaton vials closed with a 13mm rubber stopper and aluminum cap at appropriate concentrations. Each vial will contain 0.5 or 1.0 ml volumes, representing 5 and 10 doses per vial, respectively.

Original antigen labels contain the following information upon manufacturing: "Colorado State University" antigen name (MLSA-LAM or MLCwA), diluent (PBS/0.0005% Tween 80), concentration (1 and 10μg/ml), volume (0.5 or 1.0ml), "filtered and autoclaved", batch number, lot number, and the following statement, "Caution: New Drug – Limited by Federal Law to Investigational Use". Vials will be relabeled at the DMID-Clinical Agent Repository with a single letter code according to a randomized template provided by The EMMES Corporation. The identity of the antigen will be given to the SC in a sealed envelope for emergency unblinding purposes. Since there is not a recognized medical benefit of knowing the identity of an antigen in order to treat a subject for an adverse event, unblinding will not occur unless recommended by the SMC and approved by the DMID Project Officer.

9.1.2 Control Products: Saline and Tuberculin/PPD®

Two control products will be used in this study: (1) Saline, 0.9% NaCl, sterile, approved for human use (Abbott Laboratories Inc., Abbott Park, IL); and, (2) Tubersol®, 5TU dose (Aventis Pasteur Inc., Swiftwater, PA) in Stages A and B and Tuberculin/PPD RT 23 SSI, 2TU dose (Statens Serum Institute, Copenhagen, Denmark) in Stages C-1 and C-1b. Vials will contain 1 or 1.5ml total volume. Upon receipt by DMID, vials will be relabeled according to the randomized template described above (Section 9.1.1, "Formulation, Packaging, and Labeling").

9.2 Product Storage and Stability

While at Colorado State University the antigens, at 1μg/ml and 10μg/ml concentrations, are stored in a -70°C Revco freezer. There is a temperature log kept on the freezer with the temperature being recorded daily, excluding weekends and holidays.

Immediately prior to study initiation, the antigens will be packed in dry ice using an approved commercial carrier and shipped from Colorado State University to McKesson Bioservices (Rockville, MD) Repository. At McKesson Bioservices the antigens will be re-labeled as described in Section 9.1.1, "Formulation, Packaging and Labeling", and packed in a Styrofoam box containing ice packs for shipping to the Nepal test site. A temperature monitor ("temptails") will be used to monitor whether the vials were maintained at 2-8ºC. Once the antigens reach Nepal they will immediately be transported (still at 2-8°C) to the Research Laboratory at the AH in Kathmandu, Nepal. The vials will then be counted and a Drug Accountability Form completed, after which

they will be placed in a 4ºC refrigerator. This refrigerator will not be used for storage of any infectious material. The temperature of the refrigerator will be monitored daily for temperature stability (2-8°C). In the event of a power failure, the refrigerator will be run from a backup generator.

During the transport of the antigens to the clinical testing sites, they will be carried in a vaccine carrier (a Styrofoam box containing ice packs). Vials of leprosy skin test antigens, MLSA-LAM and MLCwA, and vials of saline planned for multidose use, do not contain preservative. Therefore, opened vials will not be reused more than 8 hours after opening. Unopened vials will be returned to AH and stored in the 4°C refrigerator for future use, only if temperature indicators demonstrated that the vials were maintained at the appropriate temperature (2-8°C) during transport.

At no time during the Phase II study will any vials be discarded. All unused vials will be kept at 4°C and all empty vials will be stored at 4°C, if space permits, or at room temperature in a box designated specifically for that purpose. Logs will be kept to account for all antigens received by the SC. After completion of the Phase II study, all unopened, partially used, and empty vials will be held until the visit of the site monitor, at which point they will be autoclaved and discarded.

Leprosy skin test antigens (MLSA-LAM and MLCwA), and concentrations (1 and 10µg/ml) have been tested for stability using the DTH guinea pig potency test. Each antigen at each concentration was tested every 3 months (0, 3, 6, and 12 months) after storage at three different temperatures (4, 37, and 56°C), and were found to be stable at each temperature and for the duration of the study as compared to their equivalent antigen and concentration stored at -70°C. Skin test antigens appear to be stable for a minimum of 1 year at all tested temperatures, and extended stability is probable, because the antigens are stable at accelerated temperatures. However, an expiration date and storage temperature of 1 year at 4°C has been assigned to both leprosy skin test antigens, because only a year of testing has occurred to-date. In addition, it is easier to maintain a constant temperature of 4°C across international borders, and the storage conditions are consistent with Tubersol® and Tuberculin/PPD RT 23 SSI. Effects of humidity have not been investigated.

Leprosy skin test antigen containers consist of Wheaton 2ml vials sealed with 13mm rubber septums and aluminum caps. To maintain security, a small aluminum seal must be broken on the cap to expose the rubber septum for dose extraction. Sterile saline is supplied in a sealed 2ml glass vial, and is treated in the same manner as the leprosy skin test antigen products, because it also lacks a preservative. Tubersol® is supplied in a sealed 1ml vial and Tuberculin/PPD RT 23 SSI is supplied in a sealed 1.5ml vial, and though they both contain a preservative, they will be handled in the same manner as the leprosy skin test antigens and saline to maintain blinding.

9.3 Training of Staff, Preparation, Dosage and Administration of Investigational Product

9.3.1 Training of Test Administration Staff

Four to six leprosy health workers from AH will be recruited to act as a pool of skin test administrators and readers (such a pool of individuals will allow

for staff holidays and possible staff illness). The staff selected will be licensed paramedics who are both qualified and experienced in the administration of injections and reading DTH skin test responses. An individual with extensive experience in administration of tuberculin/PPD and lepromin tests will act as the staff trainer.

Three staff training workshops will take place two months to two weeks prior to the administration of the skin test antigens to the first group of study subjects. The trainer will demonstrate proper technique for intradermal injection, and each trainee (upon signing a Practice Session Informed Consent Form) will have an opportunity during the workshop to give intradermal injections to each other using sterile saline. By this method, the trainees will be taught the standard techniques for skin test administration.

At the end of each training day, each skin test administrator will be given the opportunity to administer an actual tuberculin/PPD skin test with Tubersol® (Aventis Pasteur Inc., Swiftwater, PA) in training for Stages A, B and C-1 or RT 23 SSI (Statens Serum Institute, Copenhagen, Denmark) for Stage C-1b to at least three consenting volunteer leprosy health workers at Anandaban. The trainee's skin test administration technique will be observed and checked by the trainer using a procedural checklist. Seventy-two hours later, the trainees will record the readings from all of the volunteer leprosy health workers who had received tuberculin/PPD. Every trainee will read each reaction several times and the results will be recorded so that the reader's consistency can be assessed. The trainer will also read each of the volunteers' skin test reactions. Variation from the trainer's readings will be plotted on a standard correlation table, as recommended by the International Union against Tuberculosis and Lung Disease. The correlation chart will be used to identify systematic variations between the trainee's and the trainer's readings. Variations from the trainer's readings should not exceed 1 mm. Trainees whose skin test readings vary from the trainer's on average more than 2 mm will not be invited to participate in the study.

9.3.2 Preparation of Investigational Product

Antigens are supplied ready to use, and do not require any special thawing, diluting, mixing, or reconstitution preparation procedures. To ensure consistent handling procedures, vials will be inverted 3 times prior to each dose removal. The coded vials are kept at 4°C in a specifically designated refrigerator at AH. They will be transported to the test location at 2-8°C, in refrigerated vaccine carriers, and stored in these carriers until ready to use.

9.3.3 Dosage of Investigational Product

For Stage A and B, each individual will receive two injections of titrated doses of one of the two skin test antigens (in concentrations of 0.1μg and 1.0μg in 100μl sterile diluent, 0.9% NaCl, approved for human use, Abbott Laboratories), one injection of 100μl 5TU tuberculin/PPD Tubersol®; Aventis

Pasteur Inc., Swiftwater, PA, and one injection of 100µl diluent. For Stage C-1 and C-1b, each individual will receive each of the two skin test antigens at the high dose (1.0µg) only or low dose (0.1µg) only, respectively; and 2TU tuberculin/PPD, RT 23 SSI, Statens Serum Institute, Copenhagen, Denmark.

All injections will be in 0.1ml volumes from vials containing 0.5, 1.0 or 2.0 ml total. All injections will be made within 8 hours of the vials being opened. During this maximum 8 hour period cold storage (2-8ºC) will be maintained. The concentration ranges to be tested are based on results of our Phase I study (see p. 12), previous skin testing in humans with Rees' antigen (optimum dosage is 1.0µg in 100µl), as well as our skin test results in guinea pigs.

9.4 Accountability Procedures for the Investigational Product(s)

The status and location of all vials of antigen will be accounted for at all times. An accountability log will be maintained for each antigen. This will include details of the vial numbers dispensed on each study day, the dispensation of each vial, and the number of vials remaining in balance. After completion of use, vials will be stored until inspection at the end of the study. At the end of the study, all unopened, partially opened and empty vials will be autoclaved and discarded appropriately.

10 ASSESSMENT OF SCIENTIFIC OBJECTIVES (E.G., SAFETY OR SENSITIVITY/SPECIFICITY)

10.1 Methods and Timing for Assessing, Recording, and Analyzing Appropriate Outcome Measures

Methods used to assess safety and sensitivity/specificity of leprosy skin test antigens are listed below. Assessments will be performed on day 0, ~15 and ~30 (for stage C1b only) minutes following administration, 45-51h and 69-75h (Stage A), 69-75h and day 6-8 (Stage B) and day 2-4 and day 6-8 (Stage C). Any subject with an induration greater than 10 mm at any of the readings will be asked to return at 25-31 days for follow-up. Those subjects who need to return at day 25-31 will have their study/subject number posted on the bulletin board of the appropriate college/campus (primarily healthy non-contacts in all stages) and/or will be given instructions and a card indicating the date of their next visit (primarily patients and contacts of leprosy patients in Stage C) to serve as a reminder, without revealing the identity of the subject. Subjects experiencing any persistent reaction or adverse event will be followed until the resolution or stabilization of symptoms.

- Methods for assessing product safety:
  - Indication of local reactions, such as blistering, itching, pain, or bleeding
  - Measurement of systemic reactions, such as fever
  - Monitoring for AE and SAE

- Methods for assessing product sensitivity/specificity
  - Measurement of induration
  - Measurement of erythema

To remind the subject of their next scheduled visit and to facilitate drawing of proper case report forms, the subject is given a study card with their unique subject number recorded on it at the time of enrollment. This card will indicate for the subject the date and time of skin test administration, and the date of subsequent readings. In addition, it provides an emergency contact number for the study PI. If further readings are required, the dates will also be listed on this card.

Specific forms and logs have been developed for each Stage (A, B and C) and group [healthy unexposed (HU), BL/LL leprosy patients (LP), BT/TT leprosy patient (TD), healthy contacts of leprosy patients (HC), and tuberculosis patients (TB)] of the study. Subjects with leprosy disease classified as BL/LL or

LL are grouped into the BL/LL leprosy patient group, while those classified as BT/TT or TT are grouped into the BT/TT leprosy patient group. These case report forms (CRFs), allow subject information to be reported and tracked. They include:

- Case Report Forms
    - Screening
    - Demographic/Medical History
    - Eligibility Checklist
    - Antigen Administration Form
    - 15 – Minute Reaction Record
    - 30 – Minute Reaction Log (for C1b only)
    - Follow-up Examination and Reaction Form
    - Skin Test Reading Form
    - Study Termination Form
    - Serious Adverse Event Form
    - Adverse Event Form
    - Volunteer Symptom Diary Card
    - Laboratory Blood Test Results Form

- Logs
    - Case Report Form Log
    - Screening Log
    - Signature List Form (persons completing CRFs)
    - Index Case Code Number Log (healthy contacts)

Assessment of outcome measures for the safety of antigens MLSA-LAM and MLCwA at doses of 0.1µg and 1.0µg will be done throughout the study by continuously monitoring AE and SAEs, and at the end of each stage of the study by reviewing induration measurements, erythema, and adverse events. At the end of each stage, CRFs will be submitted for statistical analysis and compilation into a report by the DCC (The EMMES Corporation).

Stage A is essentially a safety review to allow DMID, NHRC, and CSU IRB to assess safety data before the injections are given to a larger group of healthy non-exposed subjects in stage B. We have elected to start with non-contacts whom we expect to have small or negative reactions and so minimize the probability of adverse responses. Inclusion of 100 total subjects in stages A and B is sufficient to estimate the frequency of adverse events from the administration of these new skin test reagents.

Assessment of outcome measures for the sensitivity/specificity of antigens MLSA-LAM and MLCwA at doses of 0.1µg and 1.0µg will be done at the end of Stage C, by reviewing induration and erythema from leprosy and TB patients, leprosy patient contacts and healthy non-contacts who have volunteered for this study. The skin test antigen sites of all 260 individuals in this Phase II study (10 from Stage A, 90 from Stage B, and 160 from Stage C) will be evaluated by the same methods used to determine the response to tuberculin/PPD, i.e., evaluation of induration and erythema. Comparison of skin test reactions to leprosy antigens in diagnosed leprosy patients versus leprosy

patient contacts or non-exposed healthy volunteers or TB patients will inform sensitivity and specificity estimates.

The results from the 100 healthy non-contacts studied in Stages A and B will be compared to the results from leprosy patients, contacts of leprosy patients, and tuberculosis patients in Stage C. The source data document for the study will be the case report forms generated during the study.

Of those with leprosy and tuberculosis, a combination of treated and partially treated cases will be tested. Tuberculosis patients will be those who have completed the intensive phase of short-course chemotherapy and are currently receiving anti-tuberculosis medicines. The type and length of treatment completed at the time of skin testing will be recorded. The length of therapy in the case of leprosy patients may be a factor by which we categorize the skin test results in subsequent analysis.

Data collected from each leprosy and tuberculosis patient will include the extent of disease as defined by the Ridley-Jopling scale for leprosy (35), and defined as pulmonary sputum smear positive, pulmonary sputum smear negative, or non-pulmonary for tuberculosis patients (36), and the length and type of treatment. This information will be included in the CRFs. BCG vaccination is practiced in Nepal, but implementation is relatively recent and coverage by no means complete; 30% to 50% of subjects aged less than 20 have been vaccinated and the proportions will be lower for older age groups. We will record the presence and size of the BCG vaccination scar in all subjects, and this information will be used to categorize results in the subsequent analysis.

We will conduct tuberculin/PPD testing on all patients in Stages A, B, and C. We believe it is important to have a contemporaneous tuberculin/PPD test with which to compare the reactions to the leprosy skin tests to assess specificity of the leprosy skin test antigens. Tuberculin/PPD reactivity will be a measure of not only BCG vaccination, but also tuberculosis exposure, as well as exposure to non-pathogenic environmental mycobacteria, to some extent (24). Thus, in all subjects, we will be comparing diameters of the reactions to PPD with those to the new leprosy antigens.

For healthy contacts of leprosy patients, we will record the type of contact (household or professional), and length of contact, and use this information to analyze the results. In addition, all subjects will be stratified by age and gender. Analysis of the outcome measures for safety and sensitivity/specificity, in conjunction with demographic and historical information gained from the subject, examinations, and patient medical records will allow stratification of data to meet both primary and secondary objectives.

10.2 Modification and Discontinuation of Study Intervention/Investigational Product for a Participant

10.2.1 Dose/Schedule Modifications for a Subject

Selection of a safe dosage of the test antigens to use in future studies will be based on the distribution of skin test reaction data obtained from healthy

subjects in Stages A, B, C-1 and C-1b of the study. If any of the subjects in stage A, B, or C-1 show ulcerations at the 1.0μg dose of MLCwA or 1.0μg dose of MLSA-LAM test sites, then only the 0.1μg dosage of the skin test antigen causing ulceration will be considered for future studies.

Additional adverse events may prompt a dose modification based on recommendations from the SMC.

10.2.2 Criteria for Discontinuation of Study Intervention/Product for Withdrawal of a Subject (or a Cohort)

Reasons to stop the study will include, but not necessarily be limited to, any anaphylactic reaction to one or both of the new skin test antigens. Severe ulceration and/or necrosis or urticaria at the skin test antigen sites would also be reasons for stopping the study temporarily for review and advice before potentially proceeding. Based on reviews of an SAE associated with the study product, the SMC can recommend termination of the study to the DMID. The DMID, NHRC and CSU IRB have the authority to terminate the study at their own discretion.

Reasons for discontinuing a study subject prior to intervention of product would include:

- Development of an illness, other medical condition, or situation indicating to the investigator that continued participation in the study, i.e., product administration would not be in the best interest of the subject.
- Development of any exclusion criteria (Section 5.3, "Subject Exclusion Criteria") prior to product administration may be cause for discontinuation.

If the study product is discontinued following product intervention, subjects experiencing any persistent reactions or adverse events will continue to be followed until the resolution or stabilization of symptoms. Removal of a study subject will not prompt the need to enroll further subjects unless greater than 10% are removed and/or lost to follow-up. The statistical justification for subject numbers indicates that the study will be able to meet the primary statistical objectives, even should up to 10% of the subjects withdraw and/or be lost to follow-up.

11 ASSESSMENT OF SAFETY

Safety outcome measures are listed in Section 10.1, “Methods and Timing for Assessing, Recording, and Analyzing Appropriate Outcome Measures”. Specifications of safety outcomes are described here.

11.1 Specification of Safety Parameters

Safety is a primary study outcome measure. Specifications of safety parameters are listed in detail below.

- To evaluate the safety of these skin test antigens in healthy non-contacts, with a particular interest in assessing whether, and to what degree, individuals are sensitized to the antigens (study Stages A and B)
- To evaluate the safety of these skin test antigens in leprosy patients and contacts of leprosy patients (study Stage C)

11.2 Methods and Timing for Assessing, Recording, and Analyzing Safety Parameters

Refer to Section 2.1.2, “Leprosy Skin Test Antigens, 1919-today”, for relevant information and references on the history and safety of prior leprosy skin test antigens. The primary source of risk information for this investigational product is the Investigator’s Brochure.

11.2.1 Adverse Events, Reactogenicity and Serious Adverse Events

The Principal Investigator is responsible for reporting all AEs that are observed or reported during the study, regardless of their relationship to study product.

11.2.1.1 Definitions

11.2.1.1.1 Adverse Event (AE)

An AE is any untoward medical occurrence in a patient or clinical investigation subject to whom a pharmaceutical product has been administered, and that does not necessarily have a causal relationship with this treatment. An AE can therefore be

any unfavorable and unintended sign (including an abnormal laboratory finding), symptom, or disease temporally associated with the use of a medicinal (investigational) product, whether or not in association to the medicinal (investigational) product, within a specified period of time. The occurrence of an adverse event may come to the attention of study personnel during study visits and interviews or by a study recipient presenting for medical care.

All adverse events must be graded for intensity and relationship to study product.

11.2.1.1.2 Serious Adverse Event (SAE)

An SAE is any untoward medical occurrence within a specified period of time that at any dose:

- Results in death.
- Is life-threatening. Any adverse therapy experience that places the subject, in the view of the investigator, at immediate risk of death from the reaction as it occurred (i.e., it does not include a reaction that, had it occurred in a more serious form, might have caused death).
- Requires in-patient hospitalization or prolongation of existing hospitalization.
- Results in persistent or significant disability or incapacity.
- Is a congenital anomaly/birth defect.
- An event that required intervention to prevent permanent impairment or damage.

Important medical events that do not result in death, are not life-threatening, or do not require hospitalization may be considered serious adverse events when, based upon appropriate medical judgment, they might jeopardize the subject and might require medical or surgical intervention to prevent one of the outcomes listed above.

11.2.1.1.3 Reactogenicity

Reactogenicity events are adverse events that are common and reasonably expected for the intervention/ investigational product being studied. Reactogenicity events will be collected at the time of injection site assessment, observed during study visits.

11.2.2 Management of Possible Adverse Events

Adverse events will be managed according to standard care of the study medical staff or primary care physician.

11.2.3 Management of Possible Serious Adverse Events

Serious adverse events will be managed according to standard care of the study medical staff or primary care physician.

11.3 Reporting Procedures

11.3.1 Adverse Events

Adverse Events including local and systemic reactions not meeting the criteria for "serious adverse events" should be captured on the appropriate case report form. Information to be collected includes event description, time of onset, investigator assessment of severity, relationship to study product, time of resolution of the event, seriousness, and outcome. All adverse events occurring while on study must be documented appropriately regardless of relationship. All AEs will be followed to stabilization, if they do not resolve. An event is reportable as associated with the product if the possibility of a relationship of the product to the event cannot be ruled out.

Any medical condition that is present at the time that the subject is screened should be considered as baseline and not reported as an AE. However, if it deteriorates at any time during the study it should be recorded as an AE.

11.3.2 Serious Adverse Events

All serious adverse events that occur during the study will be:

- Recorded on the appropriate serious adverse event case report form
- Followed through resolution by a study physician
- Reviewed by a study physician

Any AE considered serious by the Principal Investigator or Sub investigator, or which meets the aforementioned criteria must be submitted on an SAE form to PPD Development Inc., DMID's pharmacovigilance contractor, at the following address:

Medical Affairs/Pharmacovigilance
PPD Development
3151 17th St.
Wilmington, NC 28412
SAE Fax line: 888 488-9697
SAE Fax line Non North America: 011-910-772-7069

Questions about SAE reporting can be referred to the SAE Hotline (available 24 hours a day/7 days a week) at 800 201-8725.

The study clinician will complete a Serious Adverse Event Form within the following timelines:

- All deaths and life threatening events, whether associated or not associated, will be recorded on the Serious Event Form and sent by fax within 24 hours of site awareness of the death.
- Serious adverse events other than death or life threatening events, regardless of relationship, will be reported via fax by the site within 72 hours of becoming aware of the event.

Other supporting documentation of the event may be requested by the pharmacovigilance contractor and should be provided as soon as possible.

All SAEs will be followed until satisfactory resolution or until the Principal Investigator or Sub investigator deems the event to be chronic or the patient to be stable.

All SAEs will be reported as required to each sites' IRB. SAE data will be entered into the electronic data system provided by the EMMES Corporation.

Protocol deviations will be documented. Protocol deviation data will be entered into the electronic data system provided by the EMMES Corporation.

Dr. Bharat Kumar Yadav of Patan Hospital, located in Kathmandu, Nepal will be the ISM for the study. His contact information is provided below:

Dr. Bharat Kumar Yadav, MD
Patan Hospital
P.O. Box 252
Lagankhel, Lalitpur, Nepal
Tel #: 011-977 (1) 522-2266 (Patan Hospital)
011-977 (1) 553-0003 (Patan Hosptial Quarters)
977-1-557-2921 (home)
Fax #: 011-977 (1) 522-5559
email: patan@hospital.wlink.com.np
yadav100@hotmail.com

Dr. Jonathan Rose will be the DMID Medical Monitor for the study. Her contact information is provided below:

Jonathon Rose, MD, MPH, DABPM
Medical Officer
Office of Clinical Research Affairs
Division of Microbiology and Infectious Diseases
National Institute of Allergy and Infectious Diseases
National Institutes of Health
6610 Rockledge Drive, Room 6057, MSC 6603
Bethesda, Maryland 20892-6603
P: 301-443-7707
F: 301-480-0728
E-mail: rosejon@niaid.nih.gov

Intensity of Event: All adverse events will be assessed by the investigator using a protocol defined grading system (see Section 7.1.5, "Reactogenicity Assessments").

For events not included in the protocol defined grading system, the following guidelines will be used to quantify intensity.

- Mild: events require minimal or no treatment and do not interfere with the subject's daily activities.
- Moderate: events result in a low level of inconvenience or concern with the therapeutic measures. Moderate events may cause some interference with functioning.
- Severe: events interrupt a subject's usual daily activity and may require systemic drug therapy or other treatment. Severe events are usually incapacitating.
- Life threatening: Any adverse drug experience that places the patient or subject, in the view of the investigator, at immediate risk of death from the reaction as it occurred, i.e., it does not include a reaction that had it occurred in a more severe form, might have caused death.

Adverse events characterized as intermittent require documentation of onset and duration of each episode.

Relationship to study products: The investigator's assessment of an AE's relationship to the study drug is part of the documentation process, but it is not a factor in determining what is or is not reported in the study. If there is any doubt as to whether a clinical observation is an AE, the event should be reported. All adverse events must have their relationship to study product assessed using the following terms: associated or not associated. In a clinical

trial the study product must always be suspect. To help assess, the following guidelines are used.

- Associated – There is a known temporal relationship *and/or*, if re-challenge is done, the event abates with de-challenge and re-appears with re-challenge *and/or* the event is known to occur in association with study product or with a product in a similar class of study products. An event is reportable as associated with the study product if the possibility of a relationship of the study product to the event cannot be ruled out.

Not Associated -the AE is completely independent of study product administration; and/or evidence exists that the event is definitely related to another etiology.

11.3.3 Reporting of Pregnancy

Pregnancy tests will be performed during study screening. Pregnancies that are reported by the subject during study participation will be reported as adverse events and the volunteer will be referred to their physician following standard referral procedures.

11.4 Halting Rules

Safety findings that would temporarily suspend enrollment and/or study intervention until an *ad hoc* safety review meeting is convened by phone or e-mail with the DMID PO, the ISM and/or the SMC would include:

- Any SAE deemed associated to the skin test antigens
- AEs occurring that, in the opinion of the investigator and ISM may warrant review by the SMC
- Any death, whether related or not

The DMID PO, with input from the ISM and/or the SMC, will determine whether the study should continue per protocol, proceed with caution, be further investigated, be discontinued, or be modified and then continued. Suspension of enrollment (for a particular group or for the entire study) is another potential outcome of these recommendations. Recommendations by the DMID PO will then be submitted to DMID, NHRC, CSU IRB, and the FDA.

***Ad Hoc* Meetings of SMC:** The SMC may convene an *ad hoc* meeting to discuss any issue of safety raised by an investigator, the sponsor, or a member of the SMC. At the discretion of the investigators, the sponsors, and SMC members, a non-serious adverse event that is either associated with the product, or does not meet the stopping rules criteria may be considered as a trigger for an *ad hoc* SMC meeting to assess the safety of the product, without resulting in halting the enrollment of the trial.

12 CLINICAL MONITORING STRUCTURE

Site Monitoring will be conducted to ensure that human subject protection, study procedures, laboratory procedures, study intervention administration, and data collection processes are of high quality and meet sponsor, CGP/ICH, and regulatory guidelines, and that the study is conducted in accordance with the protocol and sponsor SOPs. DMID, the sponsoring agency, or its designee will conduct site monitoring visits either on-site or off-site as detailed in the monitoring plan or in the Manual of Operations.

13 STATISTICAL CONSIDERATIONS

13.1 Overview and Study Objectives

13.1.1 Scientific Rationale

The overall objective is to evaluate two new leprosy skin test antigens as diagnostic/epidemiological tools designed to measure the extent of leprosy infection in individuals and communities living in leprosy endemic areas.

13.1.2 Objectives

Stage A

- Primary objective:
  To evaluate the safety of the two leprosy skin test antigens in a small number of healthy individuals without known exposure to clinical leprosy or tuberculosis, living in a region endemic for leprosy.

Stage B

- Primary objective:
  To evaluate the safety of the two leprosy skin test antigens in larger numbers of healthy individuals without known exposure to clinical leprosy or tuberculosis, living in a region endemic for leprosy.

- Secondary objectives:
  - To compare responses to the leprosy skin test antigens to those of PPD/tuberculin, in order to determine if there is differentiation between the two in this population with presumed exposure to indigenous *Mycobacterium tuberculosis,* other indigenous mycobacteria, and in some cases, subjected to BCG vaccination.
  - To combine data on skin test responses to the leprosy skin test antigens and to PPD/tuberculin, with data from an equivalent group in Stage C, to arrive at a valid definition of a positive response, versus a negative response (i.e. “cut-off” point).

Stage C

Stage C-1 and C-1b

- Primary objective:
  To evaluate the safety of the two leprosy skin test antigens in small numbers of healthy contacts of leprosy patients, BT/TT leprosy patients, BL/LL leprosy patients, and TB patients.

- Secondary objectives:
  - To evaluate responses to the two leprosy skin test antigens and to PPD/tuberculin in healthy contacts of leprosy patients, BT/TT leprosy patients, BL/LL leprosy patients, and tuberculosis patients to determine if these leprosy antigens show promise for the selective diagnosis of leprosy infection.
  - To apply a whole blood assay for IFN-γ response to these antigens, and an antibody response to the leprosy specific PGL-I antigen, to these population groups, in order to provide preliminary indications as to *in vivo* skin testing being replaced or augmented with blood assays.

13.2 Study Population

All subjects for Stages A, B, and C will be Nepali nationals (including expatriates from India), between the ages of 18 and 60 years, weighing greater than 30 Kg (female) or 38 Kg (male). Healthy, non-leprosy exposed subjects and tuberculosis patients will serve as the negative control group. Healthy leprosy exposed contacts and BT/TT leprosy patients will serve as positive control groups, and BL/LL leprosy patients will serve as concurrent controls.

The skin test reactions will vary among the groups participating in this study. It is expected that the non-contacts and BL/LL leprosy cases along with the tuberculosis patients will have smaller or negative indurations at all leprosy skin test sites. The tuberculosis patients will react with a large induration at the PPD/tuberculin site, and may react with an induration less than 10 mm at the other sites. Healthy contacts of leprosy patients and BT/TT leprosy patients are expected to have larger indurations at *M. leprae*-derived antigen sites, and a variable reaction at the site of the PPD/Tuberculin. Data collected from skin test reactions from these five groups will allow study objectives to be met.

The rationale for choosing these study groups is as follows. Healthy subjects not exposed to leprosy will be considered endemic area controls, which are necessary to monitor exposure to environmental mycobacteria and/or exposure to tuberculosis. This group of volunteers will be recruited to establish the safety of both antigens and antigen dosages, as well as to establish a baseline response in a leprosy endemic area. Tuberculosis patients will serve as controls recruited to aid in establishing the specificity of leprosy skin test antigens and to show PPD positivity. The healthy contact group (including family members of leprosy patients) will serve as a positive response group. That is, in comparison to the proportion of healthy non-contacts with a positive reaction to the test antigens, we expect that at least twice as many healthy contacts of leprosy patients will have a positive test, due to the fact that the contacts have been exposed to leprosy and probably have developed at least a small immunological reaction to *M. leprae*. On the other hand, the leprosy patient study groups (BT/TT and BL/LL) fall into two categories at opposite ends of the disease spectrum. Historically, BT/TT leprosy patients produce a strong skin test response against leprosy antigens, because of their strong T-cell response to *M. leprae*. In comparison, BL/LL leprosy patients produce a weak or negligible response due to an anergic T-cell response to leprosy skin test

antigens. Alternatively, the B-cell immunological response is weak in BT/TT leprosy patients and strong in BL/LL leprosy patients. Data collected from each group will be analyzed by comparing the mean skin test response against leprosy skin test antigens as compared to PPD, thus enabling questions of safety and specificity to be addressed.

Ethical concerns have been taken into account for each group of volunteers. It is expected that healthy non-contacts, tuberculosis patients, and some BL/LL patients may have small (less than 10mm) induration reactions to the leprosy skin test antigens, due to cross-reactivity with *M. tuberculosis*, BCG vaccination, and/or environmental mycobacteria. Known environmental mycobacteria present in soil and water include: *M. avium-intracellulare, M. kansasii, M. xenopi, M. ulcerans, M. marinum, M. malmoense, M. fortuitum*, and *M. chelonei*. Tuberculosis patients are expected to produce a strong induration (10-30mm) response to PPD, however, the reaction is not expected to develop into an AE based on previous PPD testing in this region. Since tuberculosis is prevalent in Nepal and many individuals were vaccinated with BCG, most subjects will have been exposed to tuberculosis antigens, and therefore the remaining groups (healthy non-contacts, BL/LL and BT/TT leprosy patients, and contacts of leprosy patients) may react to PPD. Hence, PPD testing will be a measure of not only exposure to tuberculosis, but also BCG vaccination, and/or non-pathogenic environmental mycobacteria, to some extent. Finally, BT/TT leprosy patients and healthy contacts of leprosy patients are expected to respond more strongly (greater than 10mm induration) to leprosy skin test antigens. In order to minimize the risk to these groups, a preliminary test of each antigen at the high dose (1.0μg/100μl) will be tested in a small group of 20 subjects from each group, except the healthy non-contacts (Stage C-1). If serious adverse events are indicated, the causative antigen or antigen dose will be dropped from the study, following a safety review. To provide a comparative population at the low dose (0.1μg/100μl), Stage C-1b will be performed. Overall, the anticipated risks are the same as those encountered with other intradermal antigens such as tuberculin (see Section 2.3.1.1, "Skin Testing Risks").

13.3 Study Design

This is a prospective study where the participants all receive the same set of antigens, but are randomized to receive them in different patterns, that is, according to different "templates". The participants do not know which template is used and the readers, or evaluators are also blinded to the pattern of antigen delivery. Stages A and B randomized participants to only a single study antigen, at two doses for each volunteer, plus PPD and saline. Schemas for stages A plus B and C follow.

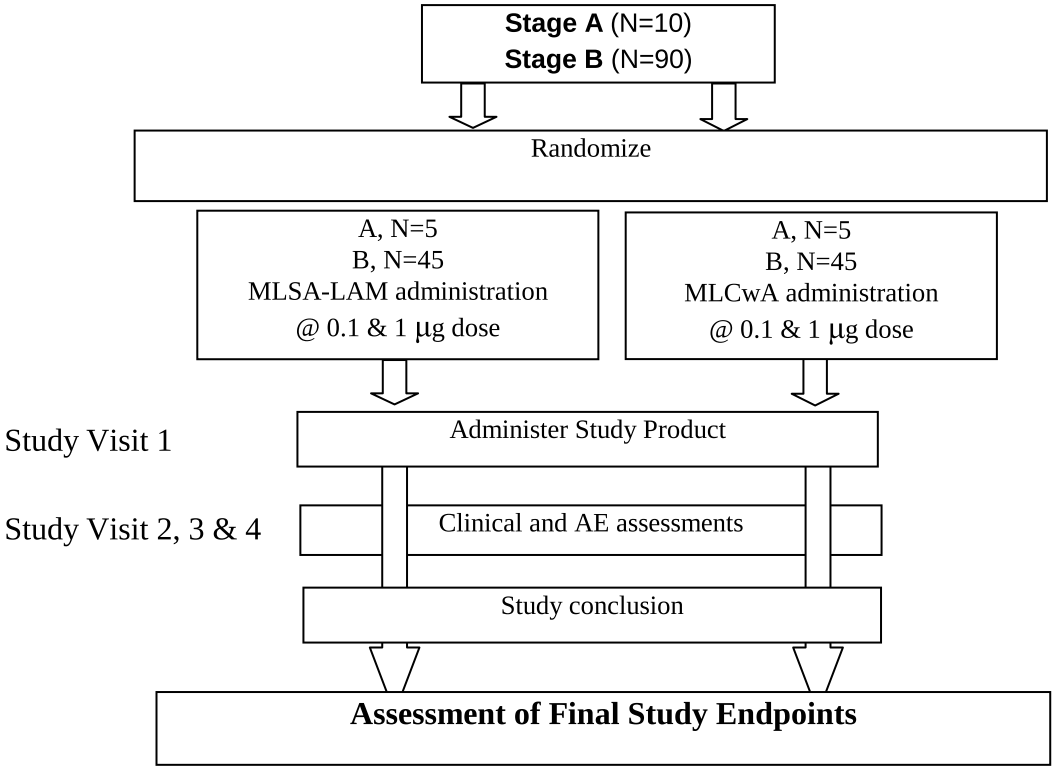

Volunteers in stage C-1 will receive both leprosy skin test antigens at the high dose of 1µg per 100µl and PPD at 2TU, while volunteers from stage C-1b will receive the same antigens at the low dose of 0.1µg per 100µl and PPD at 2TU. Since three test sites are required as compared to four in Stage A and B, they will be reassigned (2 sites on the first arm and one on the second arm), and administration templates will be randomized to different administration patterns. For Stage C-1 and C-1b, there will be 20 subjects from each study group, except healthy non-contacts (contacts of leprosy patients; BT/TT and BL/LL leprosy patients; and, tuberculosis patients).

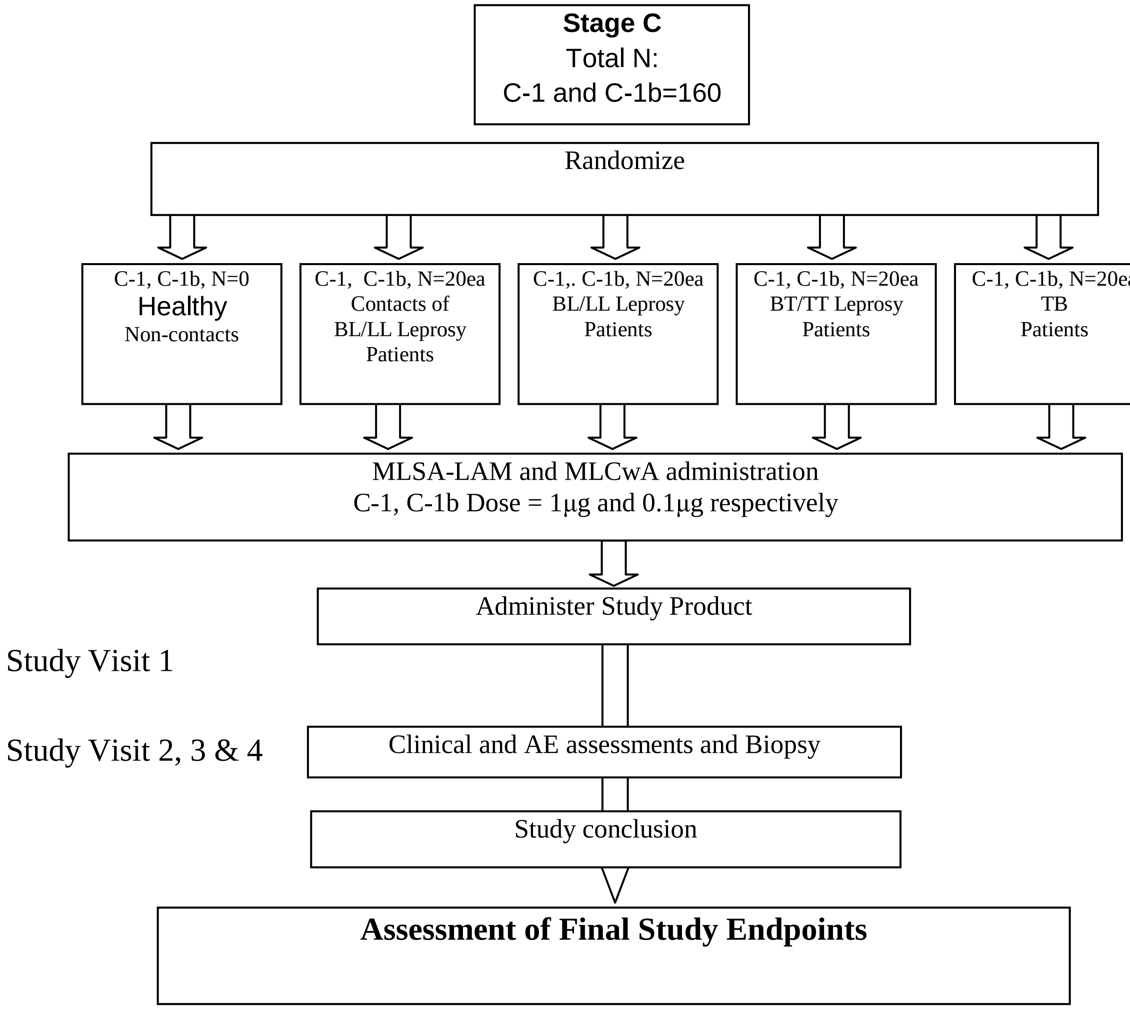

13.4 Study Outcome Measures

The primary outcome measure of this study is induration resulting from the skin test antigen DTH response. The method for measuring induration has been described in Section 4.5, "Methods for Collecting Data for Assessment of Study Objectives". As mentioned previously, evaluation of this observation variable will be performed using the same techniques used to evaluate the tuberculin/PPD response. A tuberculin reaction greater than 10mm of induration is classified as positive for persons who are not immunocompromised or previously in contact with TB patients, but have other risk factors for TB, such as living in a country where TB is prevalent. Induration measurements will be taken on days 2 and 3 (Stage A) and days 3 and 7 (Stage B and C) after skin test administration, at each test antigen site. If a subject is observed to have an induration greater than 10 mm at any injection site, at either study visit, they will

be asked to return at 25-31 days for a final induration measurement. Any reaction that persists will be followed-up until resolved or stabilized.

13.5 Study Hypotheses

This study is not designed to test a formal hypothesis such as the superiority or non-inferiority of different interventions. Rather, it is designed to describe the safety and primary response measure of induration resulting from skin test antigen DTH responses in selected groups all living in the same environment where leprosy is endemic.

An informal statement of the safety hypothesis for this study is that the study antigens are safe in all five-study groups. The study hypothesis for the diagnostic DTH response is that induration responses will be distributed in an approximately bi-modal fashion such that the healthy contacts of leprosy patients and BT/TT leprosy patients have generally larger responses in comparison to the other groups. If such a bi-modal distribution is observed for either antigen, it will be used to define positive responses, response rates for the different groups, sensitivity and specificity of the response, and the performance of the two antigens will be compared.

13.6 Sample Size Considerations

The primary variable outcome measure used for all calculations will be the mean induration measurement obtained from two independent readers for each skin test site. Our experience in this region of Nepal suggests that no more than 10% of subjects will be lost to follow-up. We are not planning to administer skin tests to additional subjects should some of the subjects not return for the 45-51 hour or later readings.

13.6.1 Safety

Stage C-1 and C1b are intended to provide safety information on small numbers of participants (N=20 for each stage) within each of four different groups that theoretically may be at higher risk of serious adverse responses to the novel antigens. The table below shows the probability of observing 1 or more serious adverse events up to 5 or more serious adverse events, related to antigen administration. This probability is calculated from a sample of 18 subjects (20 subjects minus the possible loss of 10%) for a range of assumed “true” but unknown probabilities of a serious adverse event. Based on these results, if the true serious adverse event rate is 10% then there is an 85% chance of observing one or more serious adverse events in any one of the four groups, even if there is a 10% loss to follow-up within groups. Additional calculations, not shown, demonstrate that when there is no loss to follow-up the chance of observing at least one event increases to 88%. Aggregating the four groups and assuming 10% loss (n=72) there would still be an 89% probability of observing at least one event assuming that the groups in aggregate had a true event rate as low as 3%.

Probability of Observing Adverse Events Within Any Study Group in Parts C-1 and C-1b Given a Range of True but Unknown Event Probabilities

Sample Size of 18 (assuming 2 lost to follow-up)

"True" Unknown Population Probability of an Event	Probability of Observing 1 or more Events	Probability of Observing 2 or more Events	Probability of Observing 3 or more Events	Probability of Observing 4 or more Events	Probability of Observing 5 or more Events
-0.10%	1.78%	0.02%	0.00%	0.00%	0.00%
0.50%	8.63%	0.36%	0.01%	0.00%	0.00%
1.00%	16.55%	1.38%	0.07%	0.00%	0.00%
2.00%	30.49%	4.95%	0.52%	0.04%	0.00%
3.00%	42.20%	10.03%	1.57%	0.18%	0.02%
4.00%	52.04%	16.07%	3.33%	0.50%	0.06%
5.00%	60.28%	22.65%	5.81%	1.09%	0.15%
10.00%	84.99%	54.97%	26.62%	9.82%	2.82%
15.00%	94.64%	77.59%	52.03%	27.98%	12.06%
20.00%	98.20%	90.09%	72.87%	49.90%	28.36%
25.00%	99.44%	96.05%	86.47%	69.43%	48.13%

As the above table shows, the probability of observing events of a given underlying ‘true’ probability is identically in C-1 and C-1b since the sample sizes of the two parts are the same. If the ‘true’ probability of an event is similar in both groups, then combining parts C-1 and C-1b may provide further precision in the estimation of the probability of observing the indicated number of events. In particular, if the true serious adverse event rate is 10% then there is more than a 97% chance of observing one or more serious adverse events in any one of the four groups, even if there is a 10% loss to follow-up within groups.

Probability of Observing Adverse Events Within Any Study Group when Combining Parts C-1 and C-1b Given a Range of True but Unknown Event Probabilities

Sample Size of 36 (assuming 4 lost to follow-up)

"True" Unknown Population Probability of an Event	Probability of Observing 1 or more Events	Probability of Observing 2 or more Events	Probability of Observing 3 or more Events	Probability of Observing 4 or more Events	Probability of Observing 5 or more Events
0.10%	3.54%	0.06%	0.00%	0.00%	0.00%
0.50%	16.51%	1.41%	0.08%	0.00%	0.00%
1%	30.36%	5.03%	0.56%	0.05%	0.00%
2%	51.68%	16.18%	3.50%	0.57%	0.07%
3%	66.60%	29.41%	9.28%	2.22%	0.42%
4%	77.00%	42.50%	17.34%	5.46%	1.37%
5%	84.22%	54.33%	26.79%	10.37%	3.24%
10%	97.75%	88.74%	71.21%	49.15%	28.92%
15%	99.71%	97.88%	92.24%	80.94%	64.50%
20%	99.97%	99.68%	98.40%	94.78%	87.31%
25%	100.00%	99.96%	99.74%	98.90%	96.58%

As described in the above table, Stage C-1b provides increased probability of detecting safety concerns. In addition, combining BT/TT and BL/LL leprosy groups and the contact groups from C-1 and C-1b provides a cumulative sample of 108 assuming a 10% loss. Considering this combined group, there will be better than 91% probability of observing at least one event with a true incidence rate of the event is 2% or higher.

13.6.2 Leprosy Detection/Diagnosis

The utility of a medical diagnostic test is often expressed in terms of the receiver operating characteristic (ROC) for the test (37). The ROC is a plot of the relationship between the false positive rate (FPR), or 1-specificity, and the true positive rate (TPR), or sensitivity. Given a pair of individuals, one with and one without the medical condition of interest, the area under the ROC curve (AUC) is equal to the probability that the test will correctly discriminate between this pair. This study will be the first one using these test antigens in volunteers with the condition of interest making it difficult to establish a hypothesis concerning the AUC.

The study design provides for a variety of possible subset analyses for a given dose of each antigen. Nine combinations of volunteers expected to be non-reactive and reactive respectively are listed in the following table. This is not a definitive Phase III trial, although it has some characteristics in that specificity/sensitivity are considered in the analysis, thus adjustments for multiple comparisons are not considered here. The multiple comparison possibility will be considered at the time of actual data analysis.

Although the observational unit in this study is an individual injection site, the experimental unit is subject. Since subjects in C-1 will receive the higher dose of antigen and C-1b subjects the lower dose, results from C-1 and C-1b will not be combined to construct the ROC curve or to estimate the sensitivity/specificity of the antigens. However, independently, C-1 or C-1b will be used construct the ROC curve and estimate the sensitivity and specificity for each dose of the antigen separately. The estimates and sample sizes in the remainder of this section reflect this.

Comparison Groups of Interest

Assuming Uniform Loss to Follow-up of 10% in each Group

Groups of Interest	BT/TT Patients	Contacts of BL/LL Patients	Contacts of BL/LL Plus BT/TT Patients
BL/LL Patients	36 and 36	36 and 36	36 and 72
BL/LL Patients Plus TB	72 and 36	72 and 36	72 and 72

Using a two tail-test with Type I error equal to 5% and Power equal to 80% the proposed analyses can detect a difference in area of 0.186 or less. This difference occurs for the worse case, AUC0'=0.5 and the smallest sample size combination (BL/LL and BT/TT subjects, N=36 and 36 respectively). Using the largest proposed sample (N=72 and 72 for expected negative and positive responders, respectively) and a more optimist null hypothesis of AUC0'=0.75 results in a detectable difference of 0.101.

All of these analyses may be performed for both antigens, MLSA-LAM and MLCwA at both doses. Moreover, the ROC curves can be compared. The largest proposed sample size (N=72 and 72 for expected negative and positive responders) is expected to be sufficient to detect a difference in the AUC in the range of 0.135 to 0.100.

This calculation is not setting a null hypothesis (AUC0') for the basis of a successful study or designed to definitively test the difference in the two antigens. These values are provided however, to give an indication of the power inherent in the proposed sample size.

ROC analyses involve both the expected positive and negative responding groups. Sensitivity and specificity analyses are restricted to each of these groups individually. The following table provides an indication of the precision obtainable in measuring sensitivity and specificity in this study, as determined by the half width of the confidence interval for some hypothetical results and appropriate sample sizes, as described above. Precision decreases as the observed proportion approaches 0.5 and as the sample size is decreased. Therefore, the worse case scenarios for sensitivity analyses would be restricting to BT/TT patients (N=36) and observing a 50% sensitivity. In that case, the precision would be 0.16 or a confidence interval of (0.34, 0.66). At the other extreme, if all contacts of BL/LL plus BT/TT (N=72) patients are included in the

analyses and a 0.95 sensitivity is observed, the precision is 0.05, or a confidence interval of (0.90, 1.00).

Precision of Observed Sensitivity

Sample Size	Observed Sensitivity/ Specificity	Precision based on Half-Width of 95% CI
36	0.5	0.16
	0.75	0.14
	0.9	0.10
	0.95	0.07
72	0.5	0.12
	0.75	0.10
	0.9	0.07
	0.95	0.05

13.7 Participant Enrollment and Follow-Up

A total of 260 subjects will be recruited for this trial, 10 subjects for Stage A, 90 subjects for Stage B, and 160 subjects for Stage C (80 each in C-1 and C-1b). The total estimated duration of recruitment of subjects to participate in this study is expected to be no less than 1 month and no greater than 1 year for each stage. (Stage A , B, and C-1 was completed prior to the amendment of this protocol.) Completion of Stage C-1b is expected to take approximately 3 months. Total involvement of an individual participant from administration of study product to final follow-up is expected to be no longer than 28 days.

Seven clinical sites have been selected to participate in this clinical study. Stages A and B were performed at one or more of the following Kathmandu campuses in Nepal: Lalitpur Nursing Campus, Sanepa, Kathmandu; The Central Department of Microbiology, Tribhuvan University, Kirtipur, Kathmandu; and The Institute of Medicine, Tribhuvan University, Maharajgunj, Kathmandu. Stages C-1 and C-1b will be performed at some or all of the above mentioned Kathmandu campuses and/or these additional campuses: AH, Patan Hospital TB Clinic, Lalgadh Leprosy Hospital, and Green Pastures Leprosy Hospital. Other campuses/hospitals may also be selected to participate.

13.8 Planned Interim Analyses

Interim analyses planned for this study are all directed at monitoring safety and the overall conduct of the trial. These analysis should not impact the Type I error, or equivalently the length of confidence intervals, for the primary computations of sensitivity and specificity at the end of the trial.

Members of the SMC will be composed of an independent group of physicians with relevant experience, including the ISM. The DMID PO and a Biostatistician will serve as non-voting members. The SMC will be convened by the DMID PO at the following time points:

- At the beginning of the study: A chair will be selected and the SMC will discuss procedures for study review.
- After the completion of Stage A: The SMC will submit a report to DMID, NHRC, and the CSU IRB for review and approval before Stage B is initiated.
- After the completion of Stage B: The SMC will submit a report to DMID, NHRC, and the CSU IRB for review and approval before Stage C is initiated.
- After the completion of Stage C-1: The SMC will submit a report to DMID, NHRC, and the CSU IRB.
- After the completion of Stage C-1b (the end of the study): The SMC will submit a report to DMID, NHRC, and the CSU IRB summarizing participant safety.
- In response to AE reporting, any SAEs deemed associated or not associated to the skin test antigens will be reviewed by the ISM and the DMID PO, who may convene the SMC. A report will also be submitted to the SMC for review in the event a number of AEs occur that are apparently associated with a particular antigen.

The primary charge to the advisory members is to monitor safety, study conduct, and study progress, as well as accumulated data. The committee also provides advice to DMID and the study investigators as to the appropriateness of continuing the study as designed. Monitoring bodies are advisory to DMID and their recommendations, while given careful consideration, are not binding.

Statistical interim analysis will be presented in tabular form, including but not limited to: listing demographic characteristics by subject ID and readers' assessments of erythema and measurements of induration; summary of skin test readings by subject ID, antigen code, response after ~15-minutes, ~30 minutes (for stage C1b only) and follow-up examination; and listing of all adverse events (including observations noted approximately at 15-30 minutes). Guidelines for stopping the study have been described in Section 10.2.2., "Criteria for Discontinuation of Study Intervention/Product for Withdrawal of a Subject (or a Cohort)".

13.8.1 Rules for Handling any SAE:

- The first reader will evaluate the skin test sites, measure induration and complete the Skin Test Reading Form. The second reader will then perform the same evaluation and measurements, and record the data. The MO, nurse or paramedic will then examine and interview the subject and complete the Follow-up Reaction Reading Form. Any of these staff members that become aware of an SAE will notify the attending physician.
- In the case that an SAE occurs, no further skin tests will be administered until the IRB has been informed.
- The attending physician will immediately examine the subject.
- The staff member and attending physician will complete the AE Form and the SAE Form; the principal investigator will be required to sign the SAE Form.

- The staff member is responsible for immediately reporting the SAE to the SC.
- The SC is responsible for verifying information and ensuring that all necessary information is obtained to clarify the event and determine its relevance to the study.
- The SC will notify the ISM no later than 24 hours after receiving the report of the SAE. The ISM will review the SAE upon notification of its occurrence.
- The SC or Principal Investigator will complete an SAE report and e-mail it to the DMID PO, DMID ORA, SMC, and the data center. In the event of a death, an autopsy report and terminal medical report should also be supplied. Updated reports generated by the SC must also be sent.
- The ISM will discuss any SAE that is possibly, probably, or definitely related to the study, and any death, whether related or not, with the DMID PO within 3 working days.
- The DMID PO will convene the SMC if necessary for further review.
- The Sponsor will then notify the investigator at CSU, the ORA, and the FDA.
- The SC will notify the NHRC, while the CSU investigator will notify the CSU IRB.
- Prior to reconvening the study, the SC must receive approval from the DMID PO.

13.8.2 Rules for Handling Multiple AEs:

Steps for handling AEs are similar to those outlined above for SAEs, but do not require the same level of urgency in reporting. AEs are identified by attending staff members and treated according to specifications outlined in the MOO. The attending physician is not notified unless the event becomes severe. The staff member completes the AE Form and informs the SC, who then informs the ISM. The ISM is responsible for reviewing all AEs and reporting concerns to the SC or Principal Investigator. If a pattern of AEs is indicative of a safety concern, the ISM can temporarily suspend enrollment of further subjects, pending discussion with the DMID PO and the SMC. This situation could pertain to the entire study, a specific study group, or individual study subjects, depending on the response to certain antigens and/or antigen dosages.

13.8.3 Safety Outcomes

The following safety outcome measures will be monitored: Measurement of induration, indication of erythema, measurement of local reactions, measurement of systematic reactions, and monitoring for AEs and SAEs. Each measure of safety is controlled by specifications for an AE and SAE. Monitoring safety outcome measures is performed during each study visit, up to day 3 for Stage A, and up to day 7 for Stages B and C. Reactions to the antigens that are assessed as being severe will be followed-up to day 28, or until resolved or stabilized. If a subject responds to any skin test antigen greater than 10mm in

diameter at any study visit, they will be followed-up to day 28, or until resolved or stabilized.

13.8.4 Statistical Rules to Halt Enrollment

N/A

13.9 Analysis Plan

Data for this study will consist of inclusion/exclusion documentation, medical histories used to determine leprosy and TB status, basic demographic information (age, race and gender), reactions to the antigens used to assess safety (erythema, itching, pain/tenderness, bleeding, urticaria, infection and blistering), induration as a measure of response for detecting leprosy, and adverse events. An important immunological addition will be the IFN-γ responses to at least the skin test antigens and Tuberculin/PPD, and the antibody response to the leprosy specific PGL-I antigen in participants in Stage C.

Study participants are randomized to different templates for antigen administration in order to add blinding to evaluation of outcomes and reduce observer bias. Analyses will be conducted to determine if there are important differences based on randomization within any study group. This factor will only be included in any of the subsequent analyses discussed below if there are clear suggestions of possible differences attributable to the randomization process.

Whenever appropriate, analyses will be conducted within study group and by combining groups that are similarly expected to have the same type, positive or negative, response to the test antigens.

13.9.1 Study Enrollment and Compliance

Descriptive analyses will be used to summarize the characteristics of the study cohorts and their completion of all study procedures. This will include basic demographic data and critical elements of the medical histories taken at the time of study enrollment, prior to antigen testing. Study participants are randomized to different templates for antigen administration in order to add blinding to evaluation of outcomes and reduce bias.

Sample size computations have assumed a 10% loss to follow-up, a generous estimate. Losses will be investigated to try and determine if there is a systematic bias of any type, but no imputation methods to adjust for losses are currently planned.

13.9.2 Safety

Safety analyses will summarize the proportion of study participants with erythema, itching, pain/tenderness, bleeding, urticaria, infection and blistering for each time point and antigen combination. (Reaction rates were very low in Stages A and B involving healthy controls, but may be higher in Stage C of this study.) If reaction rates are high, they will be compared across groups using categorical methods to test for statistically significant differences by group.

Longitudinal methods, such as a Generalized Estimating Equations (GEE) version of logistic regression will be considered as well.

Adverse Events will be coded by the Medical Dictionary for Regulatory Activities (MedDRA®) for preferred term and system organ class, and classified by their possible association with antigen testing. AEs will be tabulated by possible relationship to antigen and greatest reported intensity. They will be listed by subject identification code, antigen injection date, event description, MedDRA® preferred term, onset date and time, end date, maximal intensity, antigen association, action taken, outcome, resolution and brief notes. AEs will be tabulated by MedDRA® preferred term to determine if it is appropriate to conduct further analyses based on this classification.

13.9.3 Diagnostic Sensitivity/Specificity

These analyses will involve the selection of a cut-off for diagnostic testing, using the test antigens by dose, construction and comparison of ROC curves, and the determination of the sensitivity and specificity of the test antigens based on the selected cutoff points. This work will be based on the induration stimulated by the test antigens.

The text *Statistical Methods in Diagnostic Medicine* (37), will serve as a reference for most of the methods used in these analyses. An important assumption for many of these methods is that the distribution of the outcome measure (induration) follows an approximate binormal distribution, i.e. a combination of two normal distributions, one for the population with the condition of interest and another for the population without the condition. This assumption will be examined and exploited if it is met. Assuming that there is not a complete or virtually complete separation between the groups in their response, methods described by Schaefer, et.al. (38) will be examined to construct a cut-off for each of the antigens at each dose. For each cutoff the 95% confidence interval for the sensitivity and specificity based on this cutoff will be constructed. ROC curves will be constructed, the area under the curve and its 95% confidence interval computed and the antigens/doses will be compared to determine if there is a statistically significant difference in area.

13.9.4 Assay for Immune Responses

The IFN-γ responses and leprosy specific PGL-I antigen antibody responses will be assessed as possible diagnostic tools in a similar manner to the induration outcome. The antibody response, however, will be reported on a binary scale (positive/negative) so that there will be no opportunity to construct ROC curves or statistically determine a cut-off. Additional analyses will explore the correlation between the various outcome variables and test their association with baseline covariates such as demographic variables and medical history.

14 ACCESS TO SOURCE DATA/DOCUMENTS

The source documents for Stage A consisted of CRFs printed on two-ply No Carbon Required (NCR) paper. For Stages B and C, the source documents consist of data collection forms derived from the electronic case report forms (eCRF). Most data on the CRFs will be obtained directly from the participant; however, to obtain the necessary information for the purposes of the diagnosis of leprosy and tuberculosis, the necessary information will be obtained from the subject's medical record. These medical records will only be accessed following permission from the person concerned, and only the specified information will be obtained.

The SC will ensure the accuracy, completeness, legibility, and timeliness of the data reported on the source documents. For Stage A, the original (white copy) of the NCR source document was forwarded to the DCC for data entry; the second (yellow) copy of the NCR source document was maintained on-site at AH. For Stages B and C, the data from the source documents are entered into the eCRFs on a laptop computer at AH and sent to the DCC on a compact disk approximately monthly. The screening form will not be entered into the database, but will be retained in locked files on-site, in the event that data on these forms need to be analyzed in the future.

As a quality control measure, the data will be checked for anomalies and inconsistencies through the DCC's Data Integrity Evaluation System. The Data Manager will send a Data Resolution Form outlining missing values, out-of-range values, and other data anomalies to the central clinical site for reconciliation of data discrepancies. Photocopies of modified forms will be sent back to the DCC for entry into the database, while a photocopy will be maintained in the subject's CRF folder.

Essential trial documents will be maintained for this trial, in compliance with ICH E6 GCP, Section 8, and regulatory and institutional requirements for the protection of confidentiality of subjects. Files will be established by the sponsor, and by each investigator to organize and retain essential documents. To satisfy FDA IND requirements, essential documents will be maintained by DMID, AH, and Colorado State University for a period of 2 years after the end of the trial.

The DMID Office of Regulatory Affairs will inform the investigators as to when these documents no longer need to be retained. Each participating site will permit authorized representatives of DMID sponsors, FDA regulatory authorities, and either NHRC or CSU IRBs to examine study records for the purposes of quality assurance reviews, audits, and evaluation of the study safety and progress.

15 QUALITY CONTROL AND QUALITY ASSURANCE

A Manual of Operations (MOO) has been developed to provide a measure of quality control and quality assurance for the clinical trial. It is comprised of the following sections:

- Study Personnel
  - Staff Required (Qualifications and Duties)
  - Responsibilities (SC)
  - Responsibilities (Principal Investigator)
  - Individual's names, qualifications, and experience
- Study Structure SOPs
- Study Procedure SOP
- Data Entry and Analysis SOPs
- CRF Guidelines
- CRFs
- Study Logs

Since the clinical trial will take place at multiple centers, this document will be made available at each testing site for referral by study personnel in order to ensure the consistent management of quality in the trial.

16 ETHICS/PROTECTION OF HUMAN SUBJECTS

Evaluating new leprosy skin test antigens may provide a better way to diagnose leprosy in its early stages of infection. With early administration of drug therapy, infected individuals can be cured of this disease before nerve damage occurs. These tests may also help to break transmission of the disease through earlier detection. Participation in this study may not directly benefit the volunteers; however, the information gained about the early detection of individuals infected with leprosy should be beneficial to others with this affliction.

16.1 Declaration of Helsinki

The investigator will ensure that this study is conducted in full conformity with the current revision of the Declaration of Helsinki, or with the International Conference for Harmonisation Good Clinical Practice (ICH-GCP) regulations and guidelines, whichever affords the greater protection to the subject.

16.2 Institutional Review Board

Two institutions will be required to submit this protocol and respective informed consent forms to each of their ethics review committees for approval prior to proceeding with the clinical trial. The AH must receive approval from the NHRC, and CSU must receive approval from the CSU IRB. Multicenter sites in and around Kathmandu do not require additional IRB approval, because the NHRC approval includes these institutions. CSU IRB approval is required, because the leprosy skin test antigens were prepared in a Pilot Plant Facility at CSU, and the study was sponsored by DMID through this institution.

Amendments to the protocol or informed consent documents will be submitted to both NHRC and CSU IRB for review and approval before implementation. If a protocol deviation or violation occurs, the investigator is responsible to ensure that both HRCs are informed as per their requirements. In addition, following the completion of each stage, the safety report generated by the DCC, and the SMC report will be submitted to both IRBs. Approval must be obtained from both institution HRCs before proceeding to the following stage.

Participation by both institutions, CSU and Anandaban Leprosy Hospital, is allowable, because both have been issued a current U. S. Federal-Wide Assurance (FWA) number by the Office of Human Research Protection (OHRP). Additional testing sites in and around Kathmandu will be covered under the FWA issued to AH.

16.3 Informed Consent Process

Consent forms were drafted in the English language and translated into two of the appropriate languages (Nepali and Hindi), and back-translated into English to verify that all important elements were included. The appropriate form (in English, Nepali or

Hindi) will be presented to each subject. Each consent document includes the purpose of the research study, the applicable procedure, risks, benefits, compensation, confidentiality, statement of voluntary participation, who to contact with telephone numbers if the subject has problems or questions, and the subject, witness, and investigator signature section. Consent forms will be NHRC/CSU IRB approved. The subjects may withdraw consent at any time throughout the course of the trial. A copy of the consent document will be given to the subjects for their records. The rights and welfare of the subjects will be protected by emphasizing to them that the quality of their medical care will not be adversely affected if they decline to participate in this study, or if they withdraw from the study at any point after enrollment.

The following consent forms will be used in this clinical trial:

- Phase II, Practice Session
- Phase II, Stages A & B
- Phase II, Stage C (controls, contacts, TB)
- Phase II, Stage C (leprosy patients)
- Phase II, Medical Records

Qualified staff members involved in the training sessions will be asked to sign the Practice Session Informed Consent Form prior to beginning training.

Subjects recruited for Stages A, B and C will be informed of the study by a recruitment talk (in the case of healthy control subjects recruited on campuses) or individual explanation using a flip chart and the consent document (in the case of patients and patient contacts in leprosy/TB clinics) in their own language (Nepali or Hindi) by a senior member of the research team. In the case of illiterate subjects, the information will be read to them by a study staff member prior to the subject signing the consent document. Subjects will be encouraged to ask questions about concerns they may have with the study.

Leprosy index cases of healthy contacts of leprosy patients who are enrolled in the study will be asked to sign a Medical Records Consent Form, giving permission to the investigators to look at their medical record chart to obtain the following information: hospital number, hospital where treated, leprosy type, initial skin smear, treatment history, and months of treatment. The leprosy index case subject will also be asked the question, "How long has the healthy contact subject lived in the same house as you?"

On the day of the antigen administration procedure, volunteers will be asked to read and sign the applicable consent document (in English, Nepali or Hindi, as appropriate). This will be witnessed by a friend or staff member from the campus or institution, but not the investigator. The investigator (who can be anyone on the Anandaban team) will also sign the form. If the volunteer is illiterate, the investigator, who has read the form to the volunteer, must also complete the declaration at the bottom of the consent document.

16.4 Exclusion of Women, Minorities, and Children (Special Populations)

This study will include women, but exclude special minority populations, including children as previously described in Section 5.1.1, “Percentages of Women, Minorities and Children”.

16.5 Subject Confidentiality

Subject confidentiality is strictly held in trust by the participating investigators, their staff, and the study sponsors. This confidentiality is extended to cover testing of biological samples, in addition to the clinical information relating to participating subjects. The study protocol, documentation, data, and all other information generated will be held in strict confidence. Information concerning the study or the data will not be released to any unauthorized third party, without prior written approval of the sponsor.

Confidentiality is described to potential subjects during the recruitment talk, and mentioned again in the informed consent document. In summary, the subject will be informed that results from this research study may be published, but their name or identity will not be revealed. All documents and records required to be maintained by the investigator will be made available for review by the study monitor or other authorized representatives of the sponsor at CSU, FDA, DMID and NHRC. A copy of the signed consent document will be provided to each subject and the SC will keep the original forms in a locked file.

To maintain subject confidentiality, each volunteer who agrees to enter the study and signs a consent document, will be assigned a subject number. This number and the name of the volunteer will be recorded in a log, which will be kept in a locked file by the SC. The subject will only be identified by their subject number on all CRFs and DRFs, in order to protect their privacy. Participant identifiers such as name, phone number or address are not to be recorded on any CRF.

Blood samples will be labeled with only the subject number. Blood samples will be completely expended by the AH at the time of performing the cell mediated laboratory assay, while serum will be kept until completion of the study and used if necessary to repeat the Rapid PGL-I Antibody Test. Upon completion of the study, remaining serum will be autoclaved and discarded.

Both subject identifiers (the consent document and the screening log) will be kept in a locked file at AH by the SC, for a period of 2 years after the completion of the study. The DMID PO will inform the investigators as to when these documents no longer need to be retained.

16.6 Study Discontinuation

If the study is discontinued, subjects with a skin test reaction greater than 10mm will be asked to return on day 28 for examination. Further follow-up will continue until the reaction has been resolved. If the subject has experienced an SAE or AE caused by the study product, they will be treated with the appropriate care under medical supervision, and asked to return for follow-up visits until the symptoms of the event have resolved, or the subject's condition becomes stable. At the point of resolving reactions greater than 10mm and/or mild to severe adverse events, a Study Termination Form will be completed by the attending staff member. The study will continue until a Study Termination Form has been completed for each study participant. Since this study is testing a diagnostic reagent, a crossover to study drug for placebo recipients at the completion of this study is not applicable.

17 DATA HANDLING AND RECORD KEEPING

17.1 Data Management Responsibilities

Data collection is the responsibility of the clinical trial staff at AH under the immediate supervision of the SC, and overall supervision of the Principal Investigator. During the study, the Principal Investigator will maintain complete and accurate documentation for the study. All source documents and laboratory reports will be reviewed by the clinical team and data entry staff, who will ensure that they are accurate and complete. AEs must be graded at the time of occurrence, assessed for severity and causality, and reviewed by the site Principal Investigator or designee. SAE reports will be generated by the SC and reviewed by the PI, prior to sending to PPD Development Inc., DMIDs pharmacovigilance contractor, DMID Medical Monitor and the DMID PO.

Data collected at the clinical site will be checked at the DCC for anomalies and inconsistencies through the DCC's Data Integrity Evaluation System, as described in Section 14, "Access to Source Data/Documents". The DCC will be responsible for ensuring accuracy of data, analyzing the data, and preparing safety and sensitivity/specificity reports following each study stage. Additionally, data will be collected from the two laboratory assays. A compilation of this data will be sent to the DCC for inclusion in the sensitivity/specificity report for Stage C. Various reports are required throughout the study: (1) If an SAE occurs, the SC is responsible for generating a report from the SAE Form, to submit to the DMID PO, ORA, SMC and the DCC; (2) The DCC is responsible for generating a safety report following each study stage, for submission to the DMID PO and the SMC; (3) The SMC Chair is responsible for generating an SMC report summarizing the SMC recommendations following an *ad hoc* meeting to review an SAE, or following a study stage review; and, (4) The study PI is responsible for generating a final study report to submit to the DMID PO and ORA. The study sponsor will keep each report. Following the completion of the study, all data source documents, analysis files and reports will be transferred to the DMID PO for retention.

17.2 Data Capture Methods

Source documents will be the primary method for data collection for each individual study participant. For Stage A, the top copy of the NCR source document will be the CRF entered into the database. For Stages B and C, data will be captured on source documents derived from the eCRFs and entered into the eCRFs on the appropriate password-protected database system.

17.3 Types of Data

Data will be collected to measure safety and sensitivity/specificity. Data used to assess safety outcome measures will include induration and erythema; local and systemic reactions; and AEs and SAEs, while data used to assess sensitivity/specificity will include, induration and erythema.

17.4 Timing/Reports

Reviews and reports are periodic. Upon completion of each sequential stage, the DCC will analyze the data and compile a safety and/or sensitivity/specificity report, depending on the stage of the study under evaluation. This report will be submitted to the SMC for review. Following appraisal of the data by the SMC, the SMC Chair will summarize the committee's recommendations in a report to be submitted to DMID, NHRC and CSU IRB. Subject to approval from each of these authorities, the study will proceed to the next stage (no earlier than 4 days after the final reading of the previous stage), until the end of Stage C-1b. At this time, a final report will be submitted by the study PI to the study sponsor and both HRCs.

Safety and sensitivity/specificity outcome measures described in Section 17.3, "Types of Data", will be used to evaluate each study stage. In addition, data such as BCG scar size, age and gender, which is collected during the enrollment process, may also be used to evaluate study antigens. Tables reported by the DCC for appraisal of safety and sensitivity/specificity will include, but not be limited to: listing demographic characteristics; Listing of readers' assessments of erythema and measurement of induration; summary of skin test readings by antigen code (induration and erythema); summary of 15-minute reaction record and 30 minute reaction record (for State C1b only); summary of follow-up examination and reaction form; and listing of all AEs (including observations noted between 15-30 minutes after antigen administration for stage C1b) and SAEs. The safety report summarizing Stages A and B will be reviewed by the SMC to determine if skin test antigens, MLSA-LAM and MLCwA at the administered dosages, are safe to use in human subjects. Likewise, the safety report summarizing data from Stage C-1 will be reviewed before Stage C-1b begins. Subsequently, following Stage C-1b, the safety report will be analyzed according to plans outlined in Section 13.9, "Analysis Plan".

17.5 Provisions for Blinding

Two sets of randomization schemes will be created by the DCC to blind the data for each stage of the study. First, antigen codes will be randomized for each antigen and antigen dosage. Antigens will then be relabeled with assigned codes at the DMID-Clinical Agent Repository. Second, antigen administration templates will be randomized to prevent reader bias. Both randomization schemes will be sent to the clinical study PI,

in the event that unblinding is necessary. Antigen codes will be provided in separate envelopes, so that if only one antigen requires unblinding, the others will not be compromised. Unblinding antigens administered to one individual subject could unblind other study subjects and compromise the study. Moreover, there is no recognized medical benefit of knowing the identity of an antigen in order to treat a subject for an AE. Therefore, unblinding will not occur unless recommended by the SMC and approved by the DMID PO. In all cases of unblinding, the identity of antigens will only be made available to key study personnel as necessary during the study. To establish the safety of these antigens and antigen dosages, unblinding is only scheduled to occur following Stage B. .

17.6 Study Records Retention

All records pertaining to this study will be maintained by the clinical PI at AH, the study PI at CSU, and the DMID PO at DMID for a period of 2 years after the formal discontinuation of clinical development of the investigational product. Permission must be obtained from the DMID PO prior to destruction of these records.

17.7 Protocol Deviations

The trial will be conducted in compliance with this protocol when approved by the DMID sponsor, the FDA, the NHRC and CSU IRB. Protocol deviations or changes will not be made without prior review and approval from these authorities, except where necessary to eliminate an immediate hazard to trial subjects or when changes involve only logistical or administrative aspects of the trial, i.e., change of monitor or change in telephone number. Ensuring protocol compliance is the responsibility of all team members at Anandaban, CSU, EMMES Corporation, and DMID. Therefore, it is the responsibility of each team member within their area of expertise and contribution to this study, to ensure that a deviation from protocol does not occur. On the other hand, if a deviation does occur, it will be documented and forwarded to each authority listed above. If a change is implemented to eliminate an immediate hazard to trial subjects without prior approval, the deviation, reasons for it, and proposed protocol amendment, will be submitted for approval immediately.

18 PUBLICATION POLICY

The publication and authorship policy has been determined. Initiation of drafting a manuscript(s) from the results of this study will come from either the PI who initiated the study and/or the PI who was responsible for implementation of the study. Significant contributions towards the manuscript(s) will also come from the SC and DCC. Although these individuals are the major contributors, authorship should also extend to the key staff personnel at each institution involved. Therefore, including those individuals already mentioned, additional major contributors will also be assigned authorship, as deemed appropriate by both PIs.

19 LITERATURE REFERENCES

1. Anon. CTD (Control of Tropical Diseases) News. UNDP/WORLD BANK/WHO Special Programme for Research and Training in Tropical Diseases (TDR). TDR News. No. 45, p. 5, June 1994; also Anon. Weekly Epidemiological Record. World Health Organization (May 22, 1992) 67: 153-160, 1992; also Noordeen, S.K. Elimination of leprosy as a public health problem. State-of-the-art lecture, XIV Leprosy Congress, Orlando, FL (29 August - 4 September 1993). Int. J. Lepr. 62: 278-283, 1994; also Anon. Weekly Epidemiological Record. World Health Organization (June 5, 1998) 73: 169-176, 1998; also Anon. Weekly Epidemiological Record. World Health Organization (Oct. 2, 1998) 73: 305-312. 1998. http://www.who.int/topics/leprosy/en/

2. Noordeen, S.K. Eliminating leprosy as a public health problem; why the optimism is justified. Int. J. Lepr. 63: 559-566, 1995.

3. Smith, P.G. Revised estimates of global leprosy numbers. (Editorial.) Lepr. Rev. 63: 317-318, 1992; also Feenstra, P. Will there be a need for leprosy control services in the 21st century? (Paper presented by P. Feenstra at NSL press conference, Amsterdam, 27 January 1994). NSL (Netherlands Leprosy Relief) Assoc., Vol. 6. Pp. 1 and 8, May 1994. Lepr. Rev. 65: 297-299, 1994.

4. Brennan, P.J. Prospects for contributions from basic biological research to leprosy control. Int. J. Lepr. 63: 285-286, 1995.

5. Mitsuda, K. On the value of a skin reaction to a suspension of leprosy nodules. Jap. J. Derm. Urol. 19: 697-708, 1919.

6. Fernandez, J.M.M. The early reaction induced by Lepromin. Int. J. Lepr. 8: 1-14, 1940.

7. Dharmendra, D. The immunological skin tests in leprosy: the isolation of a protein antigen of Mycobacterium leprae. Ind. J. Med. Res. 30: 1-7, 1942.

8. Convit, J., M.E. Pinardi, J.L. Avila, and N. Atanzazu. Specificity of the 48-hour reaction to Mitsuda antigen. Bull. W.H.O. 52: 187-191, 1975.

9. Meyers, W.M., S. Kvernes, and C.H. Binford. Comparison of Reaction to human and armadillo Lepromins in leprosy. Int. J. Lepr. 43: 218-225, 1975.

10. Millar, J.W., C. Gannon, C.S. Chan. Comparison in leprosy patients of Fernandez and Mitsuda reactions using human and armadillo antigens. A double-blind study. Int. J. Lepr. 43: 226-233, 1975.

11. Krotoski, W.A., T.F. Mroczkowski, T.H. Rea, B.C. Clements, R.E. Neimes, M.K. Kahkonen, C.K. Job, and R.C. Hastings. Lepromin skin testing in the classification of Hansen's disease in the United States. Am. J. Med. Sci. 305: 18-24, 1993; Krotoski, W.A., T.F. Mroczkowski, E.J. Shannon, L.E. Millikan, R.M. Sanchez, and R.C. Hastings. Lepromin responses in recipients of a candidate anti-leprosy bacterin vaccine (WHO-IMMLEP

Mycobacterium leprae killed preparation in the USA). Int. J. Dermat. 32: 191-193, 1993.

12. IMMLEP Steering Committee 1982. Testing of purified armadillo-derived M. leprae in man. Document finalized by the IMMLEP Steering Committee at its meeting, 10-12 June 1981. World Health Organization. TDR/IMMLEP/SC/TEST/81.1.

13. Seibert, F.B. The isolation and properties of the purified protein derivative of tuberculin. Am. Rev. Tuberc. Pulmon. Dis. (Supplement) 30: 713-720, 1934.

14. Vaccination trials against leprosy: a meeting of the epidemiology subgroup of Scientific Working Group on the Immunology of Leprosy, Geneva, 11-13 February, 1985; TDR/IMMLEP/EDP/85.3: p. 7-8.

15. Draper, P. Protocol 1/79: Purification of M. leprae. Annex 1 of the Enlarged Steering Committee for Research on the Immunology of Leprosy (IMMLEP) Meeting of 7-8 February 1979. Geneva: World Health Organization, 1979, p. 4.

16. WHO (1977) Report of the Third IMMLEP Scientific Working Group on Leprosy. Protocol 3/77. WHO Document TDR/SWG/IMMLEP (3)/77, p. 20.

17. Gupte, M.D., and D.S. Anantharaman. Use of soluble antigens in leprosy epidemiology. Lepr. Rev. 59: 329-335, 1988.

18. Convit, J., C. Sampson, M. Zuniga, P.G. Smith, J. Plata, J. Silva, J. Molina, M.E. Pinardi, B.R. Bloom, and A. Salgado. Immunoprophylactic trial with combined Mycobacterium leprae/BCG vaccine against leprosy: preliminary results. The Lancet 339: 446-450, 1992.

19. Samuel, N.M., J.L. Stanford, R.J.W. Rees, T. Fairbain, and R.B. Adign. Human vaccination studies in normal and contacts of leprosy patients. Int. J. Lepr. 56: 36-44, 1984.

20. Gupte, M.D., D.S. Anantharaman, B. Nagaraju, S. Kannan, and R.S. Vallishayee. Experiences with Mycobacterium leprae soluble antigens in a leprosy endemic population. Lepr. Rev. 61: 132-144, 1988.

21. Hunter, S.W., M. McNeil, R.L. Modlin, V. Mehra, B.R. Bloom, and P.J. Brennan. Isolation and characterization of the highly immunogenic cell wall-associated protein of Mycobacterium leprae. J. Immunol. 142: 2864-2872, 1989.

22. Melancon-Kaplan, J. S.W. Hunter, M. McNeil, C. Stewart, R.L. Modlin, T.H. Rea, J. Convit, P. Salgame, V. Mehra, B.R. Bloom, and P.J. Brennan. Immunological significance of Mycobacterium leprae cell walls. Proc. Natl. Acad. Sci. USA 85: 1917-1921, 1988.

23. Collins, F.M., N.E. Morrison, and S.R. Watson. Fernandez and Mitsuda reactivity in guinea pigs sensitized with heat-killed Mycobacterium leprae: persistence and specificity of skin reactivity to soluble and particulate antigens. Int. J. Lepr. 51: 481-489, 1983; Watson, S.R., N.E. Morrison and F.M. Collins. Delayed hypersensitivity responses in mice and guinea pigs to Mycobacterium leprae, M. baccae, and M. nonchromogenicum cytoplasmic proteins. Infect. Immun. 25: 229-236, 1979.

24. Armadottir, T., H.L. Reider, A. Trebucq, and H.T. Waaler. Guidelines for conducting tuberculosis skin test surveys in high prevalence countries. Tubercle and Lung Disease 77: Suppl. 1-20, 1996.

25. Thole, J.E.R., B. Wielgs, J.E. Clark-Curtiss, T.H.M. Ottenhoff and T.F. Rinke de Wit. Immunological and functional characterization of Mycobacterium leprae protein antigens: and overview. Mol. Microbiol. 18: 791-800, 1995; Young, D.B., S.H.E. Kaufmann, P.W.M. Hermans, and J.E.R. Thole. Mycobacterial protein antigens: a compilation. Mol. Microbiol. 6: 133-145, 1992.

26. Pessolani, M.C.V., and P.J. Brennan. Mycobacterium leprae produces extracellular homologs of the antigen 85 complex. Infect. Immun. 60: 4452-4459, 1992.

27. Rivoire, B., M.C.V. Pessolani, C.M. Bozic, S.W. Hunter, S.A. Hefta, V. Mehra, and P.J. Brennan. Chemical definition, cloning, and expression of the major protein of the leprosy bacillus. Infect. Immun. 62: 2417-2425, 1994.

28. Mehra, V., B.R. Bloom, A.C. Bajardi, C.L. Grisso, P.A. Sieling, D. Alland, J. Convit, X. Fan, S.W. Hunter, P.J. Brennan, T.H. Rea, and R.L. Modlin. A major T cell antigen of Mycobacterium leprae is a 10-kD heat-shock cognate protein. J. Exp. Med. 175: 275-284, 1992.

29. Pessolani, M.C.V., D.R. Smith, B. Rivoire, J. McCormick, S.A. Hefta, S.T. Cole, and P.J. Brennan. Purification, characterization, gene sequence, and significance of a bacterioferritin from Mycobacterium leprae. J. Exp. Med. 180: 319-327, 1994.

30. Hunter, S.W., B. Rivoire, V. Mehra, B.R. Bloom, and P.J. Brennan. The major native protein of the leprosy bacillus. J. Biol. Chem. 265: 14065-14068, 1990.

31. Pessolani, M.C.V., and P.J. Brennan. Molecular definition and identification of new proteins of Mycobacterium leprae. Infect. Immun. 64: 5425-5427, 1996.

32. Marques, M.A.M., S. Chitale, P.J. Brennan, and M.C.V. Pessolani. Mapping and identification of the major cell wall-associated components of Mycobacterium leprae. Infect. Immun. 66: 2625-2631, 1998.

33. Marques, M.A.M., V.L. Antonio, E.N. Sarno, P.J. Brennan, and M.C.V. Pessolani. Binding of a2 laminins by pathogenic and non-pathogenic mycobacteria and adherence to Schwann cells. J. Med. Microbiol. 50: 23-28, 2001.

34. Marques,M.A.M., B.J. Espinosa, E.k. Xavier da Silveira, M.C.V. Pessolani, A.Chapeaurouge, J.Perales, K.M. Dobos, J.T. Belisle, J.S. Spencer, and P.J. Brennan. Continued proteomic analysis of Mycoacterium leprae subcellular fractions. Proteomics. 4: 1-12, 2004.

35. Ridley DS. Reactions in leprosy. Lepr Rev. 40: 77-81, 1969.

36. General Manual, National Tuberculosis Programme of Nepal, 2nd Edition, January 1997 (English); 4th Edition, April 2003 (Nepali), Department of Health Services, Ministry of Health, His Majesty's Government of Nepal, (prepared with WHO assistance, noted in the manuals' foreword).

37. Statistical Methods in Diagnostic Medicine, Zhou, Obuchowski and McClish, Wiley, 2002

38. Schaefer, Helmut, Constructing a Cut-Off Point for a Quantitative Diagnostic Test. Statistics in Medicine. 8: 1381-91, 1989.

Appendix A: Schedule of Procedures/Evaluations

				Follow-up Schedule						
Procedures		Recruitment	Screening & Intervention	Time Point 1	Time Point 2	Time Point 3		Time Point 4	Study Completion	Premature Discontinuation
Recruitment Talk		X								
Signed Consent Form			X							
Physical Exam	Complete		X							
	Follow-up		X	X	X	X		X	X	X
	Vital Signs		(X)	(X)	(X)	(X)		(X)	(X)	(X)
Clinical Laboratory	Pregnancy		X							
Research Laboratory	Blood Sampling		X							
Medical History			X							
Assessment of Eligibility Criteria			X							
Enrollment			X							
Study Intervention			X							
Skin Test Reading				X	X	X		X	X	X
Follow-up Interview				X	X	X		X	X	X
Assessment of Adverse Events				(X)	(X)	(X)		(X)	(X)	(X)
Review of Concomitant Medications				(X)	(X)	(X)		(X)	(X)	(X)

(X) – if needed

Time Points for Stage A:
Recruitment: Day –10 to –1
Baseline: Day 0
1 ~ 15 min
2 – 48h ± 3h
3 – 72h ± 3h
4 – Day 28 ± 3 days, if needed

Time Points for Stage B:
Recruitment: Day –10 to -1
Baseline: Day 0
1 ~ 15 min
2 – 72h ± 3h
3 – Day 7 ± 1 day
4 – Day 28 ± 3 days, if needed

Time Points for Stage C-1and C-1b:
Recruitment: Day –10 to -1
Baseline: Day 0
1 ~15 min;, ~30min (stage C1b only)
3 – Day 3 ± 1 day
4 – Day 7 ± 1 day
5 – Day 28 ± 3 days, if needed

APPENDIX B: IFN-GAMMA RESPONSES OF SENSITIZED T-CELLS EXPOSED TO DIFFERENT LEPROSY SKIN TEST ANTIGENS

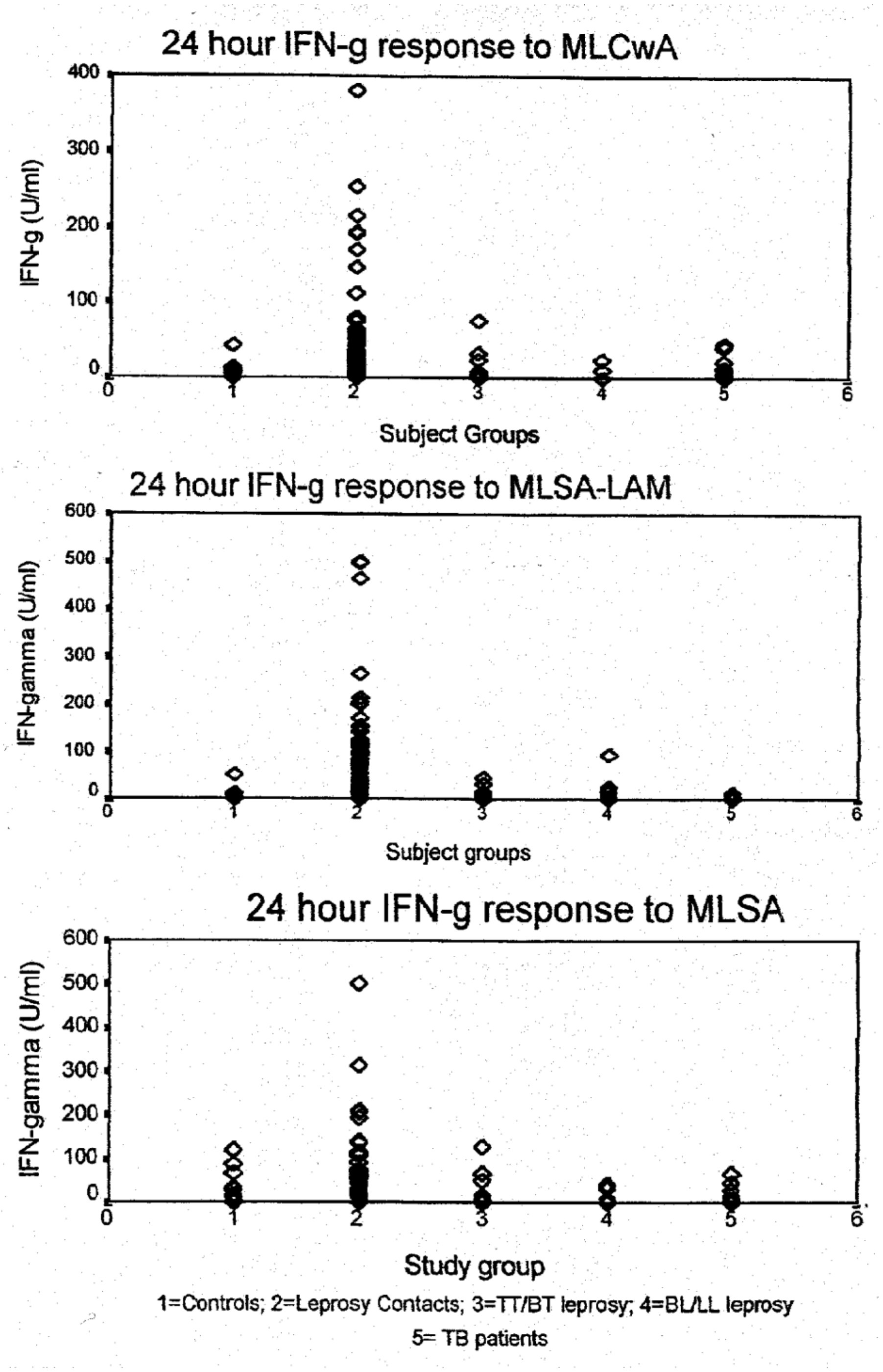

APPENDIX C: VOLUNTEER BREAKDOWN AND ESTIMATED TIME FOR COMPLETION OF TESTING

Study	Study Group	Number	Antigens Received	Study Site	Estimated Time
Stage A	Non-contacts	5	MLSA-LAM 0.1µg<br>MLSA-LAM 1.0µg<br>PPD<br>Saline	Kathmandu campuses	1 month
	Non-contacts	5	MLCwA 0.1µg<br>MLCwA 1.0µg<br>PPD<br>Saline		
Stage B	Non-contacts	45	MLSA-LAM 0.1µg<br>MLSA-LAM 1.0µg<br>PPD<br>Saline	Kathmandu campuses	1 month
	Non-contacts	45	MLCwA 0.1µg<br>MLCwA 1.0µg<br>PPD<br>Saline		
Stage C-1	Contacts	20	MLSA-LAM 1.0µg<br>MLCwa 1.0µg<br>PPD	Lalgadh Green Pastures Anandaban	5 months
	Leprosy patients	40<br>(20 TT/BT)<br>(20 BL/LL)		Lalgadh Green Pastures Anandaban	8 months
	Tuberculosis patients	20		Patan Hospital	1 month
Stage C-1b	Contacts	20	MLSA-LAM 0.1µg<br>MLCwa 0.1µg<br>PPD	Lalgadh Green Pastures Anandaban	5 months
	Leprosy patients	40<br>(20 TT/BT)<br>(20 BL/LL)		Lalgadh Green Pastures Anandaban	8 months
	Tuberculosis patients	20		Patan Hospital	1 month

Time	Stages A and B	Stage C
Day –10 to -1	Informational seminar and recruitment of volunteers	Informational seminar and recruitment of volunteers
Day 0	Pregnancy test for women	Pregnancy test for women
Day 0	Skin test antigens will be administered; first reading at ~15 minutes	Skin test antigens will be administered; readings at ~15 minutes and ~30 min (30 minute reading for stage C1b only).
Day 2	48 h reading, Part A only	none
Day 3	72 h reading	72 h reading
Day 7	7 d reading	7 d reading
Day 25 to 31	28 d reading for positive reactors	28 d reading for positive reactors

SIGNATURE PAGE

The signatures below constitute the approval of this protocol and the attachments, and provide the necessary assurances that this trial will be conducted according to all stipulations of the protocol, including all statements regarding confidentiality and according to local legal and regulatory requirements and to the principles outlined in applicable U.S. federal regulations and ICH guidelines.

Sponsor – NIH, NIAID, DMID, Bethesda, Maryland, USA

Signed: Robin M Mason Date: 3-4-09

Name: Robin M. Mason, M.S.
Title: Clinical Trials Specialist, Respiratory Diseases Branch, DMID

Principal Investigator - Anandaban Hospital, Kathmandu, Nepal

Signed: Min Thapa Date: 03.05.09

Name: Min Bahadur Thapa, M.B.B.S. (Nepal)
Title: Medical Officer

Principal Investigator – Department of Microbiology, Immunology & Pathology, Colorado State University, Fort Collins, Colorado, USA

Signed: [illegible] Date: 3-03-2009

Name: Patrick J. Brennan, Ph.D.
Title: University Distinguished Professor
